# Global burden of chronic kidney disease in adolescents and young adults, 1990–2019: a systematic analysis for the Global Burden of Disease Study 2019

**DOI:** 10.3389/fendo.2024.1389342

**Published:** 2024-09-18

**Authors:** Ping Sun, Xingyu Ming, Tiange Song, Yan Chen, Xin Yang, Zhaochen Sun, Xiaoxia Zheng, Luyao Tong, Zhiwei Ma, Zhengwei Wan

**Affiliations:** ^1^ Department of Health Management Center & Institute of Health Management, Sichuan Provincial People’s Hospital, University of Electronic Science and Technology of China, Chengdu, China; ^2^ School of Nutritional Sciences and Wellness, University of Arizona, Tucson, AZ, United States; ^3^ Department of Medical Records and Statistics, Sichuan Provincial People’s Hospital, University of Electronic Science and Technology of China, Chengdu, China; ^4^ Department of Laboratory Medicine, Sichuan Provincial People’s Hospital, University of Electronic Science and Technology of China, Chengdu, China; ^5^ School of Public Health, Southwest Medical University, Luzhou, China; ^6^ School of Life Science and Technology, University of Electronic Science and Technology of China, Chengdu, China; ^7^ Department of Urology, Sichuan Academy of Medical Sciences and Sichuan Provincial People’s Hospital, Chengdu, China

**Keywords:** chronic kidney disease, early-onset, adolescents and young adults, GBD 2019, global burden

## Abstract

**Background:**

The global status of chronic kidney disease (CKD) is underestimated, particularly the burden on adolescents and young adults (early-onset, aged 15–39).

**Objective:**

We aim to investigate the pattern and trend of early-onset CKD from 1990 to 2019.

**Methods:**

We analyzed age-specific rates of early-onset CKD incidence, death, and disability-adjusted life years (DALY) using Global Burden of Disease Study 2019 data. We examined the global, regional, national, gender-based, age group-based, and temporal changes of early-onset CKD burden from 1990 to 2019, as well as proportional DALY attributions of various risk factors.

**Results:**

From 1990 to 2019, the global age-specific incidence rate (per 100,000 population) significantly increased from 25.04 (95% confidence interval 18.51, 31.65) to 32.21 (23.73, 40.81) for early-onset CKD. However, the global age-specific death rate significantly decreased from 2.96 (2.76, 3.15) to 2.86 (2.61, 3.11), and the age-specific DALY rate remained stable. Regarding sociodemographic indexes (SDI), countries with middle SDI had the highest incidence rates and the fastest increasing trends, while those with low and low-middle SDI experienced the highest death and DALY rates. Women had a generally higher age-specific incidence rate than men, whereas men showed higher age-specific death and DALY rates. In addition, the burdens of CKD increased with age among adolescents and young adults. Moreover, the main attributable risk factors for DALY of early-onset CKD were high systolic blood pressure (SBP), fasting plasma glucose (FPG), and body mass index (BMI).

**Conclusion:**

The age-specific incidence rate of early-onset CKD increased significantly from 1990 to 2019, and the age-specific DALY rate remained stable. High SBP, high FPG, and high BMI were the primary risk factors. Targeted prevention and healthcare measures should be developed considering age, gender, and region.

## Introduction

Chronic kidney disease (CKD) is a pressing global health concern and a significant independent risk factor for cardiovascular disease, with over 650 million individuals affected by CKD in 2017 (1). CKD exhibits diverse etiologies and can manifest across all age groups, each influenced by distinct risk factors and disease profiles. Notably, the causes of death differ among middle-aged and elderly individuals with CKD (eg. renal failure), compared to adolescents and young adults with early-onset CKD (eg. cardiovascular causes and infections) (2).

Although less than 5% of adolescents with CKD progress to end-stage kidney disease, they still face a much higher death rate than that of healthy peers (3). Moreover, it has been demonstrated that the CKD burden appears to be increasing among young people (4), indicating that an up-to-date understanding of the global burden of early-onset CKD is essential for its primary intervention, secondary intervention, and health care. Adolescents and young adults grappling with CKD necessitate tailored healthcare interventions due to their unique set of complications, including hypertension, volume overload, metabolic bone disorders, and various physiological irregularities (5). Their clinical management often involves a complex array of interventions, ranging from polypharmacy for addressing bone disorders to specialized procedures like enteral feeding and catheterizations (6). Enhancing self-management skills and health literacy among this demographic is paramount, as limited health literacy is linked to unfavorable treatment adherence and outcomes (7).

Hence, a comprehensive understanding of the worldwide impact of early-onset CKD is imperative to allocate healthcare resources optimally across diverse regions. Despite existing reports on CKD deaths and disability-adjusted life years (DALY) between 1990 and 2017 (1), there remains a dearth of research specifically investigating the global burden of early-onset CKD (aged 15–39). Our research examines trends in age-specific incidence, death, and DALY rates of early-onset CKD, along with identifying attributable risk factors, across various demographic and sociodemographic categories from 1990 to 2019. This holistic analysis is essential for developing targeted primary and secondary interventions to achieve the United Nations Sustainable Development Goal of reducing premature mortality from non-communicable diseases by a third by 2030 (8).

## Methods

### Data sources

GBD 2019 project estimated the incidence, death, and DALY for 369 diseases and injuries across 204 countries and territories from 1990 to 2019. An overview of GBD 2019 data collection, modeling, and analysis was provided in the **Supplementary Materials** (**Appendix 1**). Data of cause-specific incidence rate, death rate, DALY rate, and DALY attributable to different risk factors (with corresponding 95% confidence intervals [CI]) of CKD, in adolescents and young adults from 1990 to 2019, by age, sex, year, region, and nations were obtained using the Global Health Data Exchange GBD results tool (http://ghdx.healthdata.org/gbd-results-tool).

### Relevant definitions

CKD was defined by elevated urinary albumin to creatinine ratio, decreased estimated glomerular filtration rate, or end-stage kidney disease (1). Our study specifically focused on early-onset CKD, characterized by an age of onset between 15 and 39 years. DALY is a comprehensive measure of disease burden, combining years of life lost due to premature death and years lived with disability. The sociodemographic index (SDI) serves as an indicator of a region’s development status, encompassing factors such as fertility rates among females under 25, average years of education for individuals aged 15 and above, and lag-distributed income per capita. 204 countries and territories were categorized into quintiles based on SDI level: low, low-middle, middle, high-middle, and high. Temporal trends were described using the estimated annual percentage change (EAPC) (9). Positive EAPC indicated an increasing incidence/death/DALY rate, while negative suggested a decline in the rates.

### Statistical analysis

We evaluated the number and the age-specific rates of incidence, death, and DALY in 2019, and their EAPCs (95% CI) (1990–2019) associated with early-onset CKD. We quantified age-specific trends in CKD burden based on gender and age group with a 5-year interval. Differences between males and females were calculated using the age-specific rates in males minus that in females. Based on the GBD 2019 country-level data, we mapped the age-specific incidence rates, death rates, DALY rates, and their EAPCs for 204 countries and regions. Additionally, we depicted the relationship between the SDI and the incidence/death/DALY rate by GBD region using Gaussian process regression. Furthermore, Spearman’s correlation coefficients between the SDI and EAPCs of the age-specific incidence/death/DALY rate were calculated. All statistical analyses and plots were implemented using the R program (Version 4.1.2). A two-tailed *p*-value < 0.05 was considered statistically significant.

## Results

### Global burden of early-onset CKD in 2019

In 2019, the global incidence, death, and DALY rates (per 100,000 population) for early-onset CKD were 32.21 (95% CI 23.73 to 40.81), 2.86 (2.61 to 3.11), and 236.85 (209.03 to 268.91), respectively. The global incidence and death rates were slightly higher in females (34.33) than in males (30.14), whereas the death and DALY rates were higher for men (3.32 and 253.82, respectively) than for women (2.38 and 219.45, respectively) (**Table 1**). Age-specific incidence, death, and DALY rates all increased with age groups (**Supplementary Figure 1**).

**Table 1:** Age-specific incidence, death, and DALY rates (per 100,000 persons) of the early-onset CKD in adolescents and young adults, along with their EAPCs globally, categorized by sex, SDI categories, and GBD regions from 1990 to 2019.

Location name	Incidence rate			Death rate			DALYs rate		
	1990 (per 100k)	2019 (per 100k)	EAPC (%)*	1990 (per 100k)	2019 (per 100k)	EAPC (%)*	1990 (per 100k)	2019 (per 100k)	EAPC (%)*
Global	25.04 (18.51, 31.65)	32.21 (23.73, 40.81)	0.98 (0.95, 1.02)	2.96 (2.76, 3.15)	2.86 (2.61, 3.11)	-0.40 (-0.56, -0.24)	230.83 (208.7, 255.62)	236.85 (209.03, 268.91)	-0.05 (-0.16, 0.06)
Male	22.62 (16.48, 28.6)	30.14 (22.32, 38.15)	1.08 (1.05, 1.10)	3.17 (2.91, 3.44)	3.32 (3.05, 3.67)	-0.04 (-0.17, 0.09)	232.6 (209.3, 260.57)	253.82 (225.76, 287.52)	0.19 (0.09, 0.30)
Female	27.53 (20.37, 34.8)	34.33 (25.26, 43.5)	0.90 (0.87, 0.93)	2.74 (2.48, 2.98)	2.38 (2.1, 2.64)	-0.87 (-1.08, -0.65)	229.02 (203.46, 258.05)	219.45 (187.72, 252.02)	-0.32 (-0.45, -0.19)
High SDI	18.12 (12.26, 24.63)	22.97 (15.94, 30.37)	0.73 (0.68, 0.77)	0.76 (0.74, 0.79)	0.8 (0.72, 0.91)	0.47 (0.28, 0.67)	86.63 (72.51, 104.16)	95.66 (77.95, 117.88)	0.52 (0.45, 0.58)
High-middle SDI	25.29 (18.12, 32.57)	31.7 (22.42, 41.57)	0.99 (0.89, 1.08)	2.13 (1.97, 2.3)	1.42 (1.3, 1.54)	-2.15 (-2.44, -1.85)	177.37 (156.58, 200.72)	143.16 (120.07, 168.96)	-1.03 (-1.17, -0.88)
Middle SDI	26.39 (19.43, 33.31)	37.49 (27.3, 47.6)	1.30 (1.28, 1.33)	3.71 (3.45, 4)	3.23 (2.99, 3.5)	-0.85 (-1.03, -0.67)	282.77 (255.22, 313.04)	271.69 (240, 307.31)	-0.33 (-0.44, -0.22)
Low-middle SDI	28.45 (21.43, 35.24)	33.51 (25.23, 42.11)	0.68 (0.61, 0.75)	3.68 (3.31, 4.07)	3.76 (3.35, 4.17)	-0.09 (-0.31, 0.13)	277.11 (247.55, 310.36)	290.52 (257.55, 326.58)	0.08 (-0.10, 0.26)
Low SDI	22.94 (17.38, 28.22)	26.43 (20.04, 32.82)	0.64 (0.56, 0.72)	4.1 (3.6, 4.61)	3.78 (3.28, 4.29)	-0.30 (-0.38, -0.22)	297.86 (264.34, 331.83)	288.27 (250.44, 327.99)	-0.12 (-0.18, -0.06)
East Asia	19.01 (13.44, 24.52)	21.34 (14.33, 28.71)	0.63 (0.51, 0.75)	2.83 (2.43, 3.28)	1.49 (1.27, 1.72)	-3.11 (-3.62, -2.60)	216.86 (186.75, 248.94)	138.82 (114.95, 165.43)	-1.82 (-2.12, -1.52)
South Asia	31.93 (24.26, 39.56)	33.01 (24.86, 41.61)	0.29 (0.19, 0.39)	3.42 (2.9, 3.9)	3.73 (3.23, 4.26)	0.13 (-0.18, 0.45)	259.45 (222.69, 296.9)	280.7 (245.22, 319.24)	0.22 (-0.07, 0.50)
Southeast Asia	29.12 (21.77, 36.21)	39.63 (29.77, 49.81)	1.07 (1.05, 1.10)	5.77 (5.23, 6.41)	4.87 (4.32, 5.49)	-0.81 (-0.91, -0.70)	423.57 (382.04, 475.41)	397.09 (346.97, 450.76)	-0.40 (-0.49, -0.32)
Central Asia	50.13 (39.22, 61.49)	68.14 (52.61, 84.48)	1.22 (1.08, 1.35)	4.06 (3.77, 4.48)	4.67 (4.07, 5.35)	-0.56 (-0.99, -0.13)	319.59 (288.26, 358.36)	375.47 (323.81, 430.05)	-0.21 (-0.51, 0.09)
High-income Asia Pacific	21.2 (14.69, 28.37)	22.14 (15.23, 29.73)	0.28 (0.15, 0.41)	0.93 (0.88, 0.98)	0.29 (0.27, 0.31)	-3.99 (-4.21, -3.77)	86.36 (76.52, 97.81)	49.31 (39.34, 61.82)	-1.90 (-2.04, -1.75)
Oceania	34.2 (26.35, 41.87)	40.11 (30.88, 49.23)	0.41 (0.34, 0.48)	4.25 (3.51, 5.02)	4.51 (3.66, 5.68)	-0.02 (-0.26, 0.22)	334.4 (280.81, 396.92)	371.71 (306.63, 453.44)	0.16 (-0.03, 0.34)
Australasia	11.34 (7.02, 16.14)	15.31 (9.74, 21.57)	0.86 (0.76, 0.97)	0.31 (0.29, 0.34)	0.32 (0.28, 0.36)	0.03 (-0.13, 0.19)	45.31 (36.29, 58.55)	50.12 (38.46, 63.84)	0.18 (0.06, 0.30)
Eastern Europe	41.99 (29.69, 54.53)	62.88 (45.32, 83.31)	1.62 (1.37, 1.86)	1.64 (1.59, 1.7)	1.15 (1.02, 1.28)	-2.63 (-3.12, -2.13)	155.6 (134.25, 182.71)	138.5 (109.54, 174.78)	-1.16 (-1.45, -0.87)
Western Europe	11.05 (6.87, 15.75)	12.15 (7.82, 17.2)	0.28 (0.21, 0.36)	0.39 (0.37, 0.4)	0.23 (0.22, 0.25)	-2.01 (-2.15, -1.87)	53.39 (42.74, 67.17)	43.96 (33.74, 57.12)	-0.81 (-0.87, -0.74)
Central Europe	27.94 (19.89, 36.24)	36.4 (26.17, 48.04)	1.06 (0.95, 1.17)	1.61 (1.56, 1.66)	0.81 (0.7, 0.94)	-2.40 (-2.74, -2.07)	133.47 (119.6, 150.85)	93.29 (76.04, 114.32)	-1.16 (-1.41, -0.91)
High-income North America	20.15 (13.28, 28.48)	18.48 (12.16, 25.1)	-0.67 (-0.84, -0.51)	0.77 (0.75, 0.79)	0.93 (0.87, 0.98)	1.06 (0.92, 1.21)	99.62 (80.47, 122.94)	109.57 (90.99, 132.97)	0.51 (0.34, 0.68)
Andean Latin America	18.79 (13.49, 24.35)	31.14 (22.31, 40.32)	1.74 (1.69, 1.79)	3.4 (3.06, 3.77)	3.03 (2.41, 3.76)	-0.66 (-1.05, -0.28)	253.94 (226.93, 283.13)	255.88 (208.87, 307.02)	-0.19 (-0.49, 0.11)
Central Latin America	35.91 (26.56, 45.91)	61.37 (46.39, 76.24)	1.71 (1.59, 1.83)	3.33 (3.25, 3.42)	5.18 (4.56, 5.86)	1.79 (1.69, 1.89)	292.19 (260.09, 332.24)	449.54 (387.8, 519.44)	1.68 (1.61, 1.76)
Caribbean	29.19 (21.9, 36.77)	46.37 (34.53, 58.34)	1.45 (1.26, 1.64)	3.11 (2.75, 3.56)	4.2 (3.36, 5.18)	1.13 (1.06, 1.21)	257.24 (225.04, 298.73)	354.05 (292.18, 421.64)	1.17 (1.13, 1.22)
Tropical Latin America	28.76 (20.79, 36.81)	37.42 (26.69, 48.07)	0.75 (0.65, 0.86)	2.95 (2.84, 3.06)	1.78 (1.69, 1.87)	-1.94 (-2.25, -1.64)	232.87 (211.66, 258.9)	175.32 (150.1, 207.14)	-1.14 (-1.36, -0.92)
Southern Latin America	15.56 (10.4, 20.89)	19.18 (13.11, 25.51)	0.83 (0.79, 0.87)	1.71 (1.59, 1.82)	1.47 (1.31, 1.66)	-0.70 (-0.84, -0.55)	143.33 (127.25, 161.37)	133.23 (114.15, 154.14)	-0.31 (-0.38, -0.23)
Eastern Sub-Saharan Africa	14.11 (10.12, 18.05)	15.33 (10.97, 19.88)	0.43 (0.33, 0.53)	4.19 (3.45, 4.8)	3.25 (2.68, 3.83)	-1.06 (-1.12, -0.99)	302.76 (254.95, 344.28)	252.13 (213.4, 292.44)	-0.77 (-0.83, -0.72)
Southern Sub-Saharan Africa	34.69 (25.96, 43.43)	42.61 (31.68, 53.32)	0.66 (0.41, 0.91)	4.47 (4, 5.11)	4.52 (3.59, 5.44)	-0.31 (-1.13, 0.52)	319.09 (283.23, 361.91)	335.3 (272.77, 401.31)	-0.07 (-0.71, 0.56)
Western Sub-Saharan Africa	27.3 (20.74, 33.56)	32.79 (24.95, 40.46)	0.93 (0.67, 1.18)	4.89 (4.03, 5.97)	4.34 (3.37, 5.34)	-0.36 (-0.41, -0.30)	346.89 (289.3, 414.73)	327.18 (265.01, 393.23)	-0.15 (-0.20, -0.11)
North Africa and Middle East	25.78 (18.52, 33.24)	47.97 (34.81, 60.75)	2.12 (2.09, 2.15)	2.49 (2.22, 2.77)	2.01 (1.64, 2.46)	-0.86 (-0.99, -0.73)	208.08 (184.15, 235.89)	205.76 (168.46, 251.78)	-0.10 (-0.20, 0.00)
Central Sub-Saharan Africa	15.29 (11.03, 19.48)	18.37 (13.04, 23.7)	0.70 (0.64, 0.76)	4.51 (3.61, 5.44)	3.57 (2.69, 4.5)	-0.86 (-0.91, -0.81)	320.88 (263.95, 383.09)	268.45 (211.58, 329.28)	-0.66 (-0.71, -0.62)

*If the EAPC and its lower boundary of the 95% CI were positive, the rate was considered to increase. Otherwise, if the EAPC and its upper boundary of the 95% CI were negative, the rate was considered to decline.

**Figure 1:**
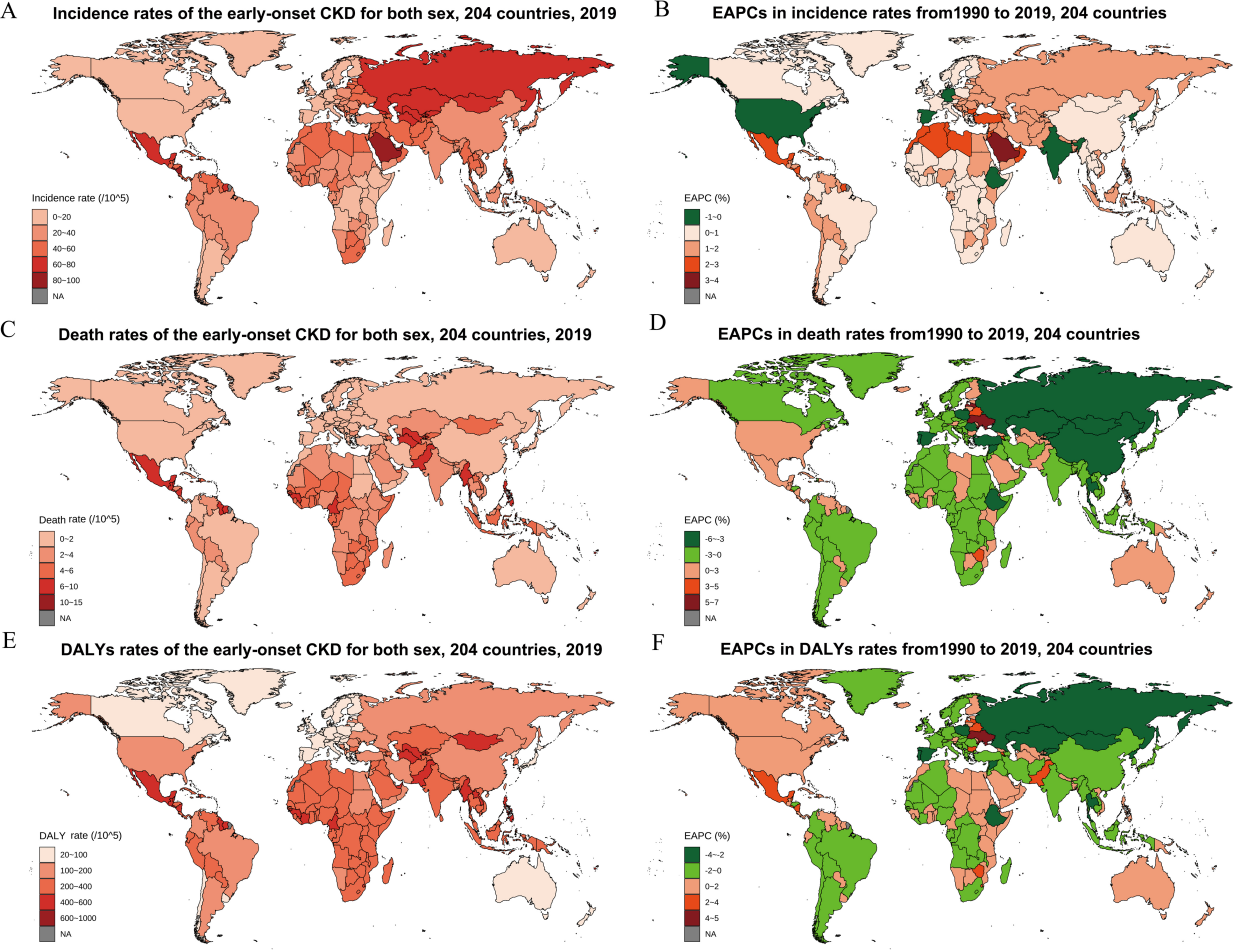
The global burden of early-onset CKD for both sexes in 204 countries and territories. **(A)** Incidence rate in 2019; **(B)** The EAPCs in incidence rates from1990 to 2019; **(C)** Death rate in 2019; **(D)** The EAPCs in death rates from1990 to 2019; **(E)** DALY rate in 2019; **(F)** The EAPCs in DALY rates from 1990 to 2019. CKD, chronic kidney disease; EAPC, estimated annual percentage change; DALY, disability-adjusted life year.

By SDI category, the highest incidence rate was in middle SDI countries (37.49, 27.3 to 47.6). Low and low-middle SDI countries had the highest death rate (3.78, 3.28 to 4.29) and DALY rate (290.52, 257.55 to 326.58), respectively (**Table 1**). The lowest incidence, death, and DALY rates were found in high SDI countries, with 22.97, 0.8, and 95.66 per 100, 000 population, respectively (**Table 1**). At the regional level, the greatest incidence rate of early-onset CKD was seen in parts of Central Asia, whereas parts of Central Latin America had the highest death and DALY rates (**Table 1**). At the country-level analysis, six nations (Palau, Micronesia, Kiribati, Nauru, El Salvador, and Solomon Islands) demonstrated consistent presence within the top 10 ranks for incidence, death, and DALY rates in 2019 (**Figure 1**; **Supplementary Tables 1**–**3**).

**Figure 2:**
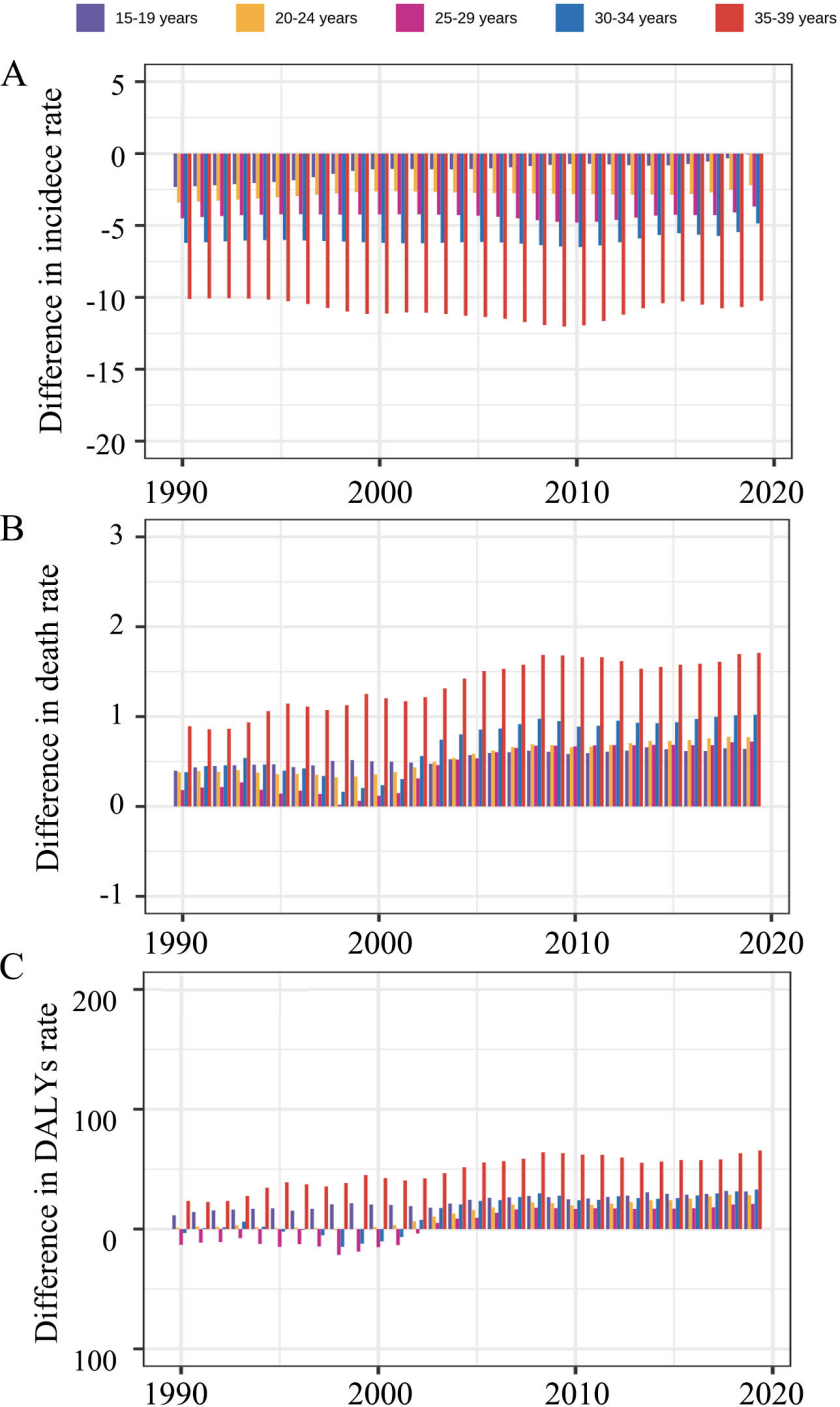
The difference in **(A)** incidence rate, **(B)** death rate, and **(C)** DALY rate between men and women by age groups globally, from 1990 to 2019. The difference was calculated using the age-specific rate in males minus that in females, with a difference higher than 0 meaning men have higher rates. DALY, disability-adjusted life year.

The differences in age distribution in incidence, death, and DALY rates between men and women were analyzed by age groups and SDI categories. Globally, the differences in incidence, death, and DALY rates between men and women were predominantly attributable to the older age groups (35–39 years) (**Figure 2**). Among all age groups, females had a higher incidence compared with males, and this was reversed in death and DALY with a few exceptions in the early years. Regarding SDI categories, females were estimated to have higher incidence rates than males for all the age groups in all the SDI categories, except for the 15–19 age group in high- and low-SDI countries (**Supplementary Figure 2**). The sex difference was mainly attributed to the 15–19 years age group for death and DALY rates in low SDI countries (**Supplementary Figures 3**, **4**).

### Temporal trend of early-onset CKD from 1990 to 2019

The global numbers of incidence, death, and DALY of CKD in adolescents and young adults have risen during the past 30 years (**Supplementary Table 4**). Of those, the incidence increased from 5.49 (95% CI 4.06 to 6.94) thousand in 1990, to 9.56 (7.04 to 12.11) thousand in 2019, an increase of 74.13%, markedly higher than the percentage increases for death (30.68%) and DALY (38.83%) numbers.

**Figure 3:**
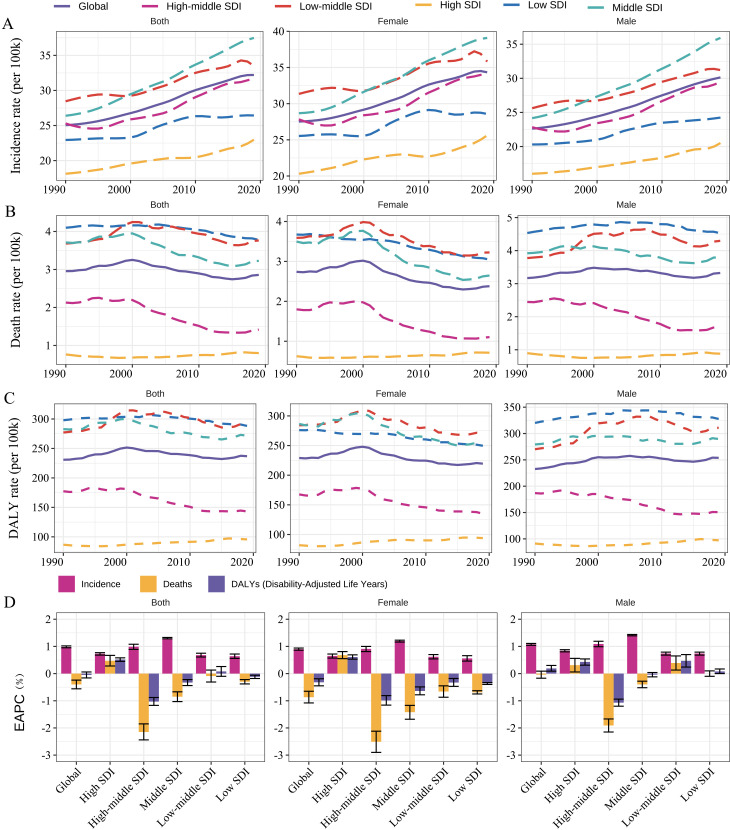
Temporal trend of age-specific **(A)** incidence rate, **(B)** death rate, and **(C)** DALY rate for the early-onset CKD globally and by SDI and sex, respectively, from 1990 to 2019. Panel **(D)** show EAPCs of age-specific incidence, death, and DALY rates by SDI and sex, respectively, from 1990 to 2019. DALY, disability-adjusted life year; CKD, chronic kidney disease. EAPC, estimated annual percentage change.

The incidence rate increased from 1990 to 2019 globally and in all SDI regions (**Figure 3A**). Detailly, the global incidence rate increased from 25.04 (95% CI 18.51 to 31.65) in 1990, to 32.21 (23.73 to 40.81) in 2019, with an EAPC of 0.98% (0.95% to 1.02%) (**Table 1**; **Figure 3D**). Middle SDI Countries exhibited the fastest increase in incidence rate (EAPC 1.30%, 95% CI 1.28% to 1.33%), whereas the EAPC was 0.64% to 0.99% for the other four SDI categories (**Table 1**; **Figures 3A, D**). Consistent increasing trends were observed in incidence rates in all age groups globally and by SDI categories and sex (**Supplementary Figures 5A**, **6A**, **7A**). All 21 regions exhibited an increase in incidence rate, except High-income North America, which had an EAPC of -0.67% (95% CI -0.84% to -0.51%) (**Table 1**). North Africa and Middle East had the fastest increase in incidence rate (EAPC 2.12%, 95% CI 2.09 to 2.15) for early-onset CKD between 1990 and 2019, followed by Andean Latin America (1.74%), Central Latin America (1.71%), and Caribbean (1.45%) (**Table 1**). At the country level, 194 of 204 countries exhibited an estimated increasing temporal trend in incidence rate (**Figure 1B**; **Supplementary Table 1**). Saudi Arabia, Bahrain, and Morocco had the fastest increases with an EAPC of 3.06%, 2.81%, and 2.71%, respectively (**Figure 1B**; **Supplementary Table 1**).

Conversely, there was a significant temporal decreasing trend in death rate (EAPC -0.40%, 95% CI -0.56% to -0.24%) and a non-significant decrease in DALY rate (-0.05%, -0.16% to 0.06%) at the global level from 1990 to 2019 (**Table 1**; **Figures 3B–D**). The death rate and DALY rate showed the fastest declines in countries with high–middle SDI (**Table 1**; **Figure 3D**). There was consistent geographical region variation in the temporal changes of death rate and DALY rate (**Table 1**). For example, most regions exhibited declines in the death rate and DALY rate over time (e.g., East Asia, High-income Asia Pacific, and Eastern Europe) whereas three regions (Central Latin America, Caribbean, and Australasia) showed significant increasing trends, and four regions (South Asia, Oceania, Australasia, and Southern Sub-Saharan Africa) had non-significant changed trends (**Table 1**). Among the 204 countries included in the study, 84 and 140 had an estimated increasing temporal trend in death rate and DALY rate, respectively (**Figures 1D, F**; **Supplementary Tables 2**, **3**). Ukraine had the highest increasing trends of DALY rate (EAPC 4.07%) and death rate (6.85%) from 1990 to 2019. Regarding age groups, the death and DALY rates consistently decreased over time in all age groups, especially in high-middle or middle SDI countries (**Supplementary Figures 5B, C**, **6B, C**, **7B, C**). However, significant increasing trends were found in 25–39 age groups within high SDI countries. Furthermore, in low or low-middle SDI countries, females exhibited significant temporal decreasing trends in death and DALY rates for early-onset CKD in all age groups, whereas males had a significant temporal increase in the rates in the 25–39 years group.

### Association of early-onset CKD burden with SDI

**Figure 4:**
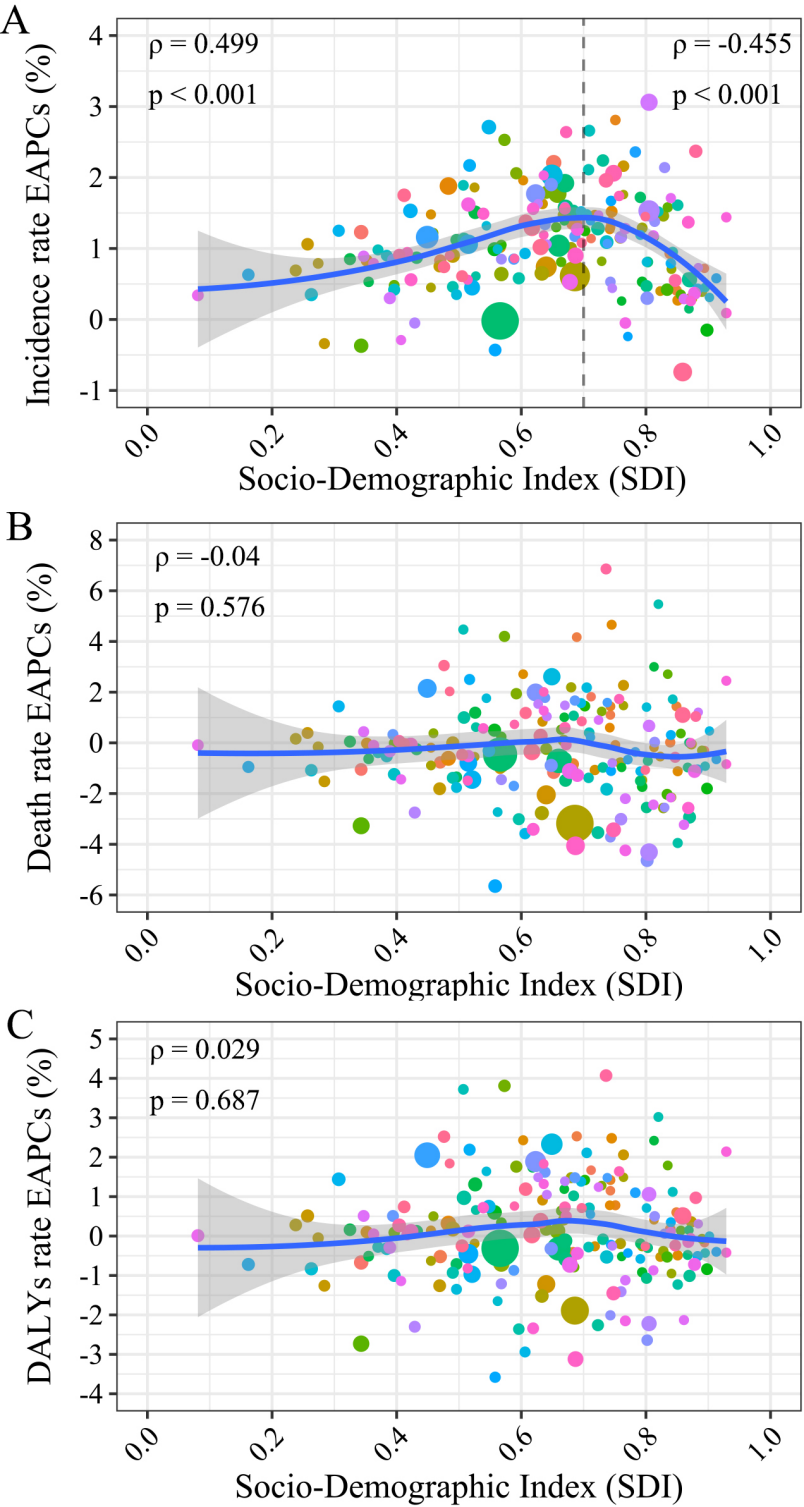
The correlation between the EAPCs of incidence/death/DALY and the SDI in 2019. The size of the circle is increased with the number of incidence, death, and DALY cases of early-onset CKD. **(A)** The EAPCs of incidence rate; **(B)** The EAPCs of death rate; **(C)** The EAPCs of DALY rate. EAPC, estimated annual percentage change; DALY, disability-adjusted life year; SDI, socio-demographic index; CKD, chronic kidney disease.

The non-linear association between the SDI and regional incidence, death, and DALY rates was examined using Gaussian process regression (**Supplementary Figure 8**). It resembled an asymmetric inverted V-shape association between the SDI and early-onset CKD incidence rates (**Supplementary Figure 8A**). The death and DALY rates both decreased with SDI levels (**Supplementary Figures 8B, C**). In terms of the correlation between the EAPCs and SDI (2019) at the national level, an asymmetric inverted V-shape was observed between EAPC for incidence rates and SDI (2019) (**Figure 4A**). The fitted curve peaked when the SDI approached 0.70, with a significant positive association found (ρ = 0.449, *p* < 0.001) when the SDI was limited to below 0.70, and a significant negative correlation observed (ρ = -0.455, *p* < 0.001) for SDI above 0.70. No apparent correlation was found in death and DALY in adolescents and young adults (ρ for death -0.04, *p* = 0.576; ρ for DALY 0.029, *p* = 0.687) (**Figures 4B, C**).

### Attributable risk factors for DALY in early-onset CKD

**Figure 5:**
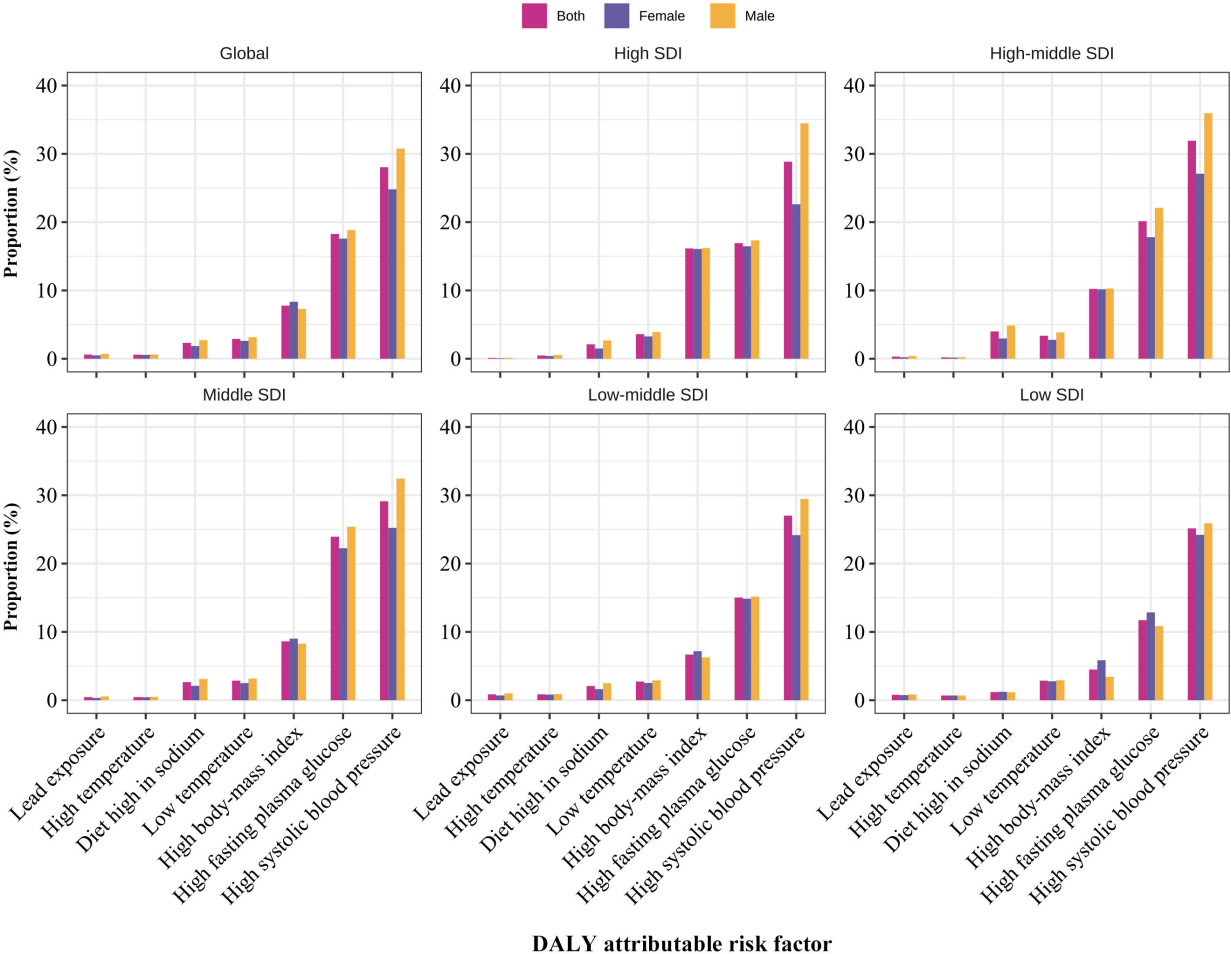
The proportion of early-onset CKD DALYs attributable to 7 risk factors classified by sex and SDI regions, respectively in 2019. DALY, disability-adjusted life year; CKD, chronic kidney disease.

Globally, DALY for CKD can be attributed to seven risk factors including lead exposure, high temperature, diet high in sodium, low temperature, high body-mass index (BMI), high fasting plasma glucose (FPG), and high systolic blood pressure (SBP) (**Figure 5**; **Supplementary Table 5**). In 2019, high SBP was the highest attributable risk factor with a proportion of 28.03%, followed by high FPG (18.27%), and high BMI (7.78%). Similar exhibits were observed in different regions divided by SDI, and were mostly consistent in males and females (**Figure 5**; **Supplementary Table 5**). By sex, the proportions of most of the global attributable risk factors in males were markedly higher than those in females, except for the high BMI (proportion for female 8.34%, for male 7.30%). The contribution of different risk factors varied with age groups (**Supplementary Figure 9**). First, high SBP and high FPG were the primary factors for all age groups. Second, high BMI did not contribute to early-onset CKD except in the age group of 35–39 years. Third, for the populations in the 15–19 and 20–24 years groups, only four risk factors (high SBP, high FPG, low temperature, and high temperature) were attributable for the CKD. In addition, the proportion of risk factors contributing to early-onset CKD that was attributable to high SBP and high BMI both increased while the proportion of high FPG decreased from 1990 to 2019 (**Supplementary Figure 10**).

## Discussion

This study provides a comprehensive estimation of the burden of CKD among adolescents and young adults. The primary findings are as follows: first, from 1990 to 2019, the global age-specific incidence rate of early-onset CKD showed a significant upward trend, while the death rate exhibited a notable downward trend, and the DALY rate remained stable. Second, in 2019, countries with a middle SDI had the highest incidence rates and the fastest increasing trend in incidence. Low and low-middle SDI countries had the highest death and DALY rates for CKD, while countries with a high-middle SDI experienced the fastest decline in death and DALY rates. Third, the global age-specific incidence rate is higher in women than in men, whereas the death rate and DALY rate are higher in men. Fourth, the burdens of CKD were proportional to age in adolescents and young adults. Fifth, the most contributive risk factors for early-onset CKD were high SBP, high FPG, and high BMI, and patterns of all attributable risk factors varied across regions, age groups, and sexes.

From 1990 to 2019, the global age-specific incidence rate of early-onset CKD exhibited a significant upward trend, while the age-specific death rate showed a marked decline. Researches indicate that changes in lifestyle and environmental factors (such as unhealthy diets and rising obesity rates), and advancements in medical diagnostic technologies contribute to the higher incidence rate of early-onset CKD (10, 11), indicating that the relevant institutions should prioritize primary prevention of early-onset CKD and promote CKD health education among the public. Moreover, the age-specific survival rate of early-onset CKD patients has improved, likely attributable to global advancements in medical technology and healthcare, including early diagnosis, effective treatments (such as medication, dialysis, and kidney transplantation), and enhanced patient management and care (12). However, despite the declining death rate, the age-specific DALY rate has remained stable. This phenomenon reflects that early-onset CKD continues to impose a significant long-term health burden, leading to persistent functional loss and decreased quality of life. Therefore, the stable DALY rate indicates a shift in the burden of disease, underscoring the need for ongoing global focus on prevention strategies and long-term management of early-onset CKD.

As indicated by SDI, health outcomes and disease burdens have previously exhibited correlations with socioeconomic development (13). Our findings mirrored this association, showing persistent disparities in early-onset CKD burden across SDI categories. An inverse V-shaped relationship between SDI and age-specific incidence rate, and a descending trend between SDI and age-specific death and DALY rates, align with prior CKD burden distribution patterns (14). The highest age-specific death and DALY rates were found in countries with low and low-middle SDI, primarily attributed to their limited CKD care quality and drug/surgery access (15). An universal health coverage research showed that lower SDI countries have shifted from communicable to non-communicable diseases and injuries, outpacing health system advancements (16). Hence, lower SDI countries should promptly draw upon experiences from developed nations to formulate policies and invest in public welfare for secondary prevention and healthcare for non-communicable diseases, thereby reducing the death and DALY rates of early-onset CKD. In countries with middle SDI, the age-specific incidence rate of early-onset CKD is highest and increasing the fastest, possibly due to increased and exacerbated CKD risk factors attributable to rapid social and economic changes. Research showed that these middle SDI countries have experienced faster socioeconomic development since 2000 (17), but poor lifestyle and medical intervention contribute significantly to the increase in hypertension and diabetes burden, major risk factors for early-onset CKD (18). Additionally, advancements in CKD screening technology and improvements in registry systems may also contribute to the increased incidence of early-onset CKD in these countries (19). Conversely, in countries with high-middle SDI, the death and DALY rates of early-onset CKD are decreasing more rapidly. This may be attributed to more stable social development, rapid improvements in healthcare resources, and increased public awareness of diseases. A study linking access to and quality of healthcare services with overall development supports this view (20). Therefore, middle SDI countries in the period of rapid transition should draw on the experiences of high SDI countries, focusing more on primary prevention (21).

The global burden of early-onset CKD also varied by gender and age. Our study showed that the age-specific incidence of early-onset CKD was higher in women. This sex-based disparity, predominantly observed in individuals aged 35–39, may be attributed to conditions like acute pyelonephritis and pregnancy-related nephropathy in women of child-bearing age, linked to CKD (22, 23). Additionally, the incidence rate among women is more pronounced in low SDI countries, likely due to limited healthcare access and poor metabolic health (24). Research also showed that inadequate lifestyle control during reproductive years may lead to pregnancy-related hypertension, hyperglycemia, and hyperlipidemia, increasing CKD prevalence (25). Thus, effective early-onset CKD prevention in women is crucial. However, men had higher age-specific death and DALY rates. This might result from a higher incidence of congenital kidney and urinary tract anomalies (26, 27). Additionally, research shows that younger individuals may be more susceptible to congenital or genetic factors. In our study, men aged 15-19 exhibited considerably higher incidence, death, and DALY rates than women in low-SDI regions, suggesting that greater attention should be given to the burden of early-onset CKD in younger males in these areas. Relevant policy departments should adjust early-onset CKD prevention and intervention measures based on gender, age, and region, especially among vulnerable populations. For example, targeted programs addressing pregnancy-related CKD risks for women and specialized education and prevention programs for adolescents should be implemented.

Regarding primary prevention, our study showed that high SBP, high FPG, and high BMI were the three major risk factors leading to early-onset CKD. As reported, hypertension is highly prevalent and is the primary risk factor for CKD (28). Also, strong evidence has indicated that high SBP increases the risk of CKD (29, 30). Similar results were found in our analysis that high SBP was the main contributor to the burden of early-onset CKD and increased globally from 1990. In addition, researchers found that if the prevalence of high SBP was reduced relatively by 25% by 2030, the deaths from CKD would be reduced by 0.8 thousand for adults (29). Moreover, the global prevalence of high SBP was generally higher for men than women (29, 30). Our analysis supported this finding, with a high SBP contributing more to the burden of early-onset CKD in men than in women. In addition, the proportional DALY for high SBP had significantly increased from 1990 to 2019, further indicating the importance of preventing hypertension, especially in men.

Meanwhile, in our analysis, high FPG was the second main contributor to the burden of early-onset CKD in 2019, with a higher attributable burden in men, consistent with the KDIGO clinical practice guideline (31). Moreover, studies have shown that with the occurrence of obesity in younger ages, the prevalence of diabetes is likely to continue to increase substantially (32, 33). This is supported by global data showing a significant increase in the incidence of type 2 diabetes among adolescents and young adults (15–39 years of age) worldwide between 1990 and 2019 (34). A study from China also showed that all-age prevalence of diabetes rose from 3.7% to 6.6%, and death rates for all-age diabetes and diabetes-related CKD increased by 63.5% and 33.3%, respectively, from 1990 to 2016 (35). In addition, our analysis showed that high BMI was the third main contributor to the burden of early-onset CKD, with a higher attributable burden in women. This result aligned with the opinion of a review that obesity is at the epicenter of the global CKD problem (36). Between 1990 and 2017, the prevalence of overweight and obesity has increased globally in all age groups and regions (37). Moreover, the global deaths and DALYs attributable to high BMI were estimated to have more than doubled (38). Therefore, men should focus on stricter blood pressure and blood sugar control, while women should prioritize weight loss, considering different risk factors.

Similarly, across all age groups included in our study, high SBP and high FPG were the leading causes of early-onset CKD DALYs in 2019, with high BMI also contributing significantly to DALYs in the 35-39 age group. These findings further emphasize the importance of managing these three leading risk factors and developing gender- and age-specific strategies for CKD primary prevention. Across all SDI regions, the main attributable risk factors for early-onset CKD maintain consistent contribution ranks, highlighting the necessity of cohesive global policies. High SBP, high FPG, and elevated BMI remain significant factors in all SDI categories. The experience of low- and middle-income countries indicates that a large number of asymptomatic and undiagnosed individuals contribute to the burden of various non-communicable diseases, including diabetes, hypertension, and advanced CKD (39). Nonetheless, studies have demonstrated that early asymptomatic CKD can be identified through simple tests. Identifying and treating high-risk individuals with CKD through case finding can provide benefits at a reasonable cost (11). Therefore, it is necessary to reduce the underreporting of early-onset CKD by expanding screening coverage, improving data collection and reporting systems, standardizing diagnostic criteria, strengthening monitoring, and increasing risk factor screening programs (40). Training healthcare professionals and conducting public health education campaigns may also enhance awareness and improve early detection. Additionally, strengthening global health management and emphasizing proactive health concepts are crucial for addressing these consistent chronic disease risk factors.

## Study limitations

Our study is subject to several limitations. First, estimates of the burden of early-onset CKD largely depend on the availability and quality of data from the GBD 2019. Some countries, especially low-income countries, may lack original/raw data, which could hinder GBD researchers’ ability to make accurate estimates. Second, differences in diagnostic and detection protocols for early-onset CKD across countries and over time may affect the comparability of the results. Given the uncertainty of the original data, caution should be exercised when interpreting the trends in the burden of early-onset CKD found in this study. Third, a narrow focus on significance testing may overlook the clinical relevance of these findings. To mitigate this limitation, we advocate for the development and implementation of multiple analytical methods to broaden and validate the results of this study.

## Conclusions

Although the age-specific death rate of early-onset CKD significantly decreased from 1990 to 2019, the age-specific incidence rate continued to rise and the age-specific DALY rate remained stable. The burdens of early-onset CKD varied by gender, age, and region. Additionally, high SBP, high FPG, and high BMI were the primary risk factors contributing to the burden of early-onset CKD. Targeted primary and secondary prevention measures, as well as healthcare interventions, should be developed based on these risk factors, considering different ages, genders, and regions.

## Data Availability

Publicly available datasets were analyzed in this study. This data can be found here: Global Health Data Exchange GBD results tool (http://ghdx.healthdata.org/gbd-results-tool).
