# Supplementary material for: Global burden of chronic kidney disease in adolescents and young adults, 1990–2019: a systematic analysis for the Global Burden of Disease Study 2019
Source: Front Endocrinol (Lausanne). 2024 Sep 18;15:1389342. doi: 10.3389/fendo.2024.1389342 (PMC11445070; doi:10.3389/fendo.2024.1389342)
Supplement: Supplementary file 1 [file DataSheet1.docx]

***Supplementary Materials***

**Global burden of** **chronic kidney disease** **in** **adolescents and young adults, 1990–2019: a systematic analysis for the Global Burden of Disease Study 2019**

**Ping Sun^1,2^****^†^, Xingyu Ming^3†^, Tiange Song^4†^, Yan Chen^5^, Xin Yang^6^, Zhaochen Sun^5^, Xiaoxia Zheng^1^, Luyao Tong^1^, Zhiwei Ma^7*^, Zhengwei Wan^1*^**

^1^Department of Health Management Center & Institute of Health Management, Sichuan Provincial People's Hospital, University of Electronic Science and Technology of China, Chengdu, China.

^2^School of Nutritional Sciences and Wellness, University of Arizona, Tucson, Arizona, USA.

^3^Department of Medical Records and Statistics, Sichuan Provincial People's Hospital, University of Electronic Science and Technology of China, Chengdu, China.

^4^Department of Laboratory Medicine, Sichuan Provincial People's Hospital, University of Electronic Science and Technology of China, Chengdu, China.

^5^School of Public Health, Southwest Medical University, Luzhou, China.

^6^School of Life Science and Technology, University of Electronic Science and Technology of China, Chengdu, China.

^7^Department of Urology, Sichuan Academy of Medical Sciences and Sichuan Provincial People’s Hospital, Chengdu, China.

*** Correspondence:**Zhengwei Wan
[18715799366@163.com](mailto:18715799366@163.com)

Zhiwei Ma
[704374245@qq.com](mailto:704374245@qq.com)

†These authors have contributed equally to this work

**Table of Content**

Appendix 1. GBD data collection, modeling/analysis, and dissemination 4

Supplementary Table 1. Early onset CKD burden of the 204 countries, including incidence case number and rates in 1990 and 2019, and the estimated annual percentage changes (EAPCs, %) of incidence rate from 1990 to 2019. 4

Supplementary Table 2. Early onset CKD burden of the 204 countries, including death case number and rates in 1990 and 2019, and the estimated annual percentage changes (EAPCs, %) of death rate from 1990 to 2019. 14

Supplementary Table 3. Early onset CKD burden of the 204 countries, including DALY case number and rates in 1990 and 2019, and the estimated annual percentage changes (EAPCs, %) of incidence rate from 1990 to 2019. 23

Supplementary Table 4. Case number of incidence, death, and DALY in 1990 and 2019, and their percentage change (%), by global, 5 SDI regions, and 21 geographical regions. 39

Supplementary Table 5. Proportion (%) of 7 attributable risk factors of early-onset chronic kidney disease (CKD) disability-adjusted life year (DALY) in 2019 for both sex, men, and women (A) including 7 factors, and excluding (B) high fasting plasma glucose and high systolic blood pressure. 42

Supplementary Figure 1. Trends of age-specific (A) incidence rate, (B) death rate, (C) DALY rate of early-onset CKD according to five age-groups, by Global and SDI regions. DALY, disability-adjusted life year; CKD, chronic kidney disease. 44

Supplementary Figure 2. The difference in incidence rate between men and women by age groups and SDI regions, from 1990 to 2019. The difference was calculated using the age-specific rate in males minus that in females, with a difference higher than 0 meaning men have higher rates. DALY, disability-adjusted life year. 45

Supplementary Figure 3. The difference in death rate between men and women by age groups and SDI regions, from 1990 to 2019. The difference was calculated using the age-specific rate in males minus that in females, with a difference higher than 0 meaning men have higher rates. DALY, disability-adjusted life year. 46

Supplementary Figure 4. The difference in DALY rate between men and women by age groups and SDI regions, from 1990 to 2019. The difference was calculated using the age-specific rate in males minus that in females, with a difference higher than 0 meaning men have higher rates. DALY, disability-adjusted life year. 47

Supplementary Figure 5. Temporal trend of age-specific (A) incidence, (B) death, and (C) DALY rates (both sex) for the early onset CKD by global, SDI regions, and age-groups, respectively, from 1990 to 2019. DALY, disability-adjusted life year; CKD, chronic kidney disease. 48

Supplementary Figure 6. Temporal trend of age-specific (A) incidence, (B) death, and (C) DALY rates (female and male) for the early onset CKD by global, SDI regions, and age-groups, respectively, from 1990 to 2019. EAPC, estimated annual percentage change;DALY, disability-adjusted life year; CKD, chronic kidney disease. 49

Supplementary Figure 7. EAPCs of age-specific (A) incidence, (B) death, and (C) DALY rates (Both sex, female, and male) for the early onset CKD by global, SDI regions, and age-groups, respectively, from 1990 to 2019. EAPC, estimated annual percentage change; DALY, disability-adjusted life year; CKD, chronic kidney disease. 50

Supplementary Figure 8. The relationship between age-specific (A) incidence rate, (B) death rate, and (C) DALY rate of early-onset CKD and SDI (1990–2019) across 21 geographical regions. For each region, points from left to right depict estimates from each year from 1990 to 2019. Solid blue line and stark area shows expected values across the spectrum of the SDI. DALY, disability-adjusted life year; CKD, chronic kidney disease; SDI, socio-demographic index. 51

Supplementary Figure 9. Proportion of early-onset CKD DALYs attributable to 7 risk factors classified by sex, in the five age-groups, globally in 2019. DALY, disability-adjusted life year; CKD, chronic kidney disease. 52

Supplementary Figure 10. Proportion of attributable risk factors of early-onset CKD DALY in 1990 and 2019 for men and women (A) including 7 factors, and excluding (B) high fasting plasma glucose and high systolic blood pressure. DALY, disability-adjusted life year; CKD, chronic kidney disease. 53

Appendix 1. GBD data collection, modeling/analysis, and dissemination

The Global Burden of Disease (GBD) study is a comprehensive research initiative aimed at systematically assessing the health status and disease burden of populations worldwide. An international network comprising over 11,500 collaborators from 164 countries and territories contributed to the generation of GBD metrics through data provision, review, and analysis. GBD data collection involves diverse sources, including epidemiological surveys, hospital records, vital registration systems, disease surveillance systems, and additional sources such as academic papers and policy reports (https://ghdx.healthdata.org/gbd-2019/data-input-sources). The data is standardized using the International Classification of Diseases (ICD) codes to ensure accuracy and comparability (https://ghdx.healthdata.org/record/ihme-data/gbd-2019-cause-icd-code-mappings). Sophisticated modeling tools, such as DisMod-MR and Spatiotemporal Gaussian Process Regression (ST-GPR), are employed to estimate prevalence, incidence, and mortality rates. Data processing includes corrections for heterogeneity and biases, as well as uncertainty analysis through Monte Carlo simulations. Key health metrics used are Disability-Adjusted Life Years (DALYs), Years of Life Lost (YLLs), and Years Lived with Disability (YLDs) (https://www.healthdata.org/research-analysis/about-gbd/gbd-data-and-tools-guide). Dissemination of GBD findings is achieved through interactive tools like GBD Compare and Viz Hub (https://ghdx.healthdata.org/gbd-2019/sources). These tools facilitate the exploration and comparison of health data across regions and time periods. The primary goal of GBD findings is to provide a comprehensive framework for understanding global and local health trends, thereby supporting evidence-based health decision-making and resource allocation.

Supplementary Table 1. Early onset CKD burden of the 204 countries, including incidence case number and rates in 1990 and 2019, and the estimated annual percentage changes (EAPCs, %) of incidence rate from 1990 to 2019.

| **Location name** | **Case 1990 (x 1000)** | **Case 2019 (x 1000)** | **Rate 1990 (per 100k)** | **Rate 2019 (per 100k)** | **EAPC 1990-2019 (%)** |
| --- | --- | --- | --- | --- | --- |
| China | 103.37 (72.87, 133.34) | 104.15 (69.3, 140.9) | 18.81 (13.26, 24.27) | 20.93 (13.92, 28.31) | 0.61 (0.48, 0.74) |
| Mali | 1.16 (0.73, 1.73) | 2.15 (1.55, 2.74) | 38.96 (24.63, 57.96) | 30.17 (21.86, 38.47) | 0.35 (0.16, 0.55) |
| Maldives | 0.02 (0.01, 0.03) | 1.29 (0.95, 1.66) | 24.79 (17.64, 32.28) | 41.1 (30.25, 52.74) | 0.99 (0.91, 1.06) |
| Mozambique | 0.57 (0.41, 0.73) | 4.55 (3.33, 5.88) | 12.5 (9.05, 16.03) | 33.05 (24.18, 42.63) | 1.25 (1.17, 1.33) |
| Malta | 0.02 (0.01, 0.02) | 3.12 (2.24, 4.04) | 11.19 (6.82, 16.45) | 31.09 (22.31, 40.2) | 0.81 (0.69, 0.93) |
| Samoa | 0.03 (0.02, 0.04) | 3.16 (2.29, 4.03) | 44.12 (33.74, 54.86) | 39.34 (28.48, 50.13) | 1.06 (0.89, 1.24) |
| Turkmenistan | 0.79 (0.62, 0.97) | 11.35 (8.28, 14.46) | 51.3 (40.23, 63.27) | 46.77 (34.14, 59.57) | 1.56 (1.45, 1.67) |
| Solomon Islands | 0.11 (0.09, 0.13) | 0.15 (0.11, 0.19) | 84.76 (69.48, 99.77) | 27.66 (19.9, 35.84) | -0.29 (-0.37, -0.22) |
| Tajikistan | 0.7 (0.53, 0.92) | 0.21 (0.16, 0.25) | 33.28 (24.89, 43.28) | 78.88 (62.59, 95.53) | 1.49 (1.24, 1.74) |
| Romania | 2.46 (1.78, 3.23) | 0.02 (0.02, 0.03) | 28.26 (20.47, 37.15) | 54.12 (40.63, 67.84) | 1.16 (1.05, 1.26) |
| Myanmar | 7.06 (5.4, 8.91) | 0.06 (0.05, 0.08) | 41.7 (31.89, 52.6) | 51.8 (40.54, 63.72) | 0.45 (0.38, 0.53) |
| Mauritania | 0.25 (0.19, 0.31) | 2.77 (2.06, 3.53) | 32.55 (24.2, 40.75) | 34.9 (25.94, 44.51) | 0.35 (0.30, 0.40) |
| Netherlands | 0.52 (0.29, 0.78) | 0.04 (0.03, 0.05) | 8.62 (4.81, 13) | 78.19 (62.06, 95.95) | 0.43 (0.38, 0.47) |
| India | 116.17 (88.43, 144.15) | 0.57 (0.42, 0.73) | 34.09 (25.95, 42.3) | 51 (37.87, 65.26) | -0.02 (-0.13, 0.10) |
| Serbia | 0.88 (0.65, 1.13) | 1.57 (1.26, 1.92) | 25.72 (18.95, 32.9) | 76.99 (61.89, 94.2) | 1.28 (1.16, 1.40) |
| Uzbekistan | 5.25 (4.04, 6.43) | 11.58 (8.33, 15.28) | 61.12 (47.05, 74.8) | 29.35 (21.11, 38.74) | 1.02 (0.90, 1.15) |
| Tonga | 0.01 (0.01, 0.02) | 0.2 (0.15, 0.25) | 38.77 (29.19, 48.64) | 55.45 (41.19, 68.81) | 1.19 (1.11, 1.27) |
| Equatorial Guinea | 0.02 (0.02, 0.03) | 0.46 (0.33, 0.62) | 14.29 (10.25, 18.48) | 35.99 (25.73, 47.78) | 1.46 (1.28, 1.63) |
| American Samoa | 0.01 (0.01, 0.01) | 0.72 (0.55, 0.89) | 48.49 (36.58, 60.18) | 60.47 (46.45, 75.27) | 1.14 (0.96, 1.32) |
| Niue | 0 (0, 0) | 0.02 (0.01, 0.02) | 45.99 (34.5, 57.59) | 70.57 (54.86, 86.33) | 1.39 (1.28, 1.49) |
| Rwanda | 0.46 (0.33, 0.59) | 11.24 (8.62, 14.07) | 16.69 (11.97, 21.75) | 78.78 (60.46, 98.63) | -0.05 (-0.22, 0.12) |
| USA | 21.56 (14.06, 30.32) | 0.4 (0.3, 0.5) | 21.15 (13.79, 29.75) | 41.25 (31.06, 51.84) | -0.74 (-0.92, -0.56) |
| Morocco | 2.19 (1.53, 2.87) | 23.28 (17.76, 28.8) | 21.07 (14.7, 27.56) | 50.47 (38.5, 62.43) | 2.71 (2.66, 2.77) |
| Seychelles | 0.01 (0.01, 0.01) | 1.08 (0.75, 1.44) | 31.38 (23.19, 40.5) | 35.4 (24.73, 47.14) | 1.47 (1.30, 1.64) |
| Gabon | 0.08 (0.05, 0.1) | 4.33 (3.27, 5.53) | 19.74 (14.12, 25.58) | 62.37 (47.06, 79.61) | 1.30 (1.23, 1.36) |
| Northern Mariana Islands | 0.02 (0.01, 0.02) | 2.48 (1.87, 3.12) | 67.3 (49.54, 85.7) | 58.04 (43.84, 73.15) | -0.24 (-0.87, 0.39) |
| Grenada | 0.01 (0.01, 0.02) | 1.02 (0.73, 1.38) | 41.58 (31.06, 52.07) | 35.78 (25.6, 48.28) | 1.39 (1.26, 1.53) |
| Vanuatu | 0.02 (0.02, 0.03) | 0.04 (0.03, 0.04) | 38.82 (29.85, 47.65) | 82.98 (65.03, 102.03) | 0.90 (0.83, 0.98) |
| Bangladesh | 5.87 (4.02, 7.71) | 2.72 (1.81, 3.66) | 13.75 (9.43, 18.07) | 15.68 (10.42, 21.14) | 1.88 (1.60, 2.17) |
| El Salvador | 0.81 (0.6, 1.02) | 1.91 (1.47, 2.36) | 38.59 (28.99, 48.73) | 71.56 (55.33, 88.49) | 2.53 (2.44, 2.62) |
| Brunei | 0.03 (0.02, 0.05) | 0.53 (0.34, 0.73) | 27 (18.56, 36.53) | 24.85 (15.98, 34.22) | -0.05 (-0.29, 0.20) |
| Cambodia | 0.92 (0.69, 1.15) | 0.38 (0.29, 0.5) | 23.74 (17.67, 29.72) | 36.98 (27.84, 48.27) | 0.40 (0.21, 0.59) |
| Niger | 0.69 (0.52, 0.87) | 0.21 (0.15, 0.27) | 24.88 (18.61, 31.26) | 51.9 (37.71, 67.86) | 0.75 (0.68, 0.81) |
| Slovakia | 0.57 (0.4, 0.75) | 1.21 (0.76, 1.72) | 27.75 (19.44, 36.5) | 14.58 (9.11, 20.67) | 0.63 (0.55, 0.71) |
| Liberia | 0.18 (0.13, 0.24) | 2.08 (1.53, 2.71) | 27.63 (20.06, 35.87) | 37.04 (27.17, 48.25) | 1.18 (1.01, 1.35) |
| Palestine | 0.22 (0.16, 0.29) | 0.28 (0.2, 0.36) | 29.18 (20.96, 37.61) | 49.16 (35.93, 64.37) | 0.42 (0.37, 0.47) |
| Zimbabwe | 1.13 (0.83, 1.44) | 0.36 (0.26, 0.47) | 28.46 (21.01, 36.35) | 42.81 (31.13, 56.16) | 0.37 (0.27, 0.47) |
| Jamaica | 0.34 (0.25, 0.43) | 8.32 (5.62, 11.28) | 34.21 (25.41, 43.68) | 25.31 (17.11, 34.32) | 0.98 (0.85, 1.10) |
| Bhutan | 0.08 (0.06, 0.1) | 3.32 (2.05, 4.9) | 29.51 (22.56, 37.22) | 13.03 (8.04, 19.23) | 0.85 (0.77, 0.93) |
| Albania | 0.38 (0.28, 0.5) | 1.45 (1.03, 1.92) | 27.13 (19.82, 35.01) | 47.52 (33.67, 62.91) | 0.74 (0.40, 1.09) |
| Estonia | 0.2 (0.14, 0.26) | 0.99 (0.7, 1.3) | 34.85 (25.2, 46.43) | 35.17 (24.96, 45.93) | 1.54 (1.27, 1.80) |
| Suriname | 0.05 (0.03, 0.09) | 0.31 (0.18, 0.45) | 33.38 (19.89, 52.8) | 9.72 (5.69, 14.12) | 1.47 (1.39, 1.55) |
| Burkina Faso | 0.7 (0.5, 0.87) | 0.37 (0.23, 0.54) | 21.8 (15.77, 27.3) | 13.36 (8.16, 19.53) | 1.48 (1.41, 1.55) |
| Guatemala | 0.99 (0.75, 1.27) | 0.42 (0.26, 0.61) | 34.06 (25.75, 43.81) | 14.6 (8.98, 21.01) | 1.34 (1.29, 1.40) |
| Bahamas | 0.04 (0.03, 0.06) | 3.37 (2.31, 4.48) | 37.06 (27.07, 47.22) | 19.36 (13.24, 25.7) | 1.58 (1.29, 1.87) |
| Pakistan | 13.79 (10.56, 17.1) | 1.31 (0.86, 1.82) | 33.68 (25.78, 41.76) | 19.31 (12.69, 26.68) | 2.03 (1.88, 2.18) |
| Uruguay | 0.14 (0.09, 0.18) | 1.18 (0.9, 1.5) | 11.96 (7.76, 16.27) | 59.72 (45.66, 75.97) | 1.76 (1.59, 1.94) |
| Trinidad | 0.17 (0.13, 0.22) | 0.19 (0.13, 0.26) | 34.75 (25.98, 44.21) | 15.96 (10.38, 21.71) | 1.37 (1.26, 1.48) |
| Guyana | 0.12 (0.09, 0.15) | 0.09 (0.06, 0.11) | 35.84 (26.3, 45.12) | 57.8 (43.28, 73.04) | 1.06 (0.97, 1.14) |
| Comoros | 0.03 (0.02, 0.04) | 33.79 (24.4, 44.9) | 17.98 (13.08, 22.64) | 70.37 (50.81, 93.5) | 1.51 (1.45, 1.56) |
| Oman | 0.15 (0.1, 0.21) | 0 (0, 0) | 18.92 (12.78, 26.21) | 11.88 (6.86, 17.3) | 0.56 (0.51, 0.62) |
| Burundi | 0.33 (0.22, 0.42) | 0.67 (0.48, 0.89) | 15.74 (10.84, 20.3) | 37.68 (26.63, 49.98) | 1.29 (1.21, 1.36) |
| Benin | 0.48 (0.36, 0.6) | 0.35 (0.22, 0.51) | 28.04 (20.91, 35.01) | 12.5 (7.84, 18.12) | 1.30 (1.04, 1.56) |
| Barbados | 0.04 (0.03, 0.05) | 1.41 (0.88, 2.05) | 34.68 (25.44, 44.37) | 12.3 (7.69, 17.87) | 1.16 (1.13, 1.19) |
| Mexico | 13.8 (10.11, 17.66) | 0.28 (0.18, 0.38) | 38.73 (28.37, 49.54) | 19.61 (13.06, 27.05) | 1.08 (1.05, 1.11) |
| Djibouti | 0.03 (0.02, 0.04) | 0.05 (0.04, 0.07) | 14.47 (10.38, 18.44) | 52.98 (38.75, 67.65) | 1.74 (1.65, 1.83) |
| Cameroon | 1.5 (1.12, 1.89) | 0.02 (0.01, 0.02) | 39.53 (29.6, 49.79) | 54.96 (39.78, 70.07) | 1.32 (1.13, 1.52) |
| Austria | 0.31 (0.18, 0.45) | 0.29 (0.18, 0.4) | 10.27 (6.11, 14.91) | 17.79 (11.4, 24.67) | 0.45 (0.35, 0.55) |
| Greenland | 0 (0, 0) | 1.3 (0.92, 1.73) | 8.87 (5.11, 13) | 26.96 (18.99, 35.79) | 2.36 (2.09, 2.63) |
| Jordan | 0.39 (0.28, 0.5) | 0.01 (0.01, 0.02) | 25.34 (18.13, 32.15) | 10.14 (5.88, 14.75) | 1.52 (1.03, 2.02) |
| Honduras | 0.58 (0.42, 0.74) | 0.03 (0.02, 0.04) | 33.4 (24.48, 42.78) | 12.93 (8.15, 18.49) | -0.34 (-0.41, -0.26) |
| Colombia | 3.89 (2.82, 5.16) | 6.53 (4.67, 8.66) | 27.68 (20.01, 36.7) | 45.2 (32.36, 59.98) | 0.83 (0.77, 0.90) |
| Syria | 1.45 (1.01, 1.89) | 0.65 (0.47, 0.82) | 30.11 (21.06, 39.25) | 54.39 (39.77, 68.75) | -0.43 (-0.62, -0.25) |
| Guam | 0.02 (0.02, 0.03) | 2.93 (2.1, 3.77) | 38.83 (28.89, 49.72) | 40.66 (29.12, 52.3) | 1.27 (1.07, 1.47) |
| Haiti | 0.8 (0.6, 1) | 0.36 (0.25, 0.48) | 32.75 (24.85, 41.27) | 57.76 (40.1, 77.69) | 2.16 (2.06, 2.26) |
| United Arab Emirates | 0.37 (0.25, 0.49) | 2.01 (1.48, 2.55) | 38.62 (25.74, 51.01) | 44.74 (32.9, 56.62) | 2.03 (1.89, 2.17) |
| Nicaragua | 0.75 (0.57, 0.94) | 18.29 (13.15, 23.57) | 50.9 (38.51, 64.03) | 44.58 (32.05, 57.47) | 1.22 (1.05, 1.38) |
| Finland | 0.15 (0.09, 0.22) | 0.47 (0.3, 0.65) | 8.44 (5.12, 12.36) | 14.42 (9.39, 19.98) | 0.90 (0.83, 0.96) |
| Palau | 0 (0, 0.01) | 9.11 (6.51, 11.91) | 60.93 (44.05, 78.82) | 48.81 (34.84, 63.8) | 0.41 (0.24, 0.58) |
| Latvia | 0.3 (0.22, 0.4) | 36.34 (27.89, 44.78) | 31.7 (23.2, 42.28) | 72.41 (55.59, 89.24) | 1.77 (1.67, 1.88) |
| South Korea | 2.25 (1.64, 2.89) | 2.99 (1.89, 4.3) | 27.27 (19.85, 35.07) | 13.69 (8.66, 19.64) | 0.26 (0.01, 0.51) |
| Croatia | 0.46 (0.31, 0.61) | 6.93 (5.02, 9.11) | 25.29 (17.11, 33.54) | 36.24 (26.24, 47.64) | 1.36 (1.15, 1.58) |
| Iran | 6.62 (4.57, 8.7) | 0.18 (0.14, 0.22) | 29.32 (20.23, 38.53) | 55.48 (42.14, 68.83) | 1.38 (1.17, 1.58) |
| Puerto Rico | 0.48 (0.36, 0.61) | 0.17 (0.1, 0.25) | 34.09 (25.49, 43.34) | 9.6 (5.63, 13.96) | 1.86 (1.80, 1.91) |
| Kuwait | 0.3 (0.21, 0.39) | 0.02 (0.01, 0.03) | 34.21 (24.11, 44.8) | 14.26 (8.7, 20.48) | -0.15 (-0.35, 0.05) |
| Poland | 4.32 (2.98, 5.82) | 5.65 (4.22, 7.07) | 29.86 (20.64, 40.22) | 53.17 (39.68, 66.45) | 2.24 (2.15, 2.33) |
| Sudan | 1.58 (1.11, 2.04) | 0.82 (0.56, 1.11) | 20.76 (14.58, 26.8) | 47.64 (32.7, 64.78) | 2.17 (2.09, 2.25) |
| Costa Rica | 0.51 (0.37, 0.68) | 14.78 (10.67, 18.91) | 40.1 (28.84, 52.6) | 80.69 (58.24, 103.2) | 1.12 (1.08, 1.15) |
| Mongolia | 0.53 (0.41, 0.67) | 2.29 (1.66, 2.93) | 60.46 (46.31, 75.81) | 41.62 (30.12, 53.15) | 0.65 (0.51, 0.80) |
| Venezuela | 2.81 (2.06, 3.63) | 3.76 (2.66, 4.97) | 35.03 (25.69, 45.3) | 27.59 (19.5, 36.48) | 1.06 (1.01, 1.12) |
| Laos | 0.55 (0.41, 0.69) | 6.38 (4.78, 7.98) | 35.4 (26.74, 44.59) | 48.81 (36.54, 61.05) | 1.56 (1.38, 1.74) |
| Moldova | 0.43 (0.31, 0.56) | 4.03 (2.74, 5.47) | 24.73 (17.51, 32.29) | 31.9 (21.68, 43.24) | 0.81 (0.76, 0.86) |
| Lithuania | 0.46 (0.33, 0.6) | 0.54 (0.39, 0.7) | 32.74 (23.9, 43.42) | 25.64 (18.33, 33.24) | 0.61 (0.51, 0.72) |
| Saudi Arabia | 2.19 (1.59, 2.81) | 1.18 (0.84, 1.54) | 32.63 (23.78, 41.95) | 39.83 (28.19, 51.66) | 1.16 (0.89, 1.42) |
| Portugal | 0.42 (0.26, 0.59) | 4.28 (3.2, 5.48) | 11.14 (6.93, 15.71) | 54 (40.37, 69.14) | 0.87 (0.82, 0.92) |
| Japan | 9.94 (6.9, 13.39) | 0.2 (0.14, 0.27) | 22.16 (15.37, 29.86) | 34.07 (24.28, 45.72) | 2.37 (1.91, 2.82) |
| Bolivia | 0.49 (0.36, 0.64) | 2.3 (1.68, 2.95) | 20 (14.55, 25.79) | 43.21 (31.55, 55.42) | 1.36 (1.22, 1.50) |
| Canada | 1.22 (0.72, 1.79) | 1.1 (0.82, 1.4) | 11.01 (6.5, 16.14) | 57.54 (42.56, 73.16) | 0.30 (0.23, 0.38) |
| Cape Verde | 0.03 (0.02, 0.04) | 0.52 (0.3, 0.75) | 22.74 (16.25, 28.8) | 9.83 (5.7, 14.21) | 1.53 (1.45, 1.61) |
| Republic of Congo | 0.2 (0.15, 0.26) | 1.95 (1.38, 2.55) | 21.58 (15.94, 27.51) | 44.04 (31.24, 57.48) | 0.35 (0.17, 0.52) |
| Ethiopia | 2.92 (2.09, 3.74) | 0.43 (0.26, 0.63) | 15.65 (11.22, 20.04) | 12.34 (7.29, 17.83) | 1.07 (0.62, 1.51) |
| Qatar | 0.06 (0.04, 0.08) | 0.03 (0.02, 0.03) | 25.17 (16.29, 34.35) | 65.45 (49.17, 81.48) | 0.29 (0.12, 0.47) |
| UK | 2.31 (1.41, 3.38) | 8.53 (6.11, 11.11) | 11.06 (6.74, 16.21) | 49.74 (35.61, 64.75) | 1.52 (1.24, 1.80) |
| Gambia | 0.1 (0.07, 0.12) | 23.59 (17.58, 29.78) | 25.71 (19.29, 31.77) | 28.14 (20.96, 35.51) | 0.69 (0.59, 0.78) |
| Czech Republic | 0.94 (0.64, 1.25) | 0.04 (0.03, 0.05) | 25.17 (17.31, 33.52) | 61.6 (46.8, 77.42) | 1.17 (1.04, 1.31) |
| Nauru | 0 (0, 0) | 6.06 (4.21, 7.92) | 60.16 (46.08, 75.61) | 17.31 (12.03, 22.64) | 1.29 (1.22, 1.37) |
| Belgium | 0.41 (0.25, 0.6) | 0.06 (0.04, 0.07) | 11.02 (6.62, 16.07) | 19.37 (13.51, 25.31) | 1.92 (1.76, 2.07) |
| Ecuador | 0.98 (0.69, 1.3) | 1.6 (1.17, 2.08) | 23.87 (16.81, 31.65) | 21.14 (15.4, 27.38) | 1.38 (1.17, 1.59) |
| Yemen | 0.95 (0.65, 1.25) | 6.57 (4.7, 8.52) | 20.6 (14.18, 27.02) | 44.95 (32.13, 58.29) | 1.61 (1.50, 1.73) |
| Kiribati | 0.02 (0.02, 0.02) | 1.75 (1.34, 2.23) | 64.72 (50.6, 78.74) | 30.31 (23.08, 38.55) | 0.83 (0.70, 0.95) |
| Belize | 0.02 (0.02, 0.03) | 2.09 (1.47, 2.7) | 32.6 (23.59, 42.09) | 18.64 (13.12, 24.1) | 1.04 (1.00, 1.09) |
| Dominican Republic | 0.71 (0.52, 0.92) | 1.13 (0.83, 1.41) | 22.98 (16.77, 29.94) | 32.22 (23.63, 40.46) | 0.48 (0.44, 0.53) |
| Malaysia | 1.75 (1.29, 2.22) | 4.4 (3.35, 5.5) | 23.65 (17.37, 29.94) | 40.82 (31.12, 51.12) | 0.30 (0.03, 0.56) |
| Ghana | 1.58 (1.19, 1.95) | 1.16 (0.87, 1.44) | 27.56 (20.74, 33.96) | 36.54 (27.37, 45.04) | 1.62 (1.55, 1.69) |
| Nigeria | 8.32 (6.14, 10.49) | 0.49 (0.37, 0.6) | 24.44 (18.02, 30.8) | 51.21 (39.43, 62.87) | 1.01 (0.93, 1.09) |
| Mauritius | 0.23 (0.17, 0.3) | 1.94 (1.3, 2.58) | 46.82 (34.1, 60.71) | 11.99 (8.06, 15.95) | 0.93 (0.82, 1.04) |
| Bermuda | 0.01 (0.01, 0.01) | 0.32 (0.24, 0.4) | 28.93 (20.12, 38.28) | 34.33 (25.5, 42.9) | 1.38 (1.29, 1.48) |
| Peru | 1.43 (1, 1.89) | 0.51 (0.38, 0.66) | 16.1 (11.27, 21.33) | 48.83 (36.08, 62.73) | 0.46 (0.35, 0.58) |
| Cuba | 1.3 (0.93, 1.68) | 0.34 (0.25, 0.44) | 26.81 (19.14, 34.61) | 33.54 (24.74, 42.97) | 0.77 (0.70, 0.84) |
| Panama | 0.35 (0.26, 0.45) | 5.1 (3.83, 6.29) | 34.88 (25.28, 44.73) | 37.45 (28.16, 46.24) | 0.79 (0.73, 0.86) |
| Eritrea | 0.2 (0.15, 0.26) | 0.35 (0.26, 0.45) | 17.65 (12.71, 22.57) | 74.66 (54.68, 95) | 1.52 (1.23, 1.82) |
| France | 2.28 (1.35, 3.29) | 1.72 (1.32, 2.15) | 10.35 (6.13, 14.96) | 35.71 (27.3, 44.57) | 0.95 (0.74, 1.16) |
| Taiwan | 2.16 (1.57, 2.82) | 0.1 (0.07, 0.13) | 23.36 (16.98, 30.59) | 19.71 (13.68, 26.12) | 3.06 (2.99, 3.13) |
| North Macedonia | 0.23 (0.17, 0.3) | 0.3 (0.23, 0.37) | 28.61 (20.77, 36.86) | 58.61 (45.04, 72.41) | 1.59 (1.38, 1.79) |
| Hungary | 0.87 (0.59, 1.15) | 1.95 (1.4, 2.5) | 23.46 (16.03, 31.2) | 17.42 (12.51, 22.29) | 0.53 (0.27, 0.79) |
| Guinea | 0.61 (0.45, 0.77) | 0.01 (0.01, 0.02) | 28.22 (20.92, 35.86) | 67.87 (52.41, 84.52) | 0.90 (0.77, 1.03) |
| Slovenia | 0.17 (0.11, 0.23) | 2.33 (1.7, 2.98) | 21.79 (14.6, 30.06) | 46.41 (33.85, 59.46) | 0.56 (0.44, 0.68) |
| Kenya | 1.09 (0.75, 1.44) | 6.17 (4.48, 7.91) | 12.48 (8.59, 16.4) | 40.93 (29.72, 52.48) | 1.02 (1.00, 1.04) |
| Lesotho | 0.2 (0.15, 0.25) | 1.97 (1.49, 2.51) | 28.82 (21.33, 36.27) | 46.64 (35.16, 59.5) | 0.42 (0.35, 0.49) |
| Democratic Republic of the Congo | 2.06 (1.46, 2.65) | 0.05 (0.03, 0.06) | 14.43 (10.21, 18.52) | 53.56 (40.28, 66.95) | 1.63 (1.43, 1.83) |
| Tuvalu | 0 (0, 0) | 10.43 (7.66, 13.17) | 42.9 (31.97, 53.16) | 43.54 (31.98, 55.01) | 0.30 (0.10, 0.51) |
| Chad | 0.54 (0.4, 0.67) | 0.58 (0.43, 0.75) | 25.57 (18.91, 32.05) | 20.31 (14.88, 26.03) | 1.53 (1.29, 1.76) |
| Antigua | 0.01 (0.01, 0.01) | 0.07 (0.04, 0.1) | 36.68 (27.08, 46.5) | 14.19 (8.46, 20.63) | 0.85 (0.71, 0.98) |
| Dominica | 0.01 (0.01, 0.02) | 0.02 (0.02, 0.03) | 46.09 (34.61, 57.41) | 56.88 (41.84, 71.44) | -0.37 (-0.48, -0.26) |
| Nepal | 2.03 (1.5, 2.58) | 0.5 (0.37, 0.63) | 27.76 (20.56, 35.3) | 23.63 (17.64, 30.03) | 0.53 (0.40, 0.67) |
| Cyprus | 0.03 (0.02, 0.05) | 6.01 (4.52, 7.46) | 11.34 (6.99, 16.12) | 49.73 (37.37, 61.71) | 2.64 (2.56, 2.72) |
| Namibia | 0.15 (0.12, 0.19) | 0.82 (0.59, 1.05) | 27.2 (20.59, 33.97) | 39.23 (28.29, 50.46) | 2.14 (2.06, 2.22) |
| New Zealand | 0.21 (0.13, 0.29) | 1.42 (1.03, 1.81) | 15.11 (9.73, 21.09) | 18.07 (13.08, 23.03) | 0.55 (0.46, 0.63) |
| Bahrain | 0.06 (0.04, 0.09) | 13.13 (9.47, 17.25) | 24.38 (17.1, 33.55) | 38.59 (27.82, 50.66) | 1.58 (1.43, 1.72) |
| Afghanistan | 1.23 (0.91, 1.55) | 2.43 (1.8, 3.08) | 31.84 (23.63, 39.95) | 28.46 (21.09, 36.03) | 1.05 (1.00, 1.09) |
| Ivory Coast | 1.58 (1.2, 1.96) | 3.13 (2.06, 4.22) | 33.4 (25.3, 41.42) | 14.16 (9.34, 19.11) | 1.46 (1.36, 1.55) |
| North Korea | 4 (2.71, 5.35) | 1.91 (1.4, 2.4) | 18.99 (12.87, 25.4) | 31.13 (22.83, 39.08) | 0.91 (0.84, 0.97) |
| Spain | 1.64 (1.05, 2.33) | 0.22 (0.13, 0.31) | 11.08 (7.08, 15.72) | 12.06 (7.49, 17.12) | 0.27 (0.21, 0.34) |
| Montenegro | 0.09 (0.07, 0.12) | 0 (0, 0) | 37.02 (27.26, 47.15) | 10.64 (6.2, 15.43) | 1.79 (1.71, 1.87) |
| Denmark | 0.21 (0.12, 0.31) | 0 (0, 0) | 11.06 (6.52, 16.24) | 11.02 (6.7, 15.73) | 1.75 (1.47, 2.03) |
| Tunisia | 0.7 (0.48, 0.93) | 1.77 (1.26, 2.27) | 20.36 (14.02, 26.85) | 16.16 (11.56, 20.76) | 2.06 (1.94, 2.18) |
| Madagascar | 0.66 (0.45, 0.85) | 0 (0, 0) | 14.54 (10.05, 18.76) | 57.38 (43.85, 70.11) | 0.61 (0.53, 0.69) |
| South Sudan | 0.31 (0.21, 0.4) | 0.02 (0.01, 0.02) | 13.16 (9.08, 16.97) | 58.28 (43.28, 73.97) | 0.72 (0.59, 0.84) |
| Lebanon | 0.31 (0.22, 0.41) | 1.03 (0.7, 1.41) | 25.77 (18.3, 33.9) | 40.08 (27.43, 54.94) | 1.96 (1.87, 2.05) |
| Belarus | 1.23 (0.88, 1.65) | 0 (0, 0) | 31.15 (22.21, 41.75) | 58.95 (44.61, 73.22) | 0.93 (0.83, 1.03) |
| Zambia | 0.57 (0.42, 0.72) | 0.04 (0.03, 0.04) | 18.8 (13.81, 23.71) | 58.5 (44.55, 73.75) | 2.06 (1.87, 2.25) |
| Sao Tome and Principe | 0.02 (0.01, 0.02) | 1.16 (0.81, 1.57) | 40.49 (30.35, 49.83) | 55.44 (38.78, 75.13) | 1.09 (1.05, 1.13) |
| Sweden | 0.25 (0.14, 0.37) | 0.1 (0.07, 0.12) | 8.44 (4.72, 12.74) | 38.32 (27.74, 48.94) | 1.63 (1.44, 1.81) |
| Andorra | 0 (0, 0) | 188.77 (140.67, 239.5) | 9.97 (5.83, 14.53) | 31.66 (23.6, 40.17) | 0.99 (0.93, 1.04) |
| Malawi | 0.49 (0.32, 0.64) | 16.13 (11.77, 21.07) | 13.48 (8.88, 17.68) | 23.79 (17.35, 31.07) | 1.06 (0.53, 1.58) |
| Marshall Islands | 0.01 (0.01, 0.01) | 0 (0, 0) | 50.07 (38.51, 61.31) | 12.12 (7.38, 17.32) | 1.48 (1.37, 1.60) |
| Turkey | 5.33 (3.85, 6.93) | 1.23 (0.87, 1.57) | 21.56 (15.55, 28.04) | 16.12 (11.45, 20.66) | 1.45 (1.24, 1.66) |
| Swaziland | 0.11 (0.09, 0.14) | 0 (0, 0) | 37.47 (28.76, 46.57) | 78.97 (60.69, 99.38) | 1.90 (1.83, 1.97) |
| Bosnia and Herzegovina | 0.49 (0.35, 0.63) | 0.54 (0.39, 0.69) | 25.31 (18.32, 32.41) | 15.71 (11.29, 20.27) | 1.47 (1.09, 1.85) |
| Indonesia | 21.67 (16.09, 27.12) | 0.02 (0.01, 0.03) | 27.76 (20.62, 34.75) | 74.09 (44.15, 112.34) | 1.67 (1.61, 1.74) |
| Guinea-Bissau | 0.15 (0.11, 0.18) | 32.16 (22.9, 41.31) | 38.74 (29.81, 47.26) | 37.34 (26.59, 47.96) | 0.15 (0.08, 0.23) |
| Papua New Guinea | 0.42 (0.31, 0.53) | 2.07 (1.56, 2.57) | 25.51 (18.83, 31.9) | 80.22 (60.65, 99.66) | 1.25 (1.14, 1.36) |
| Libya | 0.45 (0.32, 0.58) | 0 (0, 0) | 27.23 (19.57, 35.49) | 67.15 (50.43, 84.47) | 1.96 (1.68, 2.24) |
| Georgia | 0.85 (0.64, 1.1) | 3.44 (2.12, 5.05) | 40.11 (30.08, 51.62) | 42.12 (25.98, 61.78) | 1.72 (1.61, 1.82) |
| Ireland | 0.2 (0.12, 0.28) | 0.16 (0.12, 0.2) | 14.59 (9.1, 20.53) | 44.44 (33.06, 55.95) | 0.85 (0.79, 0.90) |
| Saint Lucia | 0.02 (0.02, 0.03) | 0.01 (0.01, 0.01) | 37.38 (28.48, 46.38) | 62.83 (48.33, 78.37) | 1.71 (1.55, 1.87) |
| Israel | 0.23 (0.15, 0.33) | 0.58 (0.42, 0.74) | 12.28 (7.62, 17.44) | 35.88 (26.19, 46.08) | 1.13 (1.07, 1.18) |
| Cook Islands | 0 (0, 0) | 2.32 (1.73, 2.93) | 36.24 (27.29, 45.42) | 28.52 (21.33, 36.1) | 1.89 (1.59, 2.19) |
| Brazil | 18.1 (13.09, 23.17) | 20.17 (14.19, 26.72) | 28.85 (20.86, 36.92) | 56.64 (39.84, 75.04) | 0.53 (0.48, 0.57) |
| Saint Vincent | 0.02 (0.01, 0.02) | 2.8 (2.16, 3.42) | 34.78 (25.79, 44.22) | 99.93 (76.9, 121.75) | 0.64 (0.58, 0.69) |
| Russia | 28.19 (19.96, 36.55) | 0.83 (0.61, 1.03) | 48.44 (34.3, 62.81) | 51.86 (37.98, 64.69) | 0.86 (0.78, 0.94) |
| Ukraine | 5.21 (3.55, 7.14) | 0.99 (0.69, 1.3) | 27.46 (18.69, 37.63) | 48.37 (33.56, 63.56) | 1.77 (1.67, 1.87) |
| Virgin Islands | 0.01 (0.01, 0.02) | 1.6 (1.14, 2.08) | 37.11 (27.06, 47.04) | 53.02 (37.83, 68.71) | 0.82 (0.55, 1.08) |
| Sierra Leone | 0.34 (0.25, 0.43) | 0.01 (0.01, 0.01) | 24.74 (18.65, 31.45) | 46.11 (32.83, 60.07) | 0.53 (0.29, 0.76) |
| Sri Lanka | 2.11 (1.55, 2.69) | 0 (0, 0) | 28.53 (20.93, 36.38) | 60.54 (46.08, 75.59) | 1.53 (1.36, 1.70) |
| Saint Kitts | 0.01 (0.01, 0.02) | 0.01 (0, 0.01) | 58.65 (35.43, 88.76) | 87.7 (65.35, 111.82) | 0.71 (0.67, 0.74) |
| Norway | 0.13 (0.07, 0.19) | 0.57 (0.41, 0.73) | 8.06 (4.67, 11.68) | 50.84 (36.21, 65.3) | 1.28 (1.03, 1.52) |
| Egypt | 5.62 (4.05, 7.34) | 0.35 (0.26, 0.46) | 25.66 (18.47, 33.48) | 45.28 (33.4, 59.09) | 0.89 (0.64, 1.14) |
| Armenia | 0.49 (0.36, 0.64) | 39.05 (29.06, 49.23) | 33.87 (25.08, 44.8) | 36.87 (27.43, 46.47) | 0.79 (0.63, 0.95) |
| Italy | 2.17 (1.31, 3.17) | 0.11 (0.08, 0.13) | 10.16 (6.13, 14.84) | 49.8 (36.95, 62.57) | 2.81 (2.48, 3.15) |
| Micronesia (Federated States of) | 0.02 (0.02, 0.03) | 0.09 (0.06, 0.12) | 59.23 (45.31, 73.73) | 34.14 (23.07, 46.07) | 1.23 (0.83, 1.64) |
| Kazakhstan | 3.25 (2.53, 4.04) | 10.46 (7.87, 13.08) | 47.79 (37.23, 59.49) | 47.35 (35.61, 59.19) | 0.58 (0.45, 0.71) |
| Angola | 0.61 (0.44, 0.78) | 0.13 (0.08, 0.21) | 15.42 (11.08, 19.85) | 62.5 (37.16, 98.22) | 1.19 (1.07, 1.30) |
| Luxembourg | 0.02 (0.01, 0.02) | 1.15 (0.61, 1.7) | 11.23 (6.65, 16.37) | 8.85 (4.71, 13.11) | 0.39 (0.22, 0.55) |
| Germany | 3.76 (2.32, 5.47) | 1.04 (0.79, 1.28) | 12.67 (7.82, 18.43) | 76.49 (58.37, 94.19) | 0.47 (0.36, 0.58) |
| Togo | 0.39 (0.3, 0.49) | 42.36 (32.52, 52.68) | 28.6 (21.55, 35.84) | 46.31 (35.56, 57.59) | 0.45 (0.29, 0.60) |
| Monaco | 0 (0, 0) | 3.83 (2.62, 5.26) | 10.81 (6.11, 16.01) | 85.41 (58.33, 117.22) | 0.84 (0.78, 0.91) |
| Botswana | 0.16 (0.12, 0.19) | 0.36 (0.28, 0.44) | 30.3 (22.99, 37.63) | 44.62 (34.46, 54.46) | 0.74 (0.63, 0.85) |
| Philippines | 7.52 (5.57, 9.47) | 0.1 (0.08, 0.13) | 28.95 (21.44, 36.48) | 57.82 (44.26, 72.25) | 0.79 (0.73, 0.86) |
| Somalia | 0.46 (0.35, 0.59) | 0.19 (0.12, 0.26) | 18.41 (13.75, 23.36) | 11.55 (7.4, 15.78) | 2.11 (2.00, 2.22) |
| Senegal | 0.79 (0.59, 0.98) | 0.31 (0.22, 0.4) | 28.8 (21.57, 35.71) | 60.44 (43.73, 77.57) | 0.26 (0.13, 0.38) |
| Vietnam | 5.9 (4.25, 7.54) | 1.38 (1.04, 1.72) | 20.7 (14.89, 26.44) | 33.66 (25.52, 42.06) | 1.32 (1.24, 1.40) |
| Kyrgyzstan | 1.14 (0.9, 1.38) | 5.78 (4.06, 7.51) | 63.2 (49.61, 76.54) | 33.49 (23.54, 43.52) | 1.44 (1.15, 1.74) |
| Fiji | 0.14 (0.11, 0.17) | 1.97 (1.52, 2.53) | 43.1 (33.02, 53.8) | 48.71 (37.55, 62.41) | 2.66 (2.49, 2.83) |
| Iraq | 2.19 (1.59, 2.82) | 6.04 (4.11, 8.03) | 33.33 (24.12, 42.91) | 13.61 (9.26, 18.1) | 0.59 (0.54, 0.64) |
| Azerbaijan | 1.27 (0.94, 1.63) | 4.22 (3, 5.51) | 39.85 (29.47, 51.18) | 32.42 (23.03, 42.39) | 0.09 (0.00, 0.18) |
| Bulgaria | 1 (0.72, 1.31) | 0.54 (0.39, 0.7) | 33.66 (24.03, 43.89) | 41.14 (30.08, 53.67) | 1.27 (1.17, 1.37) |
| Central African Republic | 0.2 (0.15, 0.26) | 1.56 (1.12, 2.02) | 19.37 (14.18, 24.72) | 42.96 (30.75, 55.71) | 0.81 (0.70, 0.93) |
| Paraguay | 0.4 (0.28, 0.52) | 21.04 (13.91, 28.63) | 25.32 (18.01, 32.95) | 19.12 (12.65, 26.02) | 0.90 (0.85, 0.95) |
| Algeria | 2.6 (1.83, 3.46) | 0.68 (0.51, 0.87) | 25.69 (18.06, 34.16) | 34.4 (25.64, 43.66) | 0.89 (0.84, 0.94) |
| Singapore | 0.35 (0.24, 0.45) | 0.05 (0.04, 0.06) | 22.87 (16.16, 30) | 61.32 (48.28, 74.6) | 1.45 (1.23, 1.68) |
| San Marino | 0 (0, 0) | 0.02 (0.01, 0.02) | 8.89 (5.11, 13.31) | 61.6 (45.89, 77.96) | 0.34 (0.15, 0.53) |
| Switzerland | 0.34 (0.21, 0.49) | 0.06 (0.04, 0.08) | 12.88 (7.95, 18.53) | 31.17 (21.2, 41.85) | 0.66 (0.59, 0.73) |
| Greece | 0.49 (0.31, 0.69) | 2.4 (1.51, 3.39) | 13.01 (8.36, 18.32) | 12.08 (7.58, 17.03) | 0.58 (0.54, 0.62) |
| Thailand | 9.41 (6.8, 11.91) | 0.13 (0.09, 0.17) | 36.33 (26.25, 46) | 19.75 (13.66, 26.05) | 0.44 (0.26, 0.62) |
| Timor-Leste | 0.08 (0.06, 0.1) | 3.71 (2.59, 4.87) | 23.91 (17.46, 30.59) | 17.11 (11.96, 22.46) | 1.15 (1.03, 1.28) |
| Tanzania | 1.19 (0.82, 1.6) | 2.28 (1.73, 2.85) | 12.25 (8.45, 16.51) | 36.83 (27.97, 46.01) | 0.56 (0.41, 0.72) |
| Uganda | 0.64 (0.41, 0.85) | 0.21 (0.15, 0.27) | 9.99 (6.44, 13.32) | 27.75 (19.68, 35.77) | 0.91 (0.75, 1.06) |
| South Africa | 5.86 (4.38, 7.31) | 1.8 (1.06, 2.62) | 36.85 (27.56, 46) | 11.16 (6.6, 16.25) | 1.34 (1.21, 1.47) |
| Australia | 0.72 (0.43, 1.04) | 0.66 (0.44, 0.88) | 10.57 (6.35, 15.33) | 14.21 (9.52, 19.07) | 1.19 (1.09, 1.28) |
| Iceland | 0.01 (0.01, 0.01) | 0.82 (0.54, 1.08) | 9.87 (5.8, 13.77) | 15.17 (9.92, 19.96) | 2.21 (2.11, 2.31) |
| Argentina | 2.06 (1.38, 2.8) | 1.7 (1.29, 2.11) | 16.88 (11.32, 22.9) | 34.7 (26.32, 43.21) | 0.73 (0.63, 0.83) |
| Reunion | 0 (0, 0) | 0.37 (0.21, 0.53) | 38.89 (29.46, 48.06) | 12.04 (7.06, 17.42) | 0.94 (0.87, 1.01) |
| Chile | 0.77 (0.49, 1.1) | 0.02 (0.01, 0.02) | 13.45 (8.51, 19.2) | 48.18 (34.89, 61.38) | 0.31 (0.15, 0.47) |

Supplementary Table 2. Early onset CKD burden of the 204 countries, including death case number and rates in 1990 and 2019, and the estimated annual percentage changes (EAPCs, %) of death rate from 1990 to 2019.

| **Location name** | **Case 1990** | **Case 2019** | **Rate 1990 (per 100k)** | **Rate 2019 (100k)** | **EAPC 1990-2019 (%)** |
| --- | --- | --- | --- | --- | --- |
| Afghanistan | 224 (128, 328) | 605 (403, 950) | 5.8 (3.32, 8.47) | 4.81 (3.16, 6.9) | -4.24 (-4.50, -3.97) |
| Albania | 36 (32, 41) | 19 (14, 26) | 2.55 (2.25, 2.9) | 3.26 (2.2, 4.64) | -0.08 (-0.15, -0.01) |
| Algeria | 306 (213, 461) | 372 (277, 513) | 3.02 (2.1, 4.55) | 3.2 (1.87, 4.88) | 0.37 (0.11, 0.63) |
| American Samoa | 1 (1, 1) | 2 (1, 2) | 4.78 (3.74, 6.02) | 0.34 (0.29, 0.39) | -0.47 (-0.72, -0.22) |
| Andorra | 0 (0, 0) | 0 (0, 0) | 0.39 (0.27, 0.53) | 6 (4.75, 7.47) | 1.07 (0.08, 2.06) |
| Angola | 178 (122, 238) | 397 (260, 533) | 4.53 (3.11, 6.05) | 1.06 (0.81, 1.35) | -2.55 (-2.90, -2.21) |
| Antigua | 1 (1, 1) | 2 (1, 2) | 3.5 (3, 3.97) | 4.43 (3.55, 5.45) | 1.10 (0.94, 1.25) |
| Argentina | 253 (232, 274) | 302 (263, 346) | 2.07 (1.9, 2.24) | 4.23 (2.88, 5.63) | 0.00 (-0.32, 0.32) |
| Armenia | 4 (3, 4) | 15 (12, 18) | 0.25 (0.21, 0.3) | 12.87 (9, 17.56) | 2.71 (2.15, 3.27) |
| Australia | 19 (17, 21) | 23 (19, 27) | 0.27 (0.24, 0.31) | 0.51 (0.44, 0.6) | -0.86 (-1.03, -0.69) |
| Austria | 7 (6, 8) | 9 (8, 11) | 0.24 (0.21, 0.27) | 4.45 (2.63, 7.01) | -1.24 (-1.55, -0.93) |
| Azerbaijan | 121 (100, 146) | 171 (138, 210) | 3.82 (3.14, 4.58) | 7.59 (6.27, 8.97) | -0.06 (-0.14, 0.01) |
| Bahamas | 5 (4, 6) | 9 (7, 12) | 4.13 (3.5, 4.8) | 3.3 (2.42, 4.41) | 1.26 (0.84, 1.68) |
| Bahrain | 3 (2, 3) | 7 (5, 8) | 0.98 (0.77, 1.31) | 5.06 (3.47, 7.44) | -0.16 (-0.27, -0.04) |
| Bangladesh | 618 (407, 814) | 724 (551, 936) | 1.45 (0.95, 1.91) | 1.32 (1.05, 1.61) | -0.61 (-1.12, -0.10) |
| Barbados | 3 (3, 4) | 4 (3, 5) | 2.85 (2.48, 3.28) | 3.08 (2.07, 4.24) | 1.94 (1.74, 2.14) |
| Belarus | 10 (8, 11) | 22 (16, 29) | 0.24 (0.21, 0.28) | 4.58 (3.3, 6.09) | 2.71 (1.94, 3.49) |
| Belgium | 11 (9, 12) | 8 (6, 9) | 0.28 (0.25, 0.32) | 3.67 (2.61, 5.01) | -0.04 (-0.11, 0.04) |
| Belize | 2 (2, 2) | 12 (10, 15) | 2.83 (2.44, 3.29) | 5.47 (3.56, 7.83) | -1.52 (-1.69, -1.35) |
| Benin | 91 (72, 113) | 254 (168, 365) | 5.35 (4.21, 6.64) | 1.58 (1.23, 1.98) | -1.08 (-1.37, -0.78) |
| Bermuda | 0 (0, 1) | 0 (0, 0) | 1.79 (1.55, 2.07) | 0.25 (0.22, 0.29) | 3.05 (2.58, 3.52) |
| Bhutan | 9 (5, 13) | 12 (7, 18) | 3.41 (2.02, 4.95) | 3.11 (0.91, 4.32) | -0.19 (-0.29, -0.09) |
| Bolivia | 104 (82, 139) | 159 (100, 227) | 4.22 (3.33, 5.64) | 2.33 (1.69, 3.13) | 0.67 (0.59, 0.74) |
| Bosnia and Herzegovina | 22 (19, 25) | 11 (8, 14) | 1.14 (0.99, 1.3) | 5.19 (3.44, 7.47) | -1.73 (-2.10, -1.35) |
| Botswana | 15 (9, 23) | 47 (28, 74) | 2.89 (1.84, 4.5) | 3.84 (1.91, 5.52) | -0.64 (-0.74, -0.55) |
| Brazil | 1871 (1800, 1943) | 1507 (1433, 1584) | 2.98 (2.87, 3.1) | 3.45 (2.48, 4.65) | 0.40 (-0.02, 0.81) |
| Brunei | 4 (4, 5) | 6 (5, 7) | 3.61 (2.97, 4.27) | 6.9 (5.3, 8.93) | -0.54 (-1.01, -0.06) |
| Bulgaria | 51 (46, 56) | 62 (47, 82) | 1.7 (1.54, 1.88) | 2.91 (1.82, 4.53) | -3.43 (-3.88, -2.97) |
| Burkina Faso | 122 (86, 161) | 373 (252, 516) | 3.81 (2.7, 5.04) | 4.02 (2.68, 6.3) | 0.31 (0.11, 0.51) |
| Burundi | 99 (71, 135) | 161 (118, 208) | 4.78 (3.41, 6.51) | 2.98 (2.1, 4.48) | 2.03 (1.85, 2.22) |
| Cambodia | 236 (184, 297) | 275 (206, 357) | 6.08 (4.74, 7.66) | 1.46 (0.86, 2.09) | 1.44 (1.29, 1.59) |
| Cameroon | 276 (202, 357) | 856 (552, 1207) | 7.26 (5.32, 9.4) | 1.53 (1.02, 2.11) | -0.77 (-0.91, -0.63) |
| Canada | 36 (33, 40) | 40 (34, 46) | 0.33 (0.29, 0.36) | 0.43 (0.32, 0.58) | 1.47 (1.31, 1.63) |
| Cape Verde | 5 (4, 5) | 8 (6, 10) | 3.6 (3.1, 4.15) | 7.64 (5.39, 10.25) | 1.24 (0.85, 1.63) |
| Central African Republic | 54 (38, 73) | 107 (73, 157) | 5.18 (3.62, 6.99) | 1.81 (1.06, 2.65) | -0.30 (-0.39, -0.22) |
| Chad | 103 (73, 151) | 282 (187, 396) | 4.91 (3.49, 7.19) | 2.26 (1.54, 3.14) | -1.38 (-1.48, -1.27) |
| Chile | 63 (56, 69) | 60 (52, 70) | 1.1 (0.98, 1.21) | 5.36 (4.07, 7.1) | -0.79 (-0.93, -0.65) |
| China | 15543 (13285, 18132) | 7287 (6163, 8455) | 2.83 (2.42, 3.3) | 0.42 (0.36, 0.5) | -0.05 (-0.41, 0.32) |
| Colombia | 453 (423, 485) | 308 (232, 408) | 3.22 (3.01, 3.45) | 6.01 (4.32, 8.18) | 1.99 (1.56, 2.42) |
| Comoros | 6 (1, 9) | 9 (5, 13) | 3.24 (0.48, 5.11) | 0.46 (0.39, 0.54) | 1.59 (1.33, 1.85) |
| Cook Islands | 0 (0, 0) | 0 (0, 0) | 2.85 (2.04, 4.15) | 1.46 (1.24, 1.7) | -0.97 (-1.31, -0.62) |
| Costa Rica | 17 (15, 20) | 50 (38, 66) | 1.36 (1.2, 1.53) | 2.17 (1.61, 2.99) | -0.84 (-1.10, -0.58) |
| Croatia | 10 (9, 12) | 6 (4, 7) | 0.56 (0.49, 0.65) | 5.55 (4.17, 7.87) | -3.72 (-4.14, -3.29) |
| Cuba | 94 (85, 103) | 79 (62, 100) | 1.93 (1.75, 2.13) | 2.7 (2.21, 3.34) | 0.22 (-0.12, 0.55) |
| Cyprus | 1 (1, 2) | 2 (1, 2) | 0.46 (0.37, 0.57) | 4.73 (3.02, 6.79) | -2.55 (-2.69, -2.42) |
| Czech Republic | 29 (26, 33) | 10 (8, 13) | 0.78 (0.69, 0.89) | 2.11 (1.7, 2.54) | -0.33 (-0.49, -0.16) |
| Democratic Republic of the Congo | 621 (477, 792) | 1201 (906, 1531) | 4.34 (3.34, 5.54) | 7.24 (5.35, 9.62) | 4.20 (3.63, 4.77) |
| Denmark | 5 (5, 6) | 6 (5, 7) | 0.28 (0.24, 0.32) | 5.06 (4.25, 5.97) | -1.44 (-1.88, -1.00) |
| Djibouti | 6 (4, 8) | 17 (10, 25) | 2.86 (1.89, 4.01) | 2.61 (2.15, 3.16) | 0.38 (0.09, 0.68) |
| Dominica | 2 (1, 2) | 2 (2, 3) | 5.18 (4.31, 6.16) | 3.87 (2.9, 5.02) | -1.05 (-1.24, -0.85) |
| Dominican Republic | 84 (73, 97) | 193 (125, 270) | 2.73 (2.36, 3.15) | 2.52 (1.98, 3.2) | -0.65 (-1.05, -0.24) |
| Ecuador | 127 (115, 140) | 264 (201, 342) | 3.09 (2.8, 3.39) | 1.23 (0.93, 1.58) | 0.50 (0.35, 0.66) |
| Egypt | 492 (328, 609) | 741 (434, 1089) | 2.24 (1.5, 2.78) | 5.44 (3.5, 7.37) | -2.15 (-2.57, -1.74) |
| El Salvador | 71 (64, 79) | 256 (188, 344) | 3.4 (3.05, 3.81) | 3.28 (2.35, 4.63) | -1.16 (-1.41, -0.90) |
| Equatorial Guinea | 7 (5, 10) | 23 (14, 36) | 4.98 (3.41, 6.88) | 3.2 (1.53, 4.53) | 0.62 (0.15, 1.09) |
| Eritrea | 44 (28, 61) | 114 (68, 181) | 3.8 (2.43, 5.23) | 0.58 (0.45, 0.74) | -3.01 (-3.55, -2.46) |
| Estonia | 1 (1, 1) | 3 (2, 3) | 0.22 (0.2, 0.25) | 2.38 (1.43, 3.46) | 0.05 (-0.10, 0.20) |
| Ethiopia | 1144 (806, 1399) | 1200 (982, 1482) | 6.13 (4.32, 7.5) | 0.21 (0.18, 0.26) | 0.92 (0.65, 1.18) |
| Fiji | 16 (12, 21) | 24 (18, 31) | 5 (3.86, 6.43) | 0.54 (0.45, 0.64) | -1.50 (-2.01, -1.00) |
| Finland | 4 (4, 5) | 4 (3, 4) | 0.23 (0.2, 0.26) | 2.16 (1.63, 2.77) | -0.61 (-0.99, -0.23) |
| France | 55 (49, 62) | 29 (24, 34) | 0.25 (0.22, 0.28) | 0.91 (0.72, 1.13) | 0.03 (-0.36, 0.41) |
| Gabon | 20 (14, 27) | 35 (22, 51) | 5.26 (3.68, 7.02) | 3.02 (2.38, 3.82) | -0.33 (-0.61, -0.04) |
| Gambia | 17 (11, 24) | 45 (31, 63) | 4.39 (2.87, 6.25) | 3.18 (2.22, 4.48) | -2.73 (-3.28, -2.17) |
| Georgia | 39 (33, 45) | 31 (25, 37) | 1.82 (1.53, 2.12) | 0.18 (0.15, 0.21) | -1.11 (-2.07, -0.13) |
| Germany | 107 (98, 118) | 59 (50, 68) | 0.36 (0.33, 0.4) | 3.6 (2.7, 4.8) | 1.44 (1.22, 1.66) |
| Ghana | 291 (197, 396) | 741 (477, 1003) | 5.06 (3.42, 6.9) | 3.32 (1.97, 4.96) | 4.66 (3.36, 5.98) |
| Greece | 26 (23, 29) | 13 (11, 15) | 0.69 (0.61, 0.77) | 0.54 (0.42, 0.75) | -1.38 (-1.83, -0.93) |
| Greenland | 0 (0, 0) | 0 (0, 0) | 0.36 (0.25, 0.49) | 8.68 (6.76, 11.02) | -1.28 (-1.62, -0.94) |
| Grenada | 2 (2, 2) | 3 (2, 3) | 5.56 (4.73, 6.48) | 8.11 (6.1, 10.58) | -0.71 (-0.85, -0.58) |
| Guam | 2 (1, 2) | 3 (3, 4) | 2.38 (2.02, 2.87) | 6.37 (4.86, 8.27) | 1.67 (1.35, 2.00) |
| Guatemala | 152 (133, 173) | 549 (423, 707) | 5.23 (4.59, 5.97) | 2.61 (1.88, 3.58) | -1.45 (-1.66, -1.24) |
| Guinea | 138 (104, 184) | 290 (208, 395) | 6.38 (4.81, 8.51) | 0.36 (0.29, 0.43) | 0.90 (0.53, 1.27) |
| Guinea-Bissau | 36 (25, 47) | 61 (44, 82) | 9.52 (6.7, 12.57) | 2.01 (1.69, 2.4) | 4.17 (3.15, 5.20) |
| Guyana | 15 (12, 18) | 25 (17, 33) | 4.54 (3.64, 5.4) | 3.98 (2.37, 6.31) | 2.01 (1.58, 2.44) |
| Haiti | 148 (95, 218) | 273 (183, 417) | 6.1 (3.9, 8.99) | 1.99 (1.44, 2.68) | -0.25 (-0.54, 0.04) |
| Honduras | 76 (59, 93) | 135 (79, 206) | 4.4 (3.39, 5.39) | 3.5 (2.06, 5.22) | -2.77 (-3.31, -2.22) |
| Hungary | 37 (33, 42) | 17 (13, 21) | 1.01 (0.9, 1.13) | 2.63 (2.01, 3.42) | -0.52 (-0.60, -0.45) |
| Iceland | 0 (0, 0) | 0 (0, 0) | 0.16 (0.14, 0.19) | 0.71 (0.53, 0.94) | -4.06 (-4.95, -3.15) |
| India | 12110 (10332, 13749) | 20122 (17242, 23345) | 3.55 (3.03, 4.03) | 0.58 (0.44, 0.77) | -1.70 (-1.91, -1.49) |
| Indonesia | 4484 (3873, 5082) | 4905 (4214, 6094) | 5.74 (4.96, 6.51) | 1.98 (1.12, 3.17) | -1.83 (-2.19, -1.47) |
| Iran | 378 (336, 415) | 526 (490, 562) | 1.67 (1.49, 1.84) | 6.92 (3.55, 11.46) | -0.79 (-1.35, -0.24) |
| Iraq | 234 (167, 322) | 445 (267, 646) | 3.57 (2.54, 4.89) | 0.27 (0.19, 0.38) | -0.02 (-0.10, 0.06) |
| Ireland | 4 (4, 5) | 4 (3, 4) | 0.32 (0.28, 0.36) | 0.34 (0.26, 0.43) | -0.05 (-0.26, 0.17) |
| Israel | 10 (8, 11) | 14 (12, 16) | 0.5 (0.44, 0.57) | 9.1 (5.93, 12.83) | -0.97 (-1.22, -0.71) |
| Italy | 85 (82, 89) | 33 (31, 35) | 0.4 (0.38, 0.42) | 2.19 (1.72, 2.77) | 0.60 (0.30, 0.90) |
| Ivory Coast | 298 (219, 382) | 628 (413, 865) | 6.3 (4.62, 8.07) | 4.68 (3.51, 5.93) | 0.00 (-0.09, 0.09) |
| Jamaica | 20 (18, 23) | 53 (39, 71) | 2.07 (1.8, 2.35) | 0.56 (0.5, 0.65) | -3.01 (-3.57, -2.44) |
| Japan | 250 (243, 257) | 75 (71, 79) | 0.56 (0.54, 0.57) | 10.77 (6.95, 15.11) | -2.93 (-3.09, -2.76) |
| Jordan | 33 (27, 40) | 84 (68, 104) | 2.14 (1.73, 2.57) | 0.15 (0.11, 0.2) | 1.72 (0.39, 3.06) |
| Kazakhstan | 227 (203, 254) | 147 (118, 176) | 3.34 (2.99, 3.73) | 6.59 (3.64, 9.77) | -3.54 (-4.46, -2.62) |
| Kenya | 212 (172, 265) | 655 (515, 828) | 2.42 (1.97, 3.03) | 8.97 (6.69, 11.57) | 1.00 (0.72, 1.29) |
| Kiribati | 3 (2, 4) | 6 (4, 9) | 9.33 (7.34, 11.88) | 1.69 (1.36, 2.07) | 1.21 (1.01, 1.41) |
| Kuwait | 14 (12, 16) | 15 (12, 19) | 1.64 (1.42, 1.89) | 6.79 (4.77, 9.04) | -3.95 (-4.64, -3.26) |
| Kyrgyzstan | 136 (117, 158) | 110 (92, 131) | 7.55 (6.48, 8.73) | 2.55 (2.03, 3.09) | -0.31 (-0.55, -0.07) |
| Laos | 131 (91, 173) | 185 (127, 243) | 8.5 (5.86, 11.16) | 5.59 (3.89, 7.6) | -1.12 (-1.28, -0.95) |
| Latvia | 2 (2, 2) | 6 (4, 7) | 0.2 (0.18, 0.23) | 2.55 (1.89, 3.3) | 5.47 (4.58, 6.37) |
| Lebanon | 27 (20, 33) | 32 (22, 44) | 2.21 (1.68, 2.76) | 3.66 (2.78, 4.74) | -2.16 (-2.48, -1.84) |
| Lesotho | 16 (11, 22) | 54 (32, 79) | 2.26 (1.63, 3.14) | 6.65 (4.95, 8.57) | 4.47 (3.99, 4.95) |
| Liberia | 45 (33, 58) | 109 (71, 155) | 6.67 (4.97, 8.73) | 4.02 (3.22, 4.92) | -0.39 (-0.83, 0.05) |
| Libya | 36 (27, 46) | 68 (47, 95) | 2.2 (1.65, 2.78) | 0.32 (0.26, 0.39) | -0.46 (-0.82, -0.09) |
| Lithuania | 4 (3, 4) | 6 (5, 7) | 0.26 (0.23, 0.29) | 15.01 (10.1, 19.85) | -0.30 (-0.87, 0.28) |
| Luxembourg | 1 (1, 1) | 1 (1, 1) | 0.42 (0.37, 0.48) | 1.01 (0.79, 1.28) | -1.80 (-1.98, -1.61) |
| Madagascar | 204 (149, 261) | 320 (237, 423) | 4.51 (3.29, 5.76) | 1.07 (0.81, 1.38) | -0.10 (-0.32, 0.12) |
| Malawi | 146 (102, 184) | 280 (199, 382) | 4.03 (2.82, 5.08) | 0.21 (0.19, 0.22) | -1.12 (-1.46, -0.78) |
| Malaysia | 219 (190, 251) | 297 (224, 382) | 2.96 (2.56, 3.38) | 3.05 (2.27, 4.02) | -1.00 (-1.12, -0.88) |
| Maldives | 4 (3, 5) | 6 (5, 8) | 5.19 (3.98, 6.53) | 1.48 (1.38, 1.58) | -2.57 (-2.96, -2.17) |
| Mali | 194 (142, 258) | 393 (258, 564) | 6.53 (4.77, 8.67) | 4.14 (3.47, 4.91) | -1.08 (-1.33, -0.83) |
| Malta | 1 (0, 1) | 1 (1, 1) | 0.4 (0.34, 0.45) | 0.17 (0.14, 0.19) | 0.33 (0.17, 0.50) |
| Marshall Islands | 1 (1, 1) | 3 (2, 4) | 6.49 (5.19, 8.23) | 0.17 (0.16, 0.18) | -0.53 (-0.79, -0.27) |
| Mauritania | 52 (41, 65) | 61 (37, 90) | 6.81 (5.35, 8.42) | 1.04 (0.79, 1.34) | -1.76 (-1.88, -1.63) |
| Mauritius | 25 (22, 28) | 41 (32, 52) | 5 (4.44, 5.6) | 0.5 (0.36, 0.67) | 1.04 (0.60, 1.49) |
| Mexico | 1263 (1229, 1303) | 3329 (2888, 3802) | 3.54 (3.45, 3.66) | 3.86 (2.77, 5.09) | 2.61 (2.46, 2.76) |
| Micronesia (Federated States of) | 4 (3, 5) | 6 (3, 8) | 9.03 (6.68, 12.19) | 0.69 (0.54, 0.89) | 2.50 (1.99, 3.00) |
| Moldova | 9 (8, 10) | 16 (14, 20) | 0.51 (0.45, 0.57) | 7.59 (5.5, 10.24) | 0.39 (0.23, 0.56) |
| Monaco | 0 (0, 0) | 0 (0, 0) | 0.23 (0.18, 0.29) | 5.67 (3.38, 8.36) | -0.57 (-0.76, -0.38) |
| Mongolia | 80 (64, 98) | 73 (55, 96) | 9.07 (7.3, 11.09) | 5.58 (4.49, 6.87) | -1.35 (-1.87, -0.84) |
| Montenegro | 5 (5, 6) | 4 (3, 4) | 2.15 (1.82, 2.5) | 0.36 (0.24, 0.49) | -5.65 (-6.01, -5.28) |
| Morocco | 222 (167, 280) | 284 (186, 452) | 2.13 (1.61, 2.69) | 0.23 (0.22, 0.24) | -0.20 (-0.84, 0.45) |
| Mozambique | 146 (109, 195) | 475 (323, 632) | 3.2 (2.39, 4.26) | 5.31 (3.42, 7.53) | 1.78 (1.64, 1.92) |
| Myanmar | 1643 (1177, 2134) | 1500 (1053, 1999) | 9.7 (6.95, 12.6) | 0.28 (0.23, 0.33) | -0.63 (-0.72, -0.55) |
| Namibia | 12 (7, 19) | 23 (12, 37) | 2.17 (1.25, 3.38) | 0.25 (0.24, 0.26) | -1.05 (-1.35, -0.74) |
| Nauru | 0 (0, 1) | 1 (0, 1) | 9.34 (6.08, 12.46) | 3.38 (2.89, 3.92) | -0.36 (-0.70, -0.02) |
| Nepal | 243 (173, 321) | 420 (261, 591) | 3.32 (2.37, 4.39) | 0.21 (0.18, 0.25) | -3.23 (-3.82, -2.62) |
| Netherlands | 14 (12, 15) | 9 (8, 11) | 0.22 (0.2, 0.25) | 4.77 (3.24, 6.67) | -1.45 (-1.69, -1.22) |
| New Zealand | 7 (6, 8) | 8 (7, 9) | 0.5 (0.45, 0.57) | 0.99 (0.93, 1.05) | -3.27 (-3.42, -3.11) |
| Nicaragua | 60 (54, 69) | 179 (136, 232) | 4.08 (3.64, 4.66) | 0.35 (0.29, 0.4) | -0.35 (-0.84, 0.15) |
| Niger | 148 (111, 196) | 341 (222, 477) | 5.33 (3.99, 7.04) | 5.13 (3.44, 7.84) | 0.02 (-0.20, 0.25) |
| Nigeria | 1347 (1000, 1834) | 2667 (1862, 3758) | 3.95 (2.94, 5.38) | 5.88 (4.04, 7.72) | 1.45 (0.61, 2.30) |
| Niue | 0 (0, 0) | 0 (0, 0) | 5.92 (3.87, 8.33) | 0.74 (0.59, 0.93) | -2.05 (-2.35, -1.74) |
| North Korea | 355 (319, 384) | 59 (51, 68) | 1.68 (1.52, 1.82) | 0.45 (0.39, 0.53) | 2.15 (1.79, 2.52) |
| North Macedonia | 12 (10, 14) | 9 (7, 12) | 1.45 (1.26, 1.67) | 3.77 (2.96, 4.86) | -1.47 (-1.76, -1.18) |
| Northern Mariana Islands | 2 (1, 2) | 1 (1, 1) | 6.8 (4.4, 9.26) | 7.09 (4.56, 9.99) | 1.57 (0.79, 2.35) |
| Norway | 4 (3, 4) | 3 (3, 3) | 0.22 (0.21, 0.23) | 4.2 (2.74, 5.87) | -0.08 (-0.43, 0.28) |
| Oman | 5 (4, 7) | 14 (11, 19) | 0.62 (0.44, 0.86) | 4.41 (3.25, 5.95) | -0.92 (-1.12, -0.72) |
| Pakistan | 1787 (1317, 2417) | 7415 (5104, 9655) | 4.36 (3.22, 5.9) | 1.68 (1.34, 2.08) | -0.01 (-0.21, 0.20) |
| Palau | 1 (0, 1) | 1 (1, 1) | 9.16 (6.18, 12.59) | 4.23 (3.06, 5.83) | -1.07 (-1.40, -0.74) |
| Palestine | 23 (17, 31) | 42 (35, 50) | 3.05 (2.24, 4.05) | 0.14 (0.12, 0.17) | -0.97 (-1.25, -0.69) |
| Panama | 21 (18, 23) | 53 (39, 70) | 2.04 (1.8, 2.31) | 0.33 (0.28, 0.39) | -1.07 (-1.67, -0.45) |
| Papua New Guinea | 52 (40, 66) | 147 (111, 196) | 3.16 (2.4, 3.96) | 5.59 (4.49, 6.86) | 1.44 (1.10, 1.77) |
| Paraguay | 25 (21, 29) | 79 (57, 107) | 1.59 (1.35, 1.86) | 3.48 (2.56, 4.51) | -1.81 (-1.92, -1.69) |
| Peru | 294 (249, 343) | 356 (256, 489) | 3.31 (2.8, 3.86) | 4.81 (3.36, 6.63) | 0.47 (0.12, 0.82) |
| Philippines | 1429 (1290, 1580) | 3501 (2894, 4139) | 5.51 (4.97, 6.09) | 0.62 (0.46, 0.82) | -0.71 (-1.17, -0.25) |
| Poland | 268 (259, 278) | 68 (57, 80) | 1.85 (1.79, 1.92) | 13.63 (6.53, 19.34) | -4.64 (-5.07, -4.20) |
| Portugal | 39 (35, 44) | 14 (12, 16) | 1.03 (0.92, 1.15) | 4.88 (3.23, 6.84) | 2.18 (1.79, 2.58) |
| Puerto Rico | 38 (34, 43) | 29 (21, 37) | 2.71 (2.4, 3.03) | 7.99 (5.85, 11) | -1.13 (-1.37, -0.89) |
| Qatar | 2 (2, 3) | 9 (6, 12) | 0.84 (0.64, 1.18) | 1.02 (0.87, 1.2) | -0.18 (-0.42, 0.06) |
| Republic of Congo | 55 (35, 79) | 85 (56, 124) | 5.8 (3.73, 8.29) | 5.79 (3.22, 10.24) | 0.44 (0.30, 0.58) |
| Reunion | 0 (0, 0) | 0 (0, 0) | 5.34 (3.4, 7.93) | 1.73 (1.51, 1.99) | 1.82 (1.59, 2.06) |
| Romania | 197 (183, 213) | 51 (40, 64) | 2.27 (2.1, 2.45) | 1.07 (0.92, 1.23) | 1.19 (0.81, 1.57) |
| Russia | 1326 (1285, 1376) | 515 (443, 591) | 2.28 (2.21, 2.37) | 3.43 (2.59, 4.38) | -4.31 (-4.89, -3.73) |
| Rwanda | 143 (107, 183) | 167 (112, 229) | 5.23 (3.91, 6.71) | 7.58 (6.08, 9.39) | 0.45 (-0.12, 1.03) |
| Saint Kitts | 1 (1, 1) | 1 (0, 2) | 7.33 (6.24, 8.64) | 5.83 (3.84, 8.04) | 0.07 (-0.37, 0.50) |
| Saint Lucia | 2 (2, 3) | 4 (3, 5) | 4.01 (3.51, 4.62) | 7.77 (4.62, 10.78) | 1.48 (1.27, 1.68) |
| Saint Vincent | 2 (1, 2) | 3 (2, 3) | 3.77 (3.2, 4.4) | 0.75 (0.57, 0.97) | -3.18 (-3.71, -2.66) |
| Samoa | 3 (2, 5) | 5 (3, 8) | 4.69 (3.24, 6.97) | 6.16 (4.74, 8.02) | -2.20 (-2.39, -2.01) |
| San Marino | 0 (0, 0) | 0 (0, 0) | 0.13 (0.11, 0.17) | 4.38 (2.96, 6.04) | -3.41 (-3.99, -2.84) |
| Sao Tome and Principe | 2 (1, 3) | 6 (5, 8) | 5.56 (2.5, 7.87) | 6.64 (5.29, 8.21) | 0.01 (-0.21, 0.24) |
| Saudi Arabia | 180 (129, 235) | 600 (430, 849) | 2.69 (1.93, 3.5) | 6.96 (5.75, 8.45) | -0.95 (-1.11, -0.79) |
| Senegal | 173 (126, 233) | 325 (210, 462) | 6.27 (4.55, 8.45) | 3.79 (2.29, 5.62) | 0.96 (0.59, 1.34) |
| Serbia | 46 (39, 53) | 21 (16, 27) | 1.32 (1.13, 1.54) | 9.92 (7.29, 13.35) | -0.84 (-1.42, -0.25) |
| Seychelles | 1 (1, 1) | 2 (2, 2) | 3.39 (2.91, 3.9) | 0.17 (0.11, 0.27) | 0.60 (0.32, 0.88) |
| Sierra Leone | 62 (43, 85) | 168 (117, 232) | 4.57 (3.17, 6.22) | 1.61 (1.21, 2.13) | 1.27 (0.92, 1.62) |
| Singapore | 22 (19, 25) | 11 (9, 13) | 1.46 (1.29, 1.64) | 1.53 (1.2, 1.88) | -2.03 (-2.15, -1.91) |
| Slovakia | 24 (21, 28) | 10 (8, 14) | 1.2 (1.02, 1.38) | 3.15 (2.36, 4.13) | -2.44 (-2.78, -2.10) |
| Slovenia | 2 (2, 3) | 1 (1, 1) | 0.29 (0.21, 0.39) | 4.28 (2.79, 6.01) | 0.84 (0.42, 1.27) |
| Solomon Islands | 18 (13, 24) | 23 (18, 30) | 13.63 (9.91, 18.42) | 3.21 (1.99, 4.52) | -2.57 (-3.12, -2.01) |
| Somalia | 109 (76, 149) | 332 (240, 457) | 4.32 (3.03, 5.9) | 4.02 (2.64, 5.88) | -2.75 (-3.13, -2.38) |
| South Africa | 845 (731, 950) | 1120 (840, 1420) | 5.31 (4.6, 5.97) | 0.16 (0.13, 0.19) | 0.85 (0.62, 1.09) |
| South Korea | 315 (207, 452) | 301 (199, 444) | 3.81 (2.51, 5.47) | 1.25 (1.04, 1.53) | 1.53 (1.30, 1.76) |
| South Sudan | 65 (43, 93) | 100 (62, 155) | 2.8 (1.83, 3.99) | 12.69 (7.71, 17.11) | 0.09 (-0.13, 0.31) |
| Spain | 111 (102, 122) | 33 (28, 38) | 0.75 (0.69, 0.82) | 2.29 (1.2, 3.66) | -0.03 (-0.22, 0.16) |
| Sri Lanka | 301 (237, 349) | 245 (181, 334) | 4.06 (3.2, 4.72) | 3.96 (2.51, 6.41) | 6.86 (5.96, 7.76) |
| Sudan | 184 (127, 256) | 341 (194, 547) | 2.41 (1.67, 3.36) | 3 (1.99, 4.42) | 1.73 (1.49, 1.97) |
| Suriname | 8 (5, 9) | 16 (13, 20) | 4.86 (3.33, 5.67) | 6.93 (5.35, 8.93) | 1.76 (1.49, 2.03) |
| Swaziland | 10 (8, 13) | 32 (18, 48) | 3.35 (2.52, 4.39) | 6.88 (5.63, 8.31) | 2.45 (1.67, 3.23) |
| Sweden | 6 (5, 6) | 5 (4, 6) | 0.19 (0.17, 0.21) | 0.23 (0.2, 0.27) | -0.48 (-0.68, -0.28) |
| Switzerland | 5 (5, 6) | 5 (4, 5) | 0.2 (0.18, 0.23) | 5.18 (3.45, 8.16) | -0.89 (-1.11, -0.67) |
| Syria | 244 (189, 307) | 128 (93, 172) | 5.07 (3.93, 6.37) | 2.64 (1.92, 3.61) | -0.24 (-1.19, 0.72) |
| Taiwan | 175 (162, 190) | 70 (53, 93) | 1.9 (1.75, 2.06) | 1.75 (1.66, 1.84) | -0.79 (-0.88, -0.70) |
| Tajikistan | 32 (28, 36) | 102 (80, 130) | 1.5 (1.33, 1.7) | 0.54 (0.45, 0.66) | -0.89 (-1.73, -0.03) |
| Tanzania | 320 (248, 408) | 713 (512, 957) | 3.31 (2.57, 4.22) | 3.58 (2.17, 5.51) | -0.09 (-0.19, 0.01) |
| Thailand | 1724 (1380, 2244) | 936 (672, 1235) | 6.66 (5.33, 8.66) | 6.81 (3.99, 10.73) | 2.19 (1.87, 2.51) |
| Timor-Leste | 14 (9, 19) | 17 (5, 23) | 4.39 (2.69, 6.08) | 3.04 (2.25, 4.15) | -1.49 (-2.07, -0.91) |
| Togo | 75 (55, 99) | 166 (113, 221) | 5.47 (4, 7.24) | 1.54 (1.05, 2.15) | 3.00 (2.76, 3.25) |
| Tonga | 1 (1, 2) | 2 (1, 3) | 2.94 (2.16, 4.7) | 0.31 (0.26, 0.37) | -0.66 (-1.10, -0.21) |
| Trinidad | 17 (15, 19) | 29 (20, 39) | 3.34 (2.92, 3.8) | 0.32 (0.24, 0.43) | -1.12 (-1.23, -1.00) |
| Tunisia | 52 (43, 63) | 68 (45, 94) | 1.51 (1.25, 1.82) | 3.3 (2.07, 4.7) | -3.58 (-4.23, -2.93) |
| Turkey | 601 (495, 768) | 375 (290, 468) | 2.43 (2, 3.11) | 3.23 (2.32, 4.33) | -1.48 (-1.70, -1.26) |
| Turkmenistan | 81 (72, 91) | 140 (108, 182) | 5.25 (4.66, 5.9) | 6.63 (5.75, 7.58) | 1.94 (1.47, 2.40) |
| Tuvalu | 0 (0, 0) | 0 (0, 1) | 7.43 (5.35, 10.18) | 3.31 (2.44, 4.23) | 0.73 (0.52, 0.94) |
| Uganda | 167 (115, 232) | 527 (355, 749) | 2.6 (1.78, 3.61) | 1.1 (0.85, 1.37) | -0.55 (-0.83, -0.27) |
| UK | 60 (58, 61) | 55 (53, 57) | 0.29 (0.28, 0.29) | 8.11 (5.58, 10.56) | 0.24 (-0.03, 0.50) |
| Ukraine | 56 (50, 63) | 221 (174, 271) | 0.29 (0.26, 0.33) | 2.93 (2.17, 3.87) | 1.41 (1.18, 1.63) |
| United Arab Emirates | 40 (29, 58) | 259 (145, 459) | 4.2 (3.08, 6.05) | 3.55 (2.32, 4.75) | 1.04 (0.50, 1.58) |
| Uruguay | 10 (9, 11) | 12 (11, 14) | 0.87 (0.76, 0.99) | 0.89 (0.67, 1.17) | -1.11 (-1.34, -0.89) |
| USA | 830 (807, 853) | 1092 (1027, 1152) | 0.81 (0.79, 0.84) | 0.16 (0.14, 0.19) | 0.57 (0.03, 1.12) |
| Uzbekistan | 435 (369, 547) | 982 (802, 1185) | 5.07 (4.3, 6.36) | 1.95 (1.27, 3.09) | 1.10 (0.87, 1.34) |
| Vanuatu | 2 (1, 4) | 9 (5, 13) | 4.13 (2.53, 6.11) | 5.21 (3.53, 6.93) | 0.28 (-0.21, 0.78) |
| Venezuela | 156 (142, 172) | 367 (264, 495) | 1.95 (1.77, 2.14) | 4.63 (3.98, 5.75) | 1.18 (0.64, 1.73) |
| Vietnam | 1124 (794, 1548) | 1305 (961, 1668) | 3.94 (2.78, 5.43) | 0.89 (0.76, 1.02) | 1.01 (0.71, 1.31) |
| Virgin Islands | 1 (1, 2) | 1 (1, 1) | 3.36 (2.7, 4.11) | 6.31 (3.61, 9.42) | 0.16 (0.01, 0.32) |
| Yemen | 71 (36, 112) | 189 (112, 272) | 1.54 (0.78, 2.43) | 0.23 (0.19, 0.27) | 2.27 (1.99, 2.55) |
| Zambia | 144 (116, 177) | 348 (251, 462) | 4.73 (3.83, 5.84) | 4.6 (1.44, 7.71) | -0.88 (-0.96, -0.81) |
| Zimbabwe | 83 (55, 143) | 245 (155, 398) | 2.11 (1.38, 3.61) | 2.99 (2.5, 3.62) | 0.52 (0.21, 0.82) |

Supplementary Table 3. Early onset CKD burden of the 204 countries, including DALY case number and rates in 1990 and 2019, and the estimated annual percentage changes (EAPCs, %) of incidence rate from 1990 to 2019.

| **Location name** | **Case 1990 (x 1000)** | **Case 2019 (x 1000)** | **Rate 1990 (per 100k)** | **Rate 2019 (per 100k)** | **EAPC 1990-2019 (%)** |
| --- | --- | --- | --- | --- | --- |
| Afghanistan | 16.08 (10.46, 22.66) | 49.4 (37.03, 71.14) | 415.18 (270.23, 585.32) | 47.43 (35.36, 62.08) | 1.48 (1.31, 1.65) |
| Albania | 2.8 (2.46, 3.18) | 1.65 (1.29, 2.1) | 197.15 (173.39, 224.54) | 51.63 (40.06, 67.83) | 1.74 (1.36, 2.11) |
| Algeria | 24.48 (18.49, 34.12) | 38.03 (29.89, 47.9) | 241.78 (182.55, 336.97) | 131.17 (95.41, 179.02) | -0.49 (-0.54, -0.43) |
| American Samoa | 0.08 (0.06, 0.1) | 0.13 (0.1, 0.17) | 388.07 (314.27, 474.55) | 249.03 (195.57, 312.77) | -2.15 (-2.34, -1.97) |
| Andorra | 0.01 (0.01, 0.02) | 0.01 (0.01, 0.02) | 49.41 (37.72, 63.39) | 326.83 (232.56, 433.13) | -0.95 (-1.14, -0.76) |
| Angola | 12.57 (9, 16.38) | 30.13 (21.24, 38.64) | 319.5 (228.85, 416.2) | 585.76 (483.59, 706.65) | -2.64 (-2.95, -2.32) |
| Antigua | 0.08 (0.07, 0.09) | 0.14 (0.11, 0.16) | 297.2 (255.57, 344.47) | 563.44 (358.81, 838.97) | -0.43 (-0.51, -0.35) |
| Argentina | 20.49 (18.27, 23) | 26.02 (22.19, 30.16) | 167.66 (149.49, 188.21) | 322.19 (227.87, 428.2) | -0.19 (-0.27, -0.12) |
| Armenia | 0.94 (0.66, 1.3) | 1.65 (1.29, 2.07) | 65.48 (45.81, 90.21) | 126.09 (102.95, 156.48) | 1.42 (1.28, 1.56) |
| Australia | 2.91 (2.32, 3.77) | 3.97 (2.99, 5.11) | 43.01 (34.21, 55.7) | 78.59 (60.41, 99.91) | -2.30 (-2.61, -1.99) |
| Austria | 1.34 (0.97, 1.82) | 1.52 (1.12, 2.04) | 44.8 (32.17, 60.55) | 150.34 (122.17, 183.68) | 0.60 (0.46, 0.75) |
| Azerbaijan | 9.54 (8.07, 11.22) | 14.22 (11.7, 17.14) | 299.92 (253.58, 352.75) | 314.29 (240.92, 392.33) | 1.00 (0.70, 1.30) |
| Bahamas | 0.39 (0.33, 0.45) | 0.73 (0.59, 0.91) | 329.05 (280.05, 381.58) | 1048.18 (767.92, 1331.32) | -0.22 (-0.36, -0.08) |
| Bahrain | 0.3 (0.24, 0.39) | 0.95 (0.71, 1.27) | 117.56 (93.02, 150.51) | 400.82 (207.83, 583.52) | 0.51 (0.40, 0.62) |
| Bangladesh | 49.63 (35.42, 62.65) | 72.82 (57.27, 90.87) | 116.27 (82.98, 146.78) | 172.14 (131.2, 221.86) | 0.71 (0.45, 0.97) |
| Barbados | 0.27 (0.23, 0.32) | 0.34 (0.27, 0.42) | 249.36 (213.39, 293.51) | 268.96 (189.53, 344.87) | -0.59 (-0.71, -0.47) |
| Belarus | 2.18 (1.57, 2.97) | 2.92 (2.17, 3.85) | 55.11 (39.7, 75.05) | 327.99 (251.53, 415.58) | 2.48 (2.04, 2.92) |
| Belgium | 1.84 (1.38, 2.38) | 1.58 (1.16, 2.12) | 49.51 (37.11, 64.07) | 643.06 (509.35, 808.25) | -2.13 (-2.55, -1.70) |
| Belize | 0.19 (0.16, 0.22) | 1 (0.84, 1.19) | 255.76 (217.61, 301.14) | 365.45 (266.93, 513.46) | 0.55 (0.29, 0.82) |
| Benin | 6.41 (5.21, 7.82) | 18.59 (13.05, 25.55) | 376.47 (306.11, 459.1) | 56.77 (44.99, 70.69) | 1.38 (0.94, 1.82) |
| Bermuda | 0.04 (0.04, 0.05) | 0.03 (0.03, 0.04) | 173.38 (144.23, 209.64) | 59.01 (46.58, 75.96) | 0.66 (0.26, 1.05) |
| Bhutan | 0.66 (0.43, 0.91) | 0.97 (0.66, 1.35) | 253.77 (164.06, 351.13) | 354.16 (259.57, 470.58) | -0.48 (-0.68, -0.28) |
| Bolivia | 7.55 (6.16, 9.55) | 12.91 (9.01, 16.92) | 306.51 (250.06, 387.84) | 305.66 (244.62, 383.92) | -0.61 (-0.71, -0.51) |
| Bosnia and Herzegovina | 2.05 (1.74, 2.42) | 1.16 (0.91, 1.44) | 105.89 (90.22, 125.27) | 687.47 (549.26, 843.85) | 1.28 (0.93, 1.62) |
| Botswana | 1.14 (0.8, 1.6) | 3.52 (2.34, 5.11) | 220.67 (154.74, 311.11) | 358.73 (252.71, 484.12) | 0.03 (-0.35, 0.41) |
| Brazil | 147.47 (134.21, 163.96) | 149.19 (127.26, 176.48) | 235.01 (213.88, 261.28) | 51.84 (38.96, 67.91) | 1.24 (0.99, 1.49) |
| Brunei | 0.32 (0.27, 0.37) | 0.43 (0.37, 0.5) | 256.64 (217.45, 297.57) | 298.6 (227.65, 397.85) | -1.23 (-1.51, -0.94) |
| Bulgaria | 4.22 (3.68, 4.88) | 4.87 (3.91, 6.08) | 141.94 (123.74, 163.9) | 459.48 (299.85, 646.75) | 1.89 (1.56, 2.23) |
| Burkina Faso | 8.86 (6.61, 11.29) | 27.9 (19.85, 36.97) | 277.53 (206.86, 353.61) | 147.87 (115.57, 185.36) | -0.58 (-0.83, -0.33) |
| Burundi | 7.04 (5.19, 9.28) | 12 (9.17, 14.94) | 339.62 (250.6, 447.9) | 42.4 (34.49, 52.39) | 0.71 (0.58, 0.84) |
| Cambodia | 16.87 (13.71, 20.42) | 22.75 (18.12, 27.99) | 435.22 (353.67, 526.72) | 237.09 (173.39, 312.26) | -1.21 (-1.41, -1.00) |
| Cameroon | 19.25 (14.71, 24.15) | 62.9 (43.25, 85.03) | 506.69 (387.33, 635.67) | 525.58 (419.81, 662.52) | 1.12 (0.64, 1.59) |
| Canada | 5.65 (4.35, 7.26) | 6.42 (4.92, 8.28) | 50.8 (39.16, 65.34) | 59.48 (47.32, 75.86) | -2.94 (-3.51, -2.37) |
| Cape Verde | 0.34 (0.3, 0.39) | 0.62 (0.49, 0.78) | 262.72 (227.31, 299.24) | 449.03 (367.8, 543.12) | 1.49 (1.30, 1.68) |
| Central African Republic | 3.83 (2.78, 5.04) | 7.71 (5.63, 10.83) | 364.91 (264.95, 479.58) | 255.21 (151.65, 340.81) | -2.22 (-2.55, -1.89) |
| Chad | 7.24 (5.32, 10.06) | 20.7 (14.71, 27.67) | 345.13 (253.36, 479.2) | 355.34 (253.56, 484.36) | -0.32 (-0.54, -0.10) |
| Chile | 5.91 (5.07, 6.93) | 6.68 (5.42, 8.2) | 103.19 (88.57, 120.98) | 521.78 (420.97, 644.18) | 0.78 (0.55, 1.00) |
| China | 1189.13 (1019.36, 1367.6) | 677.45 (558.88, 807.54) | 216.4 (185.51, 248.88) | 542.99 (426.05, 668.51) | -1.89 (-2.20, -1.58) |
| Colombia | 37.19 (32.91, 42.01) | 35.19 (27.26, 44.64) | 264.29 (233.89, 298.53) | 259.08 (182.56, 337.9) | 1.05 (0.68, 1.41) |
| Comoros | 0.44 (0.14, 0.65) | 0.76 (0.45, 1.01) | 253.12 (80.95, 374.87) | 111.39 (87.68, 138.77) | -0.63 (-1.30, 0.03) |
| Cook Islands | 0.02 (0.01, 0.02) | 0.02 (0.01, 0.03) | 249.41 (192.34, 323.11) | 702.59 (543.52, 867.09) | -2.01 (-2.31, -1.71) |
| Costa Rica | 2.21 (1.75, 2.78) | 5.22 (4.04, 6.58) | 172.4 (136.08, 216.45) | 408.9 (331.46, 511.23) | -0.29 (-0.54, -0.04) |
| Croatia | 1.23 (0.99, 1.52) | 0.88 (0.65, 1.14) | 67.74 (54.65, 83.85) | 383.59 (328.05, 459.25) | -0.26 (-0.37, -0.14) |
| Cuba | 8.7 (7.41, 10.28) | 7.93 (6.26, 9.88) | 178.85 (152.38, 211.31) | 568.96 (484.95, 663.53) | 0.54 (0.04, 1.04) |
| Cyprus | 0.17 (0.14, 0.22) | 0.25 (0.18, 0.33) | 56.49 (44.35, 71.89) | 352.04 (269.73, 449.59) | 0.05 (-0.13, 0.24) |
| Czech Republic | 2.97 (2.45, 3.68) | 1.98 (1.48, 2.61) | 79.94 (65.93, 99.05) | 215.05 (178.97, 259.78) | 1.64 (1.47, 1.82) |
| Democratic Republic of the Congo | 44.39 (35.21, 55.82) | 90.42 (71.44, 113.4) | 310.5 (246.27, 390.47) | 72.83 (57.64, 94.17) | 1.67 (1.47, 1.87) |
| Denmark | 0.87 (0.67, 1.16) | 0.88 (0.67, 1.14) | 45.68 (35.07, 60.76) | 189.37 (121.75, 270.49) | -0.28 (-0.56, 0.00) |
| Djibouti | 0.44 (0.31, 0.58) | 1.32 (0.88, 1.81) | 220.12 (155.17, 290.7) | 68 (50.72, 88.71) | 0.50 (0.26, 0.73) |
| Dominica | 0.12 (0.1, 0.14) | 0.17 (0.13, 0.21) | 420.1 (358.26, 492.01) | 298.71 (229.58, 380.75) | -1.41 (-1.86, -0.95) |
| Dominican Republic | 6.93 (5.95, 8.01) | 15.82 (11.34, 20.69) | 225.6 (193.7, 260.59) | 354.45 (254.77, 464.53) | 1.39 (1.24, 1.53) |
| Ecuador | 9.9 (8.83, 11.09) | 22.03 (17.58, 27.05) | 240.08 (214.3, 269.1) | 173.23 (147.77, 204.91) | -0.26 (-0.70, 0.19) |
| Egypt | 41.9 (31.2, 50.64) | 79.37 (56.9, 104.53) | 191.24 (142.39, 231.09) | 566.77 (420.58, 716.58) | -0.97 (-1.12, -0.82) |
| El Salvador | 5.9 (5.11, 6.86) | 18.87 (14.54, 24.16) | 282.8 (245.12, 329.04) | 267.01 (186.36, 349.92) | -0.70 (-0.78, -0.62) |
| Equatorial Guinea | 0.52 (0.38, 0.7) | 1.79 (1.18, 2.55) | 348.98 (250.84, 467.08) | 98.12 (80.99, 116.85) | -0.43 (-0.89, 0.02) |
| Eritrea | 3.24 (2.23, 4.32) | 8.62 (5.67, 12.8) | 279.5 (193.01, 373.44) | 62.55 (50.66, 77.31) | -0.52 (-0.70, -0.35) |
| Estonia | 0.34 (0.24, 0.46) | 0.4 (0.29, 0.53) | 59.09 (42.28, 81.79) | 82.24 (65.37, 103.65) | -0.25 (-0.45, -0.04) |
| Ethiopia | 79.36 (58.97, 95.8) | 95.45 (79.44, 115.31) | 425.4 (316.1, 513.56) | 221.73 (174.27, 279.27) | -0.93 (-0.99, -0.86) |
| Fiji | 1.32 (1.06, 1.63) | 1.95 (1.53, 2.4) | 408.97 (328.25, 504.94) | 305.42 (243.84, 375.15) | -2.26 (-2.89, -1.63) |
| Finland | 0.69 (0.53, 0.9) | 0.66 (0.5, 0.88) | 38.17 (28.95, 49.7) | 211.76 (158.36, 284.82) | 0.11 (-0.03, 0.25) |
| France | 9.34 (7.16, 12.2) | 7.36 (5.31, 9.97) | 42.45 (32.57, 55.48) | 375.43 (292.13, 472.92) | 0.73 (0.49, 0.98) |
| Gabon | 1.43 (1.04, 1.85) | 2.67 (1.88, 3.6) | 375.86 (271.78, 484.3) | 345.83 (272.42, 429.07) | -1.52 (-1.87, -1.17) |
| Gambia | 1.2 (0.83, 1.64) | 3.34 (2.4, 4.38) | 316.7 (220.67, 434.74) | 189.55 (146.73, 240.92) | -0.87 (-1.02, -0.73) |
| Georgia | 3.49 (2.92, 4.13) | 2.74 (2.27, 3.34) | 163.9 (136.98, 194.1) | 128.9 (101.04, 162.41) | 2.19 (1.82, 2.56) |
| Germany | 16.06 (12.45, 20.62) | 11.63 (8.87, 15.5) | 54.08 (41.92, 69.42) | 453.95 (373.01, 543.12) | -0.83 (-1.04, -0.62) |
| Ghana | 20.36 (14.54, 26.9) | 53.79 (37.75, 70.44) | 354.1 (252.93, 467.88) | 279.23 (219.15, 343.33) | 0.60 (0.46, 0.73) |
| Greece | 2.77 (2.26, 3.34) | 1.73 (1.38, 2.21) | 73.58 (60.24, 88.86) | 579.62 (378.76, 762.94) | 0.09 (-0.09, 0.26) |
| Greenland | 0.02 (0.01, 0.02) | 0.01 (0.01, 0.01) | 56.76 (42.85, 73.08) | 37.01 (26.73, 50.13) | 0.04 (-0.03, 0.11) |
| Grenada | 0.14 (0.12, 0.16) | 0.22 (0.18, 0.27) | 417.49 (355.72, 482.38) | 35.06 (27.22, 44.91) | -2.73 (-2.87, -2.60) |
| Guam | 0.15 (0.12, 0.18) | 0.28 (0.23, 0.34) | 229.21 (189.55, 277.37) | 225.73 (173.18, 286.95) | -3.12 (-3.84, -2.40) |
| Guatemala | 11.44 (10.04, 13.12) | 43.46 (34.9, 53.81) | 394.34 (345.88, 452.15) | 138.66 (109.73, 172.19) | 2.43 (2.02, 2.84) |
| Guinea | 9.5 (7.4, 12.39) | 20.8 (15.61, 27.45) | 439.78 (342.94, 573.77) | 338.33 (286.18, 398.75) | 0.72 (0.31, 1.13) |
| Guinea-Bissau | 2.41 (1.77, 3.15) | 4.29 (3.2, 5.58) | 642.67 (470.63, 839.74) | 33.39 (24.15, 45.2) | 0.15 (0.09, 0.21) |
| Guyana | 1.22 (1.02, 1.42) | 1.89 (1.45, 2.39) | 359.02 (298.5, 417.8) | 434.67 (317.76, 624.72) | 1.15 (1.01, 1.30) |
| Haiti | 10.8 (7.59, 14.88) | 21.95 (16.23, 30.5) | 444.36 (312.33, 612.31) | 104.35 (82.25, 131.85) | -0.53 (-0.86, -0.20) |
| Honduras | 5.86 (4.75, 7.07) | 12.35 (8.65, 16.79) | 339.41 (275.44, 409.69) | 524.77 (407.17, 666.32) | -0.79 (-1.00, -0.58) |
| Hungary | 3.44 (2.93, 4.05) | 2.24 (1.72, 2.85) | 93.06 (79.28, 109.54) | 357.61 (254.04, 477.86) | -0.12 (-0.28, 0.05) |
| Iceland | 0.03 (0.03, 0.05) | 0.04 (0.03, 0.05) | 32.78 (24.54, 44.14) | 64.15 (51.83, 79.46) | 1.79 (1.61, 1.98) |
| India | 922.91 (798.19, 1048.36) | 1532.11 (1322.32, 1760.68) | 270.83 (234.23, 307.64) | 773.11 (547.91, 1028.12) | -0.31 (-0.64, 0.02) |
| Indonesia | 329.6 (289.83, 372.18) | 406.31 (347.48, 486.45) | 422.24 (371.29, 476.79) | 377.36 (272.91, 485.71) | 0.32 (-0.04, 0.67) |
| Iran | 37.12 (31.81, 43.06) | 59.03 (48.42, 72.99) | 164.28 (140.78, 190.57) | 164.26 (119.06, 212.91) | 2.33 (2.22, 2.45) |
| Iraq | 18.97 (14.66, 24.28) | 46.5 (33.87, 60.65) | 288.5 (223.01, 369.35) | 427.83 (297.15, 566.85) | 1.48 (1.20, 1.75) |
| Ireland | 0.74 (0.56, 0.97) | 0.8 (0.59, 1.08) | 54.06 (40.69, 70.87) | 546.57 (410.66, 694.23) | 3.72 (3.38, 4.07) |
| Israel | 1.21 (0.96, 1.51) | 1.91 (1.51, 2.46) | 63.24 (50.02, 78.86) | 465.29 (366.54, 612.04) | -1.12 (-1.28, -0.97) |
| Italy | 11.77 (9.35, 14.64) | 6.75 (5.07, 8.84) | 55.14 (43.81, 68.57) | 98.12 (79.71, 120.48) | -0.71 (-1.07, -0.34) |
| Ivory Coast | 21.17 (16.2, 26.52) | 46.07 (31.99, 61.03) | 447.66 (342.47, 560.59) | 251.68 (187.65, 337.51) | -1.07 (-1.13, -1.01) |
| Jamaica | 2.03 (1.71, 2.42) | 4.48 (3.49, 5.64) | 206.11 (173.94, 246.14) | 584.79 (447.59, 740.22) | -0.08 (-0.29, 0.13) |
| Japan | 29.92 (25.31, 35.38) | 16.8 (13.08, 21.28) | 66.71 (56.44, 78.89) | 466.4 (342.78, 582.73) | -1.01 (-1.11, -0.91) |
| Jordan | 2.97 (2.5, 3.52) | 9.6 (7.73, 11.7) | 192.18 (162.11, 227.57) | 732.55 (564.48, 937.7) | -0.52 (-0.65, -0.38) |
| Kazakhstan | 18.36 (16.18, 21.05) | 14.62 (12.06, 17.68) | 270.22 (238.07, 309.74) | 36.91 (26.01, 50.44) | -0.22 (-0.33, -0.11) |
| Kenya | 16.3 (13.57, 19.92) | 50.49 (40.89, 61.45) | 186.24 (154.99, 227.56) | 223.4 (173.84, 280.88) | 0.39 (0.03, 0.75) |
| Kiribati | 0.21 (0.17, 0.26) | 0.45 (0.34, 0.59) | 684.73 (555.35, 841.63) | 422.41 (272.47, 669.86) | -2.34 (-2.68, -2.00) |
| Kuwait | 1.4 (1.17, 1.68) | 2.74 (1.99, 3.74) | 161 (134.13, 192.7) | 282.39 (211.64, 371.05) | 2.11 (1.83, 2.39) |
| Kyrgyzstan | 9.83 (8.57, 11.21) | 9.01 (7.62, 10.62) | 544.95 (475, 621.35) | 45.11 (33.11, 60.42) | 0.64 (0.41, 0.86) |
| Laos | 9.2 (6.78, 11.66) | 14.68 (10.79, 18.35) | 595.34 (438.62, 754.17) | 661.78 (468.34, 890.83) | 0.05 (-0.40, 0.51) |
| Latvia | 0.53 (0.38, 0.73) | 0.68 (0.52, 0.87) | 55.69 (39.8, 76.14) | 333.22 (274.03, 401.64) | -0.72 (-0.86, -0.58) |
| Lebanon | 2.31 (1.85, 2.83) | 3.65 (2.7, 4.76) | 191.72 (153.67, 235.04) | 192.64 (130.83, 273.28) | 0.91 (0.59, 1.24) |
| Lesotho | 1.25 (0.95, 1.62) | 3.84 (2.58, 5.29) | 181.54 (137.33, 235.29) | 271.53 (210.95, 341.02) | -0.17 (-0.24, -0.10) |
| Liberia | 3.06 (2.34, 3.9) | 7.76 (5.48, 10.71) | 458.27 (351.22, 584.81) | 558.62 (466.3, 662.6) | 0.01 (-0.08, 0.10) |
| Libya | 3.09 (2.43, 3.79) | 6.57 (4.97, 8.55) | 188.46 (148.48, 231.5) | 120.48 (83.05, 168.83) | -0.15 (-0.49, 0.20) |
| Lithuania | 0.81 (0.59, 1.1) | 0.78 (0.59, 1) | 58.33 (42.6, 78.84) | 55.95 (42.88, 72.15) | -0.53 (-0.59, -0.47) |
| Luxembourg | 0.08 (0.06, 0.1) | 0.11 (0.09, 0.14) | 55.07 (43.03, 69.44) | 351.86 (252.25, 460.13) | 1.64 (1.43, 1.85) |
| Madagascar | 14.68 (11.06, 18.27) | 24.95 (19.43, 31.62) | 324.41 (244.47, 403.73) | 391.16 (276.03, 539.44) | 0.75 (0.58, 0.93) |
| Malawi | 10.68 (7.87, 13.25) | 21.52 (16.13, 28.28) | 295.46 (217.63, 366.4) | 317.4 (244.07, 420.05) | 1.83 (1.52, 2.14) |
| Malaysia | 18.14 (15.65, 20.91) | 31.8 (25, 39.55) | 244.71 (211.12, 282.1) | 115.18 (96.1, 138.89) | 0.17 (-0.60, 0.95) |
| Maldives | 0.31 (0.25, 0.38) | 0.61 (0.5, 0.75) | 381.94 (309.59, 471.32) | 177.86 (131.46, 231.73) | 0.41 (0.30, 0.52) |
| Mali | 13.36 (10.24, 17.19) | 29.05 (20.73, 39.59) | 448.76 (344.15, 577.67) | 569.24 (493.79, 664.08) | 2.42 (2.21, 2.63) |
| Malta | 0.08 (0.06, 0.1) | 0.09 (0.07, 0.11) | 54.88 (43.33, 70.14) | 154.08 (114.31, 204.82) | 1.02 (0.34, 1.71) |
| Marshall Islands | 0.08 (0.07, 0.1) | 0.18 (0.13, 0.24) | 487.12 (399.5, 599.27) | 233 (188.7, 283.58) | 1.30 (0.95, 1.65) |
| Mauritania | 3.63 (2.93, 4.38) | 4.81 (3.26, 6.66) | 471.9 (381.47, 570.12) | 45.64 (34.8, 60.81) | -0.92 (-1.07, -0.76) |
| Mauritius | 1.97 (1.71, 2.27) | 3.22 (2.57, 3.95) | 395.29 (342.5, 454.16) | 380.36 (267.05, 522.72) | -0.44 (-0.66, -0.21) |
| Mexico | 114.26 (101.13, 130.64) | 285.65 (247.79, 333.24) | 320.57 (283.73, 366.52) | 381.7 (262.41, 515.67) | 0.78 (0.61, 0.96) |
| Micronesia (Federated States of) | 0.26 (0.2, 0.33) | 0.41 (0.24, 0.55) | 649.96 (503.42, 833.02) | 355.91 (240.93, 463.13) | 0.50 (0.44, 0.56) |
| Moldova | 1.18 (0.95, 1.49) | 1.65 (1.35, 2.05) | 67.64 (54.38, 85.46) | 540.15 (438.02, 650.8) | -0.84 (-0.97, -0.72) |
| Monaco | 0 (0, 0.01) | 0 (0, 0.01) | 41.93 (30.64, 55.82) | 231.41 (192.17, 281.97) | 2.52 (2.20, 2.84) |
| Mongolia | 5.55 (4.59, 6.65) | 5.54 (4.49, 6.93) | 628.12 (519.64, 752.75) | 525.01 (432.22, 619.4) | -1.35 (-1.47, -1.24) |
| Montenegro | 0.43 (0.37, 0.5) | 0.32 (0.26, 0.39) | 171.85 (146.42, 200.56) | 49.87 (36.81, 67.06) | 3.02 (2.80, 3.24) |
| Morocco | 18.67 (15.22, 22.57) | 30.96 (23.15, 41.64) | 179.44 (146.28, 216.87) | 431.2 (323.67, 569.06) | 0.14 (0.07, 0.20) |
| Mozambique | 10.94 (8.58, 14) | 36.2 (25.84, 46.5) | 239.06 (187.63, 305.93) | 230.72 (181.38, 286.96) | 1.44 (1.25, 1.63) |
| Myanmar | 116.26 (89.61, 146.4) | 120.78 (90.75, 153.41) | 686.45 (529.11, 864.41) | 193.52 (138.73, 254.85) | 0.12 (0.03, 0.21) |
| Namibia | 0.96 (0.64, 1.38) | 1.93 (1.24, 2.76) | 171.76 (114.16, 247.01) | 299.54 (202.76, 414.37) | 0.41 (0.25, 0.57) |
| Nauru | 0.03 (0.02, 0.03) | 0.04 (0.03, 0.05) | 674.06 (484.34, 859.8) | 141.69 (110.15, 181.81) | 0.06 (-0.09, 0.20) |
| Nepal | 17.74 (13.46, 22.41) | 33.88 (23.87, 44.18) | 242.97 (184.35, 306.91) | 340.18 (271.91, 429.08) | -0.72 (-0.83, -0.60) |
| Netherlands | 2.38 (1.81, 3.07) | 1.93 (1.42, 2.56) | 39.55 (29.97, 50.94) | 256.99 (221.8, 295.33) | 1.19 (0.98, 1.40) |
| New Zealand | 0.78 (0.64, 0.96) | 0.9 (0.73, 1.12) | 56.59 (46.01, 69.11) | 64.69 (51.6, 82.47) | -0.28 (-0.64, 0.08) |
| Nicaragua | 4.89 (4.27, 5.62) | 14.74 (11.78, 18.59) | 332.08 (290.31, 381.45) | 48.64 (37.27, 63.44) | -0.32 (-0.48, -0.17) |
| Niger | 10.75 (8.44, 13.66) | 26.18 (18.52, 34.79) | 387 (303.81, 491.64) | 80.59 (62.21, 103.99) | 0.36 (0.29, 0.43) |
| Nigeria | 97.07 (75, 129.02) | 211.05 (157.36, 283.03) | 285 (220.19, 378.82) | 449.68 (333, 573.18) | 0.18 (0.09, 0.26) |
| Niue | 0 (0, 0) | 0 (0, 0) | 448.21 (323.15, 596.64) | 191.52 (154.33, 233.5) | -0.87 (-1.12, -0.62) |
| North Korea | 26.27 (23.56, 29.31) | 7.35 (5.98, 9.08) | 124.72 (111.84, 139.12) | 256.52 (187.74, 340.68) | -3.58 (-3.88, -3.27) |
| North Macedonia | 1.04 (0.88, 1.23) | 1 (0.78, 1.25) | 127.73 (108.8, 150.79) | 464.87 (384.1, 557.86) | 3.81 (3.28, 4.34) |
| Northern Mariana Islands | 0.12 (0.09, 0.16) | 0.06 (0.05, 0.09) | 523.97 (373.6, 680.88) | 532.78 (352.35, 759.07) | 0.50 (0.32, 0.68) |
| Norway | 0.62 (0.47, 0.82) | 0.62 (0.47, 0.83) | 39.03 (29.55, 51.03) | 533.93 (398.52, 694.73) | -0.24 (-0.37, -0.12) |
| Oman | 0.66 (0.5, 0.85) | 2.88 (2.06, 3.92) | 82.06 (62.73, 105.36) | 625.88 (480.59, 814.97) | -0.40 (-0.56, -0.24) |
| Pakistan | 129.79 (100.5, 167.68) | 518.41 (384.69, 655.43) | 316.98 (245.44, 409.51) | 272.34 (210.76, 343.32) | 0.09 (-0.23, 0.41) |
| Palau | 0.05 (0.03, 0.06) | 0.06 (0.05, 0.08) | 660.37 (469.13, 864.53) | 301.1 (212.72, 420.44) | -0.30 (-0.51, -0.08) |
| Palestine | 1.97 (1.56, 2.5) | 4.43 (3.66, 5.36) | 257.16 (203.37, 326.03) | 274.52 (181.52, 392.11) | -0.19 (-0.39, 0.02) |
| Panama | 2.04 (1.71, 2.44) | 5.02 (3.85, 6.27) | 202.03 (169.05, 241.6) | 159.41 (119.89, 207.21) | 1.48 (1.26, 1.70) |
| Papua New Guinea | 4.21 (3.29, 5.1) | 12.51 (10.01, 15.71) | 254.28 (198.69, 307.96) | 55.41 (41.44, 72.49) | -0.43 (-0.95, 0.09) |
| Paraguay | 2.3 (1.92, 2.76) | 7.01 (5.41, 8.83) | 147.06 (122.46, 176.39) | 211.99 (175.13, 256.43) | -0.30 (-0.54, -0.06) |
| Peru | 21.8 (18.56, 25.22) | 30.77 (23.61, 39.12) | 245.78 (209.29, 284.39) | 98.86 (72.57, 132.33) | 0.17 (0.03, 0.30) |
| Philippines | 104.09 (93.81, 116.04) | 262.45 (223.69, 306.07) | 400.9 (361.31, 446.92) | 29.5 (22.24, 38.97) | -0.05 (-0.15, 0.04) |
| Poland | 21.88 (19.47, 24.92) | 9.2 (7.29, 11.9) | 151.32 (134.62, 172.37) | 488.42 (395.26, 583.69) | -0.32 (-0.41, -0.24) |
| Portugal | 3.45 (2.96, 4.04) | 1.73 (1.37, 2.15) | 91.17 (78.24, 106.81) | 925.2 (630.7, 1187.65) | 1.10 (1.03, 1.18) |
| Puerto Rico | 3.45 (2.96, 4.01) | 3.05 (2.37, 3.83) | 243.86 (209.6, 283.91) | 41.93 (31.49, 54.91) | 1.31 (1.04, 1.57) |
| Qatar | 0.25 (0.2, 0.33) | 2.07 (1.43, 2.9) | 107.47 (82.84, 140.34) | 520.19 (333.02, 709.09) | 1.84 (1.71, 1.98) |
| Republic of Congo | 3.87 (2.64, 5.39) | 6.34 (4.48, 8.85) | 407.73 (278.37, 568.11) | 520.52 (357.93, 703.7) | 1.62 (1.53, 1.71) |
| Reunion | 0 (0, 0) | 0 (0, 0) | 398.32 (276.03, 548.95) | 930.7 (696.1, 1199.88) | 1.06 (0.85, 1.27) |
| Romania | 15 (13.56, 16.74) | 5.86 (4.62, 7.41) | 172.62 (155.99, 192.6) | 235.72 (181.88, 297.08) | 0.74 (0.56, 0.92) |
| Russia | 117.95 (103.62, 136.29) | 68.04 (52.89, 87.31) | 202.68 (178.06, 234.19) | 165.78 (135.98, 204.98) | 0.10 (0.02, 0.17) |
| Rwanda | 10.07 (7.79, 12.63) | 12.84 (9.39, 16.91) | 368.73 (285.04, 462.4) | 49.09 (36.31, 65.4) | 0.51 (0.33, 0.69) |
| Saint Kitts | 0.09 (0.08, 0.1) | 0.09 (0.05, 0.13) | 516.92 (445.05, 601.58) | 287.33 (226.36, 361.93) | -1.65 (-2.06, -1.24) |
| Saint Lucia | 0.18 (0.16, 0.21) | 0.31 (0.25, 0.37) | 322.21 (278.72, 371.21) | 277.59 (188.76, 383.81) | 1.32 (1.05, 1.59) |
| Saint Vincent | 0.14 (0.12, 0.17) | 0.21 (0.17, 0.25) | 311.79 (265.37, 364.14) | 322.52 (230.26, 414.32) | 0.97 (0.74, 1.20) |
| Samoa | 0.25 (0.19, 0.34) | 0.43 (0.28, 0.59) | 371.75 (277.61, 513.16) | 490.27 (391.95, 604.99) | 2.06 (1.85, 2.27) |
| San Marino | 0 (0, 0) | 0 (0, 0.01) | 34.27 (24.58, 46.49) | 39.71 (29.9, 52.64) | -1.26 (-1.39, -1.13) |
| Sao Tome and Principe | 0.17 (0.09, 0.23) | 0.46 (0.35, 0.58) | 399.6 (210.55, 546.25) | 259.58 (198.44, 323.27) | -1.14 (-1.50, -0.77) |
| Saudi Arabia | 14.67 (11.18, 18.3) | 54.73 (42.06, 69.76) | 218.77 (166.66, 272.84) | 246.7 (197.84, 307.74) | 1.06 (1.02, 1.11) |
| Senegal | 12.1 (9.16, 15.8) | 23.4 (16.08, 31.61) | 438.91 (332.3, 572.94) | 227.95 (158.55, 329.27) | 0.34 (0.11, 0.57) |
| Serbia | 3.77 (3.22, 4.42) | 2.32 (1.85, 2.93) | 109.63 (93.5, 128.43) | 107.4 (84.46, 134.02) | -1.00 (-1.27, -0.72) |
| Seychelles | 0.09 (0.08, 0.1) | 0.17 (0.14, 0.21) | 283.72 (240.2, 333.94) | 40.35 (28.98, 55.62) | 0.51 (0.38, 0.65) |
| Sierra Leone | 4.47 (3.26, 5.9) | 12.38 (9.08, 16.45) | 327.35 (238.79, 432.43) | 319.97 (254.77, 393.64) | 0.51 (0.38, 0.63) |
| Singapore | 1.83 (1.61, 2.1) | 1.33 (1.08, 1.65) | 121.21 (106.62, 138.86) | 142.52 (112.99, 176.6) | -0.25 (-0.63, 0.12) |
| Slovakia | 2.21 (1.86, 2.61) | 1.44 (1.11, 1.86) | 107.78 (90.96, 127.66) | 149.38 (127.42, 173.18) | 0.12 (-0.12, 0.36) |
| Slovenia | 0.39 (0.29, 0.5) | 0.3 (0.21, 0.42) | 50.47 (37.92, 65.84) | 257.39 (195.03, 335.74) | -0.62 (-0.82, -0.41) |
| Solomon Islands | 1.23 (0.94, 1.6) | 1.84 (1.42, 2.27) | 959.93 (728.32, 1243.3) | 327.84 (245.73, 472.09) | 0.14 (0.08, 0.21) |
| Somalia | 7.87 (5.85, 10.47) | 24.89 (19.14, 32.94) | 311.88 (231.74, 414.72) | 51.08 (39.78, 64.72) | -1.22 (-1.44, -1.00) |
| South Africa | 59 (51.84, 66.05) | 82.83 (65.25, 102.76) | 371.05 (325.99, 415.37) | 242.37 (196.58, 297.24) | -0.73 (-1.48, 0.03) |
| South Korea | 24.41 (17.57, 32.97) | 25.77 (18.86, 34.23) | 295.7 (212.85, 399.36) | 217.17 (164.07, 282.44) | 1.76 (1.62, 1.90) |
| South Sudan | 4.95 (3.5, 6.71) | 7.79 (5.42, 11.26) | 211.87 (149.76, 287.57) | 395.15 (277.27, 517.41) | -0.51 (-0.69, -0.33) |
| Spain | 10.37 (8.79, 12.17) | 4.55 (3.53, 5.83) | 69.89 (59.24, 82.06) | 218.76 (172.57, 272.33) | -0.38 (-0.65, -0.12) |
| Sri Lanka | 23.31 (18.84, 27.13) | 23.09 (18.19, 29.09) | 315.16 (254.66, 366.72) | 298.23 (207.1, 448.98) | -0.25 (-0.40, -0.09) |
| Sudan | 14.54 (10.95, 18.97) | 33.24 (22.58, 47.16) | 190.61 (143.58, 248.7) | 548.74 (440.67, 679.43) | -0.45 (-0.53, -0.37) |
| Suriname | 0.61 (0.45, 0.72) | 1.26 (1.04, 1.52) | 378.13 (280.34, 444.79) | 396.86 (324.6, 476.24) | -0.26 (-0.48, -0.05) |
| Swaziland | 0.79 (0.63, 1) | 2.32 (1.51, 3.27) | 262.04 (207.62, 332.21) | 136.11 (112.29, 162.25) | 2.14 (1.53, 2.74) |
| Sweden | 1.01 (0.76, 1.34) | 0.94 (0.71, 1.24) | 34.43 (25.75, 45.71) | 314.62 (241.32, 396.49) | -0.67 (-0.87, -0.47) |
| Switzerland | 1.15 (0.85, 1.55) | 1.12 (0.8, 1.54) | 43.79 (32.28, 58.77) | 292.36 (204.8, 397.4) | 0.00 (-0.17, 0.17) |
| Syria | 18.79 (15.03, 22.81) | 12.31 (9.58, 15.47) | 390.08 (312.12, 473.58) | 334.3 (222.33, 484.57) | -0.19 (-0.49, 0.12) |
| Taiwan | 16.03 (13.98, 18.49) | 12.66 (9.52, 16.45) | 173.83 (151.51, 200.48) | 112.35 (80.4, 152.92) | -0.82 (-1.26, -0.38) |
| Tajikistan | 3.03 (2.57, 3.56) | 8.96 (7.22, 11.02) | 143.08 (121.33, 168.49) | 35.39 (26.48, 47.1) | 2.05 (1.74, 2.37) |
| Tanzania | 24.18 (19.37, 29.95) | 56.83 (43.07, 74.13) | 249.84 (200.06, 309.45) | 269.38 (128.9, 344.53) | 0.12 (0.02, 0.23) |
| Thailand | 127.57 (105.64, 161.54) | 79.59 (61.04, 100.85) | 492.5 (407.83, 623.66) | 36.61 (26.93, 48.71) | -0.42 (-0.51, -0.32) |
| Timor-Leste | 1.03 (0.68, 1.35) | 1.45 (0.69, 1.85) | 322.62 (213.17, 424.51) | 245.99 (177.39, 334.03) | -0.07 (-0.41, 0.27) |
| Togo | 5.29 (4.06, 6.76) | 12.03 (8.7, 15.48) | 386.08 (296.06, 492.81) | 92.8 (69.8, 119.24) | 0.31 (0.22, 0.39) |
| Tonga | 0.1 (0.08, 0.14) | 0.17 (0.12, 0.24) | 261.95 (203.65, 373.21) | 53.89 (39.72, 72.34) | 2.53 (2.26, 2.79) |
| Trinidad | 1.4 (1.2, 1.61) | 2.3 (1.7, 2.93) | 278.54 (239.38, 321.55) | 403.95 (271.84, 557.2) | -2.36 (-2.76, -1.95) |
| Tunisia | 5.03 (4.21, 6) | 7.63 (5.81, 9.83) | 145.89 (122.21, 174.04) | 228.35 (177.85, 289.45) | -0.05 (-0.22, 0.12) |
| Turkey | 49.76 (41.54, 61.59) | 47.2 (37.35, 58.61) | 201.26 (168.03, 249.1) | 981.54 (571.29, 1304.05) | -1.45 (-1.60, -1.30) |
| Turkmenistan | 6.06 (5.39, 6.83) | 10.61 (8.56, 13.1) | 393.91 (350.81, 443.91) | 249 (181.41, 324.79) | 0.71 (0.61, 0.81) |
| Tuvalu | 0.02 (0.01, 0.02) | 0.03 (0.02, 0.04) | 532.44 (407.17, 685.06) | 210.46 (173.53, 254.4) | 0.76 (0.58, 0.94) |
| Uganda | 12.56 (9.03, 16.62) | 39.74 (28.66, 53.97) | 195.76 (140.66, 259.02) | 64.96 (48.68, 85.72) | 0.27 (-0.09, 0.62) |
| UK | 10.83 (8.32, 13.95) | 11.34 (8.52, 14.85) | 51.94 (39.91, 66.88) | 221.06 (178.14, 271.74) | 1.49 (1.41, 1.56) |
| Ukraine | 10.51 (7.69, 14.08) | 20.59 (16.32, 25.51) | 55.34 (40.52, 74.15) | 260.95 (173.97, 358.93) | 0.13 (-0.11, 0.38) |
| United Arab Emirates | 3 (2.31, 4.09) | 18.94 (12.22, 30.03) | 313.32 (241.2, 427.21) | 170.53 (133.19, 215.93) | -0.51 (-0.59, -0.42) |
| Uruguay | 0.95 (0.8, 1.11) | 1.18 (0.98, 1.41) | 83.89 (70.65, 98.09) | 120.88 (93.21, 154.14) | 1.42 (0.85, 1.99) |
| USA | 106.97 (86.52, 131.74) | 126.71 (105.71, 152.79) | 104.95 (84.89, 129.25) | 95.65 (71.19, 126.32) | 0.16 (0.03, 0.28) |
| Uzbekistan | 34.2 (29.25, 40.97) | 74.9 (61.66, 88.36) | 398.18 (340.47, 476.97) | 299.9 (197.33, 445.13) | -0.48 (-0.58, -0.39) |
| Vanuatu | 0.19 (0.13, 0.26) | 0.68 (0.45, 0.9) | 329.38 (228.92, 450.51) | 43.16 (31.53, 58.07) | 0.28 (0.15, 0.40) |
| Venezuela | 15.5 (12.96, 18.43) | 33.46 (25.67, 42.17) | 193.43 (161.77, 230.01) | 51.22 (35.46, 71.02) | 1.19 (0.84, 1.54) |
| Vietnam | 84.07 (63.28, 109.49) | 110.15 (86.45, 135.44) | 294.75 (221.84, 383.86) | 258.44 (204.19, 324.15) | 4.07 (3.52, 4.62) |
| Virgin Islands | 0.11 (0.09, 0.13) | 0.09 (0.07, 0.12) | 281.65 (233.99, 336) | 413.03 (305.36, 573.78) | 0.35 (0.21, 0.49) |
| Yemen | 6.39 (4.08, 9.08) | 21.37 (15.49, 27.7) | 138.5 (88.48, 196.88) | 183.97 (142.52, 233.36) | -0.53 (-0.74, -0.32) |
| Zambia | 10.55 (8.71, 12.77) | 26.71 (20.47, 34.11) | 347.62 (286.98, 420.94) | 47.74 (35.95, 61.54) | -1.26 (-1.36, -1.16) |
| Zimbabwe | 6.83 (4.87, 10.47) | 18.5 (12.84, 27.85) | 172.35 (122.89, 264.41) | 213.96 (182.05, 249.84) | 0.97 (0.61, 1.34) |

Supplementary Table 4. Case number of incidence, death, and DALY in 1990 and 2019, and their percentage change (%), by global, 5 SDI regions, and 21 geographical regions.

| **Location name** | **Incidence (x100000)** | | | **Death (x100000)** | | | **DALY (x100000)** | | |
| --- | --- | --- | --- | --- | --- | --- | --- | --- | --- |
|  | **1990** | **2019** | **Percentage change (%)** | **1990** | **2019** | **Percentage change (%)** | **1990** | **2019** | **Percentage change (%)** |
| Global | 5.49 (4.06, 6.94) | 9.56 (7.04, 12.11) | 74.13 | 64.84 (60.45, 69.18) | 84.8 (77.5, 69.18) | 30.86 | 50.63 (45.78, 56.07) | 70.29 (62.04, 56.07) | 38.74 |
| High SDI | 0.59 (0.4, 0.8) | 0.76 (0.53, 1.01) | 28.81 | 2.47 (2.38, 2.56) | 2.65 (2.38, 2.56) | 7.29 | 2.8 (2.34, 3.37) | 3.17 (2.58, 3.37) | 13.21 |
| High-middle SDI | 1.22 (0.88, 1.57) | 1.64 (1.16, 2.15) | 34.43 | 10.29 (9.55, 11.13) | 7.32 (6.74, 11.13) | -28.24 | 8.58 (7.57, 9.71) | 7.4 (6.2, 9.71) | -13.75 |
| Middle SDI | 1.97 (1.45, 2.49) | 3.51 (2.55, 4.45) | 78.17 | 27.7 (25.73, 29.89) | 30.17 (27.98, 29.89) | 8.66 | 21.11 (19.05, 23.37) | 25.41 (22.44, 23.37) | 20.38 |
| Low-middle SDI | 1.27 (0.95, 1.57) | 2.46 (1.86, 3.1) | 93.7 | 16.4 (14.74, 18.11) | 27.67 (24.62, 18.11) | 68.29 | 12.34 (11.02, 13.82) | 21.36 (18.94, 13.82) | 73.17 |
| Low SDI | 0.44 (0.34, 0.55) | 1.18 (0.9, 1.47) | 168.18 | 7.94 (6.97, 8.93) | 16.92 (14.68, 8.93) | 112.85 | 5.77 (5.12, 6.43) | 12.91 (11.21, 6.43) | 123.57 |
| East Asia | 1.08 (0.76, 1.39) | 1.1 (0.74, 1.48) | 1.85 | 16.03 (13.78, 18.57) | 7.66 (6.53, 18.57) | -52.12 | 12.3 (10.59, 14.11) | 7.16 (5.93, 14.11) | -41.79 |
| South Asia | 1.38 (1.05, 1.71) | 2.54 (1.91, 3.2) | 84.06 | 14.77 (12.53, 16.84) | 28.69 (24.8, 16.84) | 94.56 | 11.21 (9.62, 12.82) | 21.58 (18.85, 12.82) | 91.96 |
| Southeast Asia | 0.57 (0.43, 0.71) | 1.08 (0.81, 1.35) | 89.47 | 11.35 (10.28, 12.62) | 13.23 (11.74, 12.62) | 16.81 | 8.34 (7.52, 9.36) | 10.78 (9.42, 9.36) | 28.3 |
| Central Asia | 0.14 (0.11, 0.18) | 0.26 (0.2, 0.32) | 85.71 | 1.16 (1.07, 1.28) | 1.77 (1.54, 1.28) | 52.59 | 0.91 (0.82, 1.02) | 1.42 (1.23, 1.02) | 56.04 |
| High-income Asia Pacific | 0.14 (0.1, 0.19) | 0.12 (0.08, 0.16) | -14.29 | 0.63 (0.59, 0.66) | 0.15 (0.14, 0.66) | -76.19 | 0.58 (0.52, 0.66) | 0.26 (0.21, 0.66) | -55.17 |
| Oceania | 0.01 (0.01, 0.01) | 0.02 (0.02, 0.03) | 100 | 0.11 (0.09, 0.13) | 0.25 (0.2, 0.13) | 127.27 | 0.09 (0.07, 0.1) | 0.2 (0.17, 0.1) | 122.22 |
| Australasia | 0.01 (0.01, 0.01) | 0.01 (0.01, 0.02) | 0 | 0.03 (0.02, 0.03) | 0.03 (0.03, 0.03) | 0 | 0.04 (0.03, 0.05) | 0.05 (0.04, 0.05) | 25 |
| Eastern Europe | 0.36 (0.25, 0.47) | 0.43 (0.31, 0.57) | 19.44 | 1.41 (1.36, 1.46) | 0.79 (0.7, 1.46) | -43.97 | 1.33 (1.15, 1.57) | 0.95 (0.75, 1.57) | -28.57 |
| Western Europe | 0.16 (0.1, 0.23) | 0.16 (0.1, 0.23) | 0 | 0.56 (0.54, 0.57) | 0.3 (0.29, 0.57) | -46.43 | 0.77 (0.62, 0.97) | 0.58 (0.44, 0.97) | -24.68 |
| Central Europe | 0.13 (0.09, 0.17) | 0.13 (0.09, 0.17) | 0 | 0.74 (0.72, 0.76) | 0.29 (0.25, 0.76) | -60.81 | 0.61 (0.55, 0.69) | 0.33 (0.27, 0.69) | -45.9 |
| High-income North America | 0.23 (0.15, 0.32) | 0.22 (0.15, 0.31) | -4.35 | 0.87 (0.84, 0.89) | 1.13 (1.06, 0.89) | 29.89 | 1.13 (0.91, 1.39) | 1.33 (1.11, 1.39) | 17.7 |
| Andean Latin America | 0.03 (0.02, 0.04) | 0.08 (0.06, 0.1) | 166.67 | 0.52 (0.47, 0.58) | 0.78 (0.62, 0.58) | 50 | 0.39 (0.35, 0.44) | 0.66 (0.54, 0.44) | 69.23 |
| Central Latin America | 0.24 (0.18, 0.31) | 0.62 (0.47, 0.77) | 158.33 | 2.27 (2.21, 2.33) | 5.23 (4.6, 2.33) | 130.4 | 1.99 (1.77, 2.27) | 4.54 (3.92, 2.27) | 128.14 |
| Caribbean | 0.04 (0.03, 0.05) | 0.08 (0.06, 0.11) | 100 | 0.46 (0.41, 0.53) | 0.76 (0.61, 0.53) | 65.22 | 0.38 (0.33, 0.44) | 0.64 (0.53, 0.44) | 68.42 |
| Tropical Latin America | 0.18 (0.13, 0.24) | 0.33 (0.24, 0.43) | 83.33 | 1.9 (1.82, 1.97) | 1.59 (1.51, 1.97) | -16.32 | 1.5 (1.36, 1.67) | 1.56 (1.34, 1.67) | 4 |
| Southern Latin America | 0.03 (0.02, 0.04) | 0.05 (0.03, 0.06) | 66.67 | 0.33 (0.3, 0.35) | 0.37 (0.33, 0.35) | 12.12 | 0.27 (0.24, 0.31) | 0.34 (0.29, 0.31) | 25.93 |
| Eastern Sub-Saharan Africa | 0.1 (0.07, 0.13) | 0.26 (0.18, 0.33) | 160 | 2.96 (2.43, 3.39) | 5.42 (4.46, 3.39) | 83.11 | 2.13 (1.8, 2.43) | 4.2 (3.56, 2.43) | 97.18 |
| Southern Sub-Saharan Africa | 0.08 (0.06, 0.1) | 0.14 (0.11, 0.18) | 75 | 0.98 (0.88, 1.12) | 1.52 (1.21, 1.12) | 55.1 | 0.7 (0.62, 0.79) | 1.13 (0.92, 0.79) | 61.43 |
| Western Sub-Saharan Africa | 0.19 (0.15, 0.24) | 0.59 (0.45, 0.72) | 210.53 | 3.47 (2.86, 4.25) | 7.77 (6.03, 4.25) | 123.92 | 2.47 (2.06, 2.95) | 5.86 (4.75, 2.95) | 137.25 |
| North Africa and Middle East | 0.35 (0.25, 0.45) | 1.24 (0.9, 1.57) | 254.29 | 3.37 (3.01, 3.75) | 5.21 (4.25, 3.75) | 54.6 | 2.83 (2.5, 3.2) | 5.32 (4.36, 3.2) | 87.99 |
| Central Sub-Saharan Africa | 0.03 (0.02, 0.04) | 0.1 (0.07, 0.12) | 233.33 | 0.94 (0.75, 1.13) | 1.85 (1.39, 1.13) | 96.81 | 0.67 (0.55, 0.8) | 1.39 (1.1, 0.8) | 107.46 |

Supplementary Table 5. Proportion (%) of 7 attributable risk factors of early-onset chronic kidney disease (CKD) disability-adjusted life year (DALY) in 2019 for both sex, men, and women (A) including 7 factors, and excluding (B) high fasting plasma glucose and high systolic blood pressure.

| **Risk factors** | **Sex** | **Global (%)** | **SDI region (%)** | | | | |
| --- | --- | --- | --- | --- | --- | --- | --- |
|  |  |  | **High SDI** | **High-middle SDI** | **Low SDI** | **Low-middle SDI** | **Middle SDI** |
| High systolic blood pressure | Female | 24.8 | 22.62 | 27.09 | 24.21 | 24.17 | 25.23 |
| High systolic blood pressure | Male | 30.76 | 34.47 | 35.96 | 25.89 | 29.47 | 32.45 |
| High systolic blood pressure | Both | 28.03 | 28.85 | 31.92 | 25.16 | 27.02 | 29.12 |
| High fasting plasma glucose | Female | 17.59 | 16.47 | 17.79 | 12.85 | 14.86 | 22.26 |
| High fasting plasma glucose | Male | 18.85 | 17.33 | 22.1 | 10.85 | 15.18 | 25.38 |
| High fasting plasma glucose | Both | 18.27 | 16.92 | 20.14 | 11.73 | 15.03 | 23.94 |
| High body-mass index | Female | 8.34 | 16.07 | 10.17 | 5.85 | 7.17 | 9.01 |
| High body-mass index | Male | 7.3 | 16.21 | 10.27 | 3.42 | 6.26 | 8.28 |
| High body-mass index | Both | 7.78 | 16.14 | 10.23 | 4.48 | 6.68 | 8.62 |
| Low temperature | Female | 2.62 | 3.27 | 2.77 | 2.78 | 2.52 | 2.51 |
| Low temperature | Male | 3.15 | 3.89 | 3.87 | 2.92 | 2.91 | 3.17 |
| Low temperature | Both | 2.91 | 3.6 | 3.37 | 2.86 | 2.73 | 2.86 |
| Diet high in sodium | Female | 1.86 | 1.5 | 2.97 | 1.23 | 1.62 | 2.11 |
| Diet high in sodium | Male | 2.72 | 2.67 | 4.86 | 1.17 | 2.49 | 3.12 |
| Diet high in sodium | Both | 2.33 | 2.12 | 4 | 1.19 | 2.09 | 2.65 |
| Lead exposure | Female | 0.49 | 0.09 | 0.22 | 0.74 | 0.69 | 0.33 |
| Lead exposure | Male | 0.71 | 0.15 | 0.4 | 0.84 | 1.01 | 0.57 |
| Lead exposure | Both | 0.61 | 0.12 | 0.32 | 0.8 | 0.86 | 0.46 |
| High temperature | Female | 0.57 | 0.4 | 0.18 | 0.69 | 0.82 | 0.43 |
| High temperature | Male | 0.63 | 0.55 | 0.22 | 0.69 | 0.9 | 0.49 |
| High temperature | Both | 0.6 | 0.48 | 0.2 | 0.69 | 0.86 | 0.46 |

Supplementary Figure 1. Trends of age-specific (A) incidence rate, (B) death rate, (C) DALY rate of early-onset CKD according to five age-groups, by Global and SDI regions. DALY, disability-adjusted life year; CKD, chronic kidney disease.


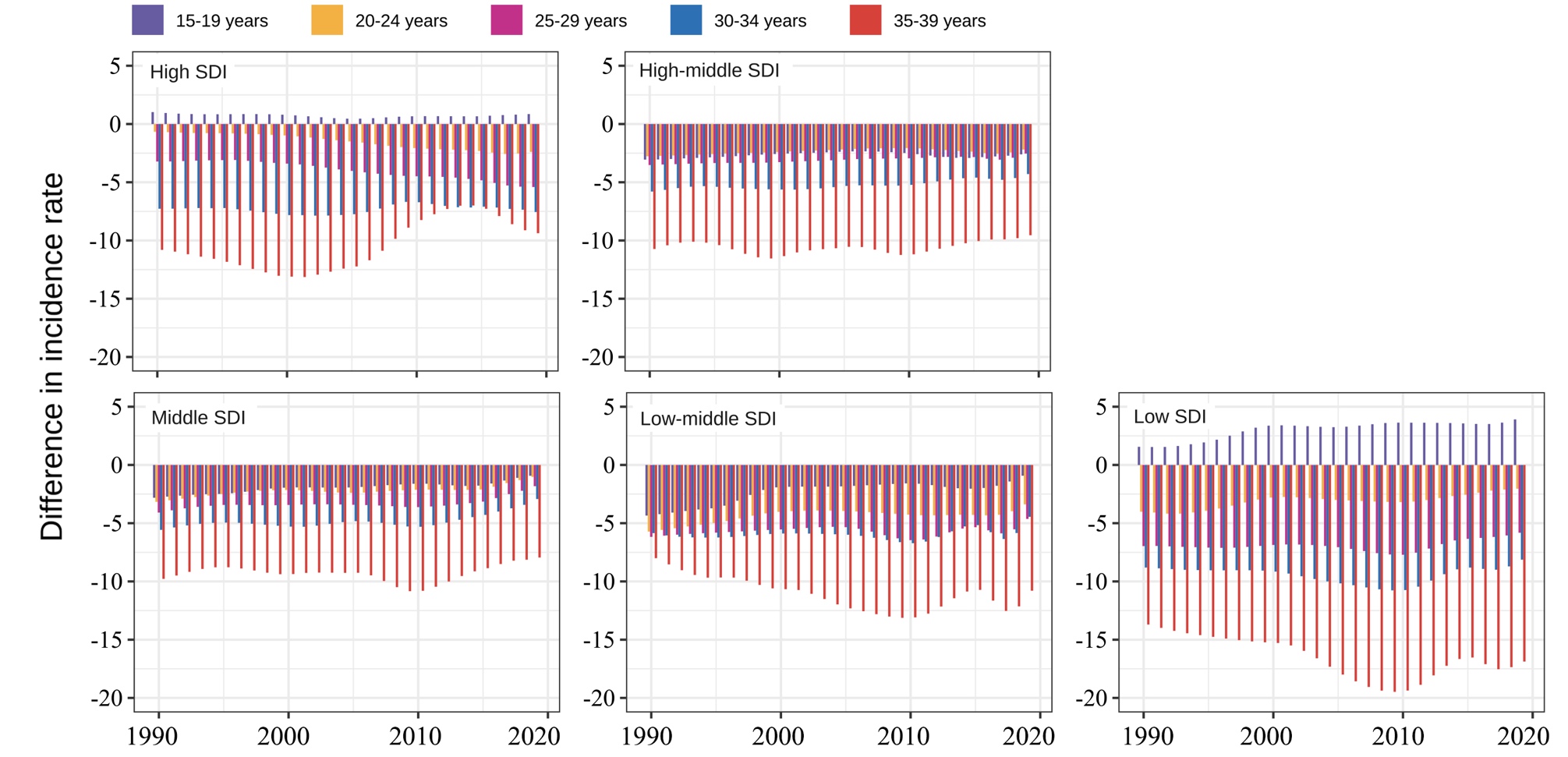


Supplementary Figure 2. The difference in incidence rate between men and women by age groups and SDI regions, from 1990 to 2019. The difference was calculated using the age-specific rate in males minus that in females, with a difference higher than 0 meaning men have higher rates. DALY, disability-adjusted life year.


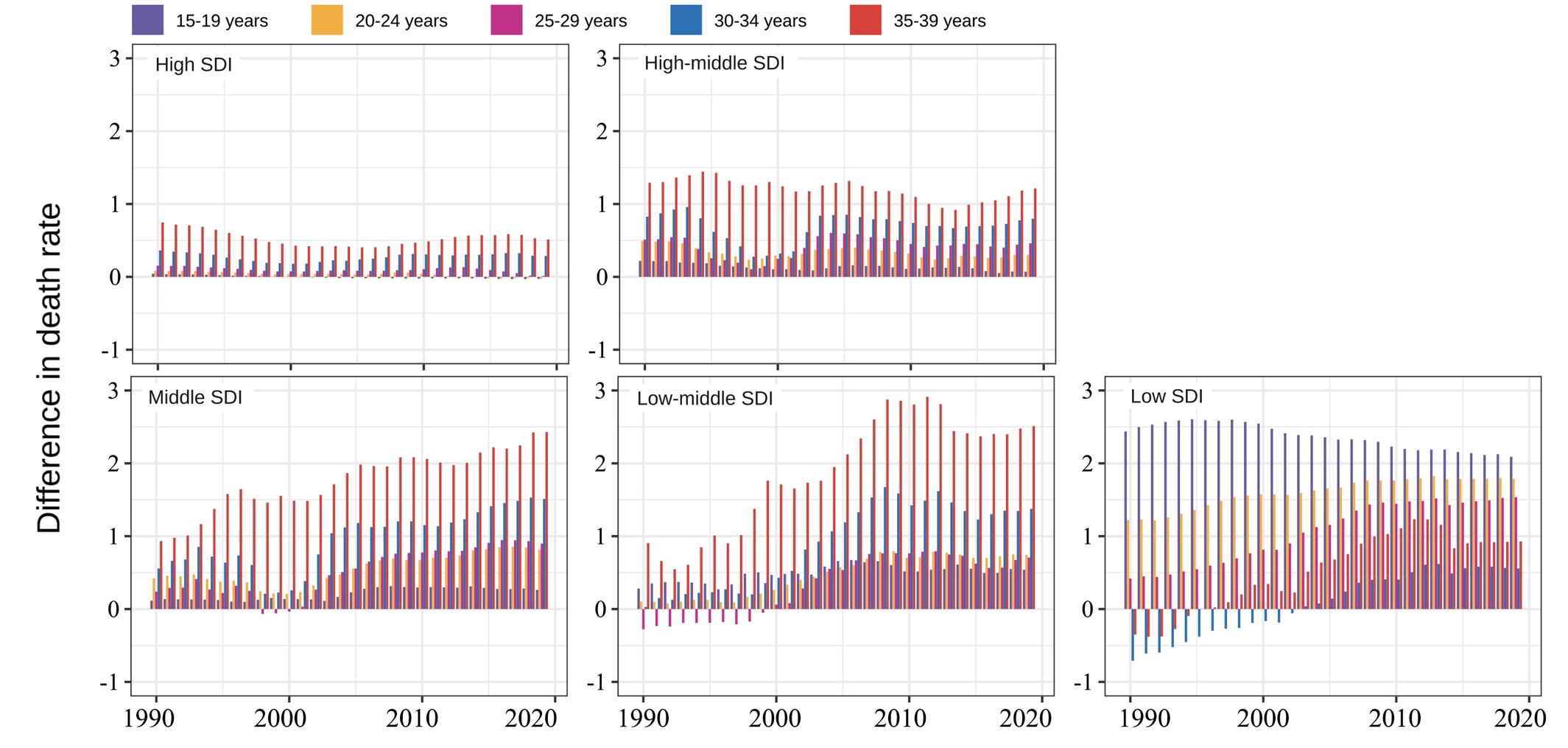


Supplementary Figure 3. The difference in death rate between men and women by age groups and SDI regions, from 1990 to 2019. The difference was calculated using the age-specific rate in males minus that in females, with a difference higher than 0 meaning men have higher rates. DALY, disability-adjusted life year.


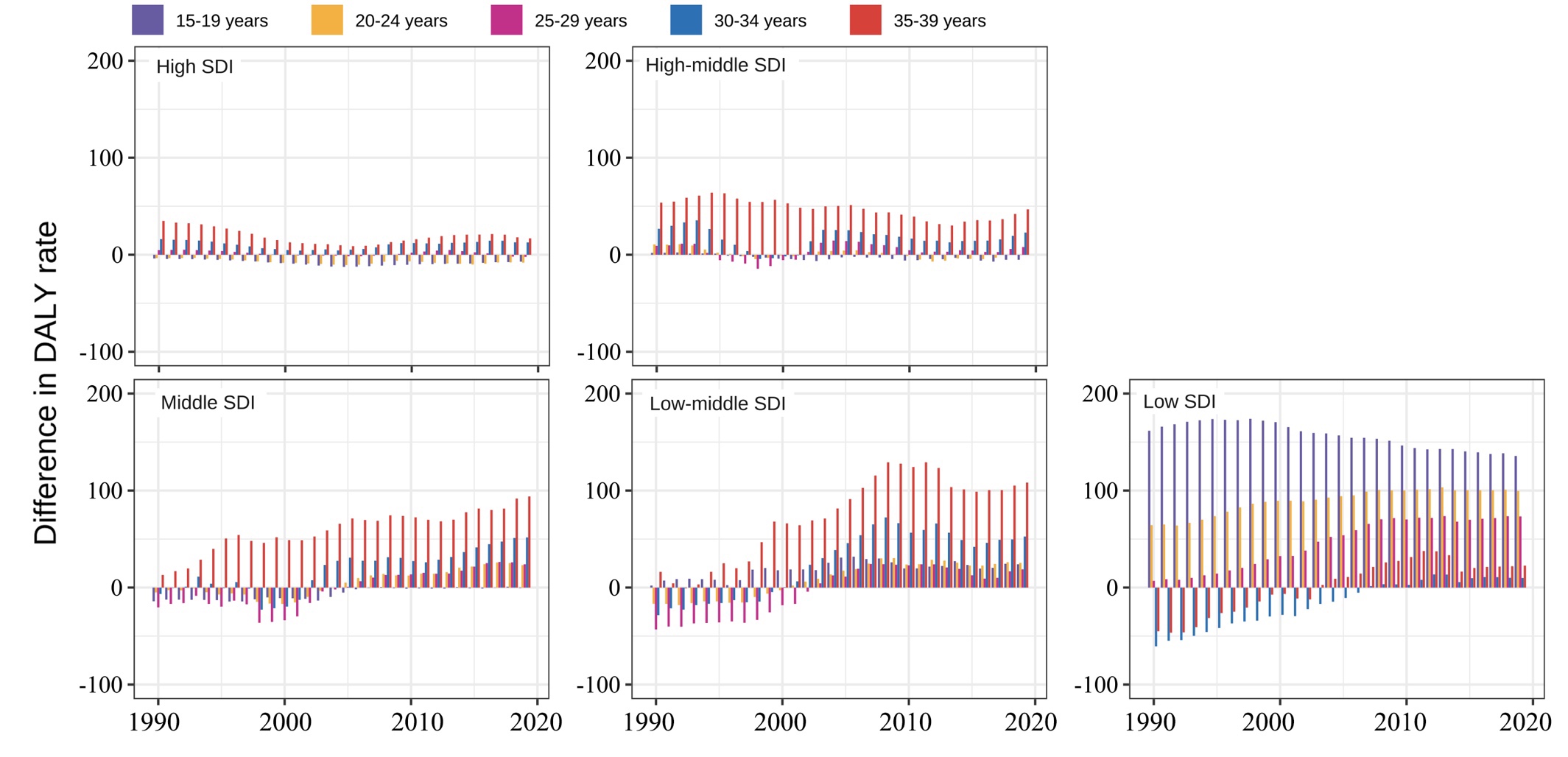


Supplementary Figure 4. The difference in DALY rate between men and women by age groups and SDI regions, from 1990 to 2019. The difference was calculated using the age-specific rate in males minus that in females, with a difference higher than 0 meaning men have higher rates. DALY, disability-adjusted life year.

Supplementary Figure 5. Temporal trend of age-specific (A) incidence, (B) death, and (C) DALY rates (both sex) for the early onset CKD by global, SDI regions, and age-groups, respectively, from 1990 to 2019. DALY, disability-adjusted life year; CKD, chronic kidney disease.

Supplementary Figure 6. Temporal trend of age-specific (A) incidence, (B) death, and (C) DALY rates (female and male) for the early onset CKD by global, SDI regions, and age-groups, respectively, from 1990 to 2019. EAPC, estimated annual percentage change;DALY, disability-adjusted life year; CKD, chronic kidney disease.

Supplementary Figure 7. EAPCs of age-specific (A) incidence, (B) death, and (C) DALY rates (Both sex, female, and male) for the early onset CKD by global, SDI regions, and age-groups, respectively, from 1990 to 2019. EAPC, estimated annual percentage change; DALY, disability-adjusted life year; CKD, chronic kidney disease.

**
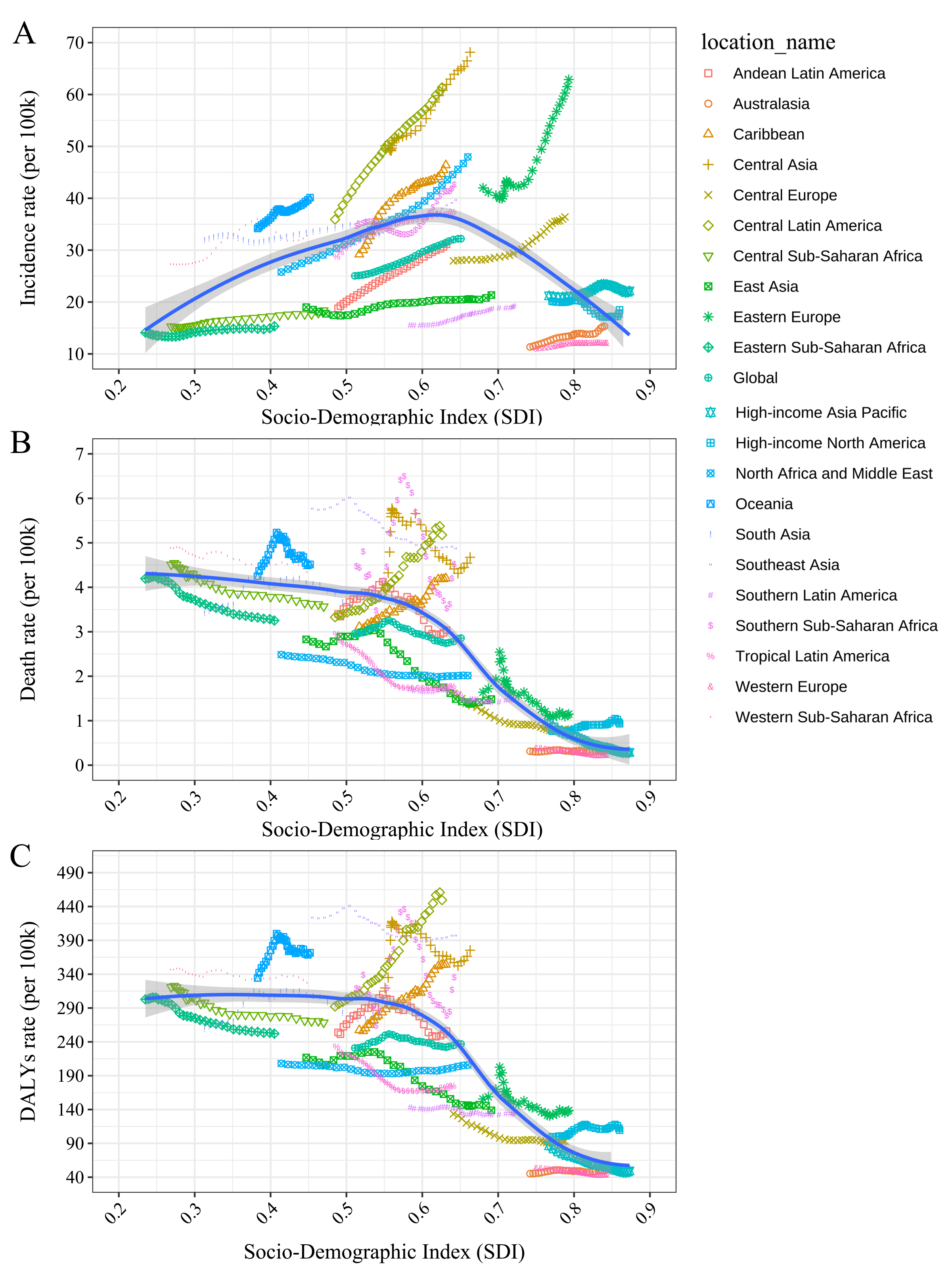
**

Supplementary Figure 8. The relationship between age-specific (A) incidence rate, (B) death rate, and (C) DALY rate of early-onset CKD and SDI (1990–2019) across 21 geographical regions. For each region, points from left to right depict estimates from each year from 1990 to 2019. Solid blue line and stark area shows expected values across the spectrum of the SDI. DALY, disability-adjusted life year; CKD, chronic kidney disease; SDI, socio-demographic index.

Supplementary Figure 9. Proportion of early-onset CKD DALYs attributable to 7 risk factors classified by sex, in the five age-groups, globally in 2019. DALY, disability-adjusted life year; CKD, chronic kidney disease.

Supplementary Figure 10. Proportion of attributable risk factors of early-onset CKD DALY in 1990 and 2019 for men and women (A) including 7 factors, and excluding (B) high fasting plasma glucose and high systolic blood pressure. DALY, disability-adjusted life year; CKD, chronic kidney disease.
